# Plasmonic modulator enabling kilometer-range high-throughput sub-THz links for radio access networks

**DOI:** 10.1038/s41467-026-72053-z

**Published:** 2026-04-18

**Authors:** Boris Vukovic, Laurenz Kulmer, Tobias Blatter, Yannik Horst, Marcel Destraz, Wolfgang Heni, Stefan M. Koepfli, Hande Ibili, Michael Baumann, Yuriy Fedoryshyn, Jasmin Smajic, Sarperi Luciano, Juerg Leuthold

**Affiliations:** 1 ETH Zurich, Institute of Electromagnetic Fields (IEF), Zurich, Switzerland; 2 Polariton Technologies, Adliswil, Switzerland; 3 ZHAW, Institute of Signal Processing and Wireless Communications (ISC), Winterthur, Switzerland

**Keywords:** Fibre optics and optical communications, Microwave photonics, Electrical and electronic engineering, Integrated optics

## Abstract

Future radio access networks must accommodate growing mobile data traffic. Capacity between mobile devices and remote radio units (RRUs) must increase by using higher wireless carrier frequencies. Higher frequencies reduce reach, requiring RRU densification and high-capacity front- and backhaul connections. Optical fiber offers high throughput, but deployment can be expensive or unfeasible. Here we show an all-photonic sub-THz wireless link at 226 GHz over 1400 m, achieving a record-high net-rate-distance product of 214.2 Gbit s^−1^ km. Broadband photonic and plasmonic components enabled flat frequency response at high speeds. A novel dual-sideband receiver increased signal-to-noise ratio by 2 dB. We assessed power variations due to atmospheric turbulence; the scintillation index remained below 0.019 under strong turbulence, confirming sub-THz link resilience. A theoretical comparison with free-space optical links highlights the turbulence resistance. In this work, we show sub-THz links offer high capacity, resilience to weather and turbulence, and cost-effective deployment for wireless front- and backhaul.

## Abstract (web summary)

The authors demonstrate a 226-GHz all-photonic wireless link over 1.4 km, achieving a record-high net-rate-distance product of 214.2 Gbits⁻¹ km using broadband photonic/plasmonic components and a dual-sideband receiver that boosts SNR by 2 dB, showing strong turbulence resilience and exciting sub-THz deployment potential.

## Introduction

In future mobile radio access networks (RAN), the links between the user end (UE) and remote radio unit (RRU) need to increase in capacity and therefore will extends beyond traditional sub-6 GHz frequencies to higher millimeter-wave, reaching up to 95 GHz^1^. While these higher frequencies offer more bandwidth, they also come with reduced range, requiring a denser deployment of RRUs to maintain coverage. Connections between the RRUs and a centralized baseband unit (BBU) are typically established using optical fiber, as it supports high data rates exceeding 100 Gbit s^−1^. However, deployment of fiber can be very expensive or unfeasible, such as in urban areas and areas with difficult terrain. To circumvent these issues, one may resort to high-capacity wireless-based communication such as free-space optical (FSO) links. FSO links typically operate at visible and near-IR wavelengths^2^ between 750 nm and 1600 nm. The very low atmospheric attenuation in clear weather conditions and low beam divergence makes them suitable for high data rates and long-distance links, they are even considered for earth-to-satellite communication^3^. Yet, FSO links can be strongly degraded by atmospheric turbulence effects and adverse weather, especially fog^4^. A solution offering sufficient bandwidth to transmit the high capacity signals as well as robustness against adverse weather and atmospheric turbulences^5^ are wireless links operating in the E/W (60–110 GHz) and in the sub-THz band (100–300 GHz). Demonstrations in the E/W band have achieved channel line rates of 96 Gbit s^−1^ over 4.6 km^6^ and 32 Gbit s^−1^ over 28.8 km^7^. In case even higher data rates above 100 Gbit s^−1^ are required, sub-THz frequencies offer more bandwidths. Unlike fiber, these sub-THz links can be rapidly deployed permanently or on a temporary basis to enhance network capacity in specific areas during events such as emergency situations. Furthermore, in line-of-sight conditions, a direct high-speed connection can be established between the user end and a sub-THz RRU. Smart reflecting surfaces can be employed to enable non-line-of-sight communication by redirecting the sub-THz beam around obstacles and performing real-time beam steering^8^. Depending on the transmitter and receiver technology, sub-THz links can be categorized into all-electronic, photonic-electronic, and all-photonic types.

All-electronic links use electronic mixers based on III-V materials such as InP or GaAs for signal conversion at the transmitter and the receiver^9^ and have demonstrated line rates of 96 Gbit s^−1^ over 40 m^10^, 64 Gbit s^−1^ over 850 m^11^, and 10 Gbit s^−1^ over 5.8 km^12^. While electronic transmitters offer higher output powers, they introduce non-linear distortions^13^ and have narrow bandwidths, which limit the capacity.

Even higher data rates are achieved using photonic-electronic links, which are enabled by high-speed photodetectors. The most used photodetector is the uni-traveling carrier photodiode (UTC-PD), which combines high bandwidth and output power in the milliwatt range^14,15^. Such transmitters leverage well-established optical communication transmitter technology, allowing for high-bandwidth transmission^9^. Recently, many photonic-electronic links have demonstrated line rates exceeding 100 Gbit s^−1^ at distances up to 214 m^13,16–23^. Data rates as high as 1056 Gbit s^−1^ over 3.1 m have been demonstrated using polarization multiplexing and MIMO schemes^17^. Long-range demonstrations achieved line rates of 19.6 Gbit s^−1^ over 4.6 km^24^ and 50 Gbit s^−1^ over 850 m^25^. While photonic-electronic links enable integration with fiber-optic networks at the transmitter, the limited bandwidth of the electronic receiver and the need for down-mixing to baseband complicate integration on the receiver side. This issue may be solved by all-photonic links. The key components enabling direct conversion between the sub-THz and the optical domain are high-speed photodetectors at the transmitter side and high-speed electro-optic modulators at the receiver side. While UTC-PDs with bandwidths in excess of 300 GHz have been available for many years, so far, the lack of modulators with sufficient bandwidth has prevented a direct up-mixing of the sub-THz signals to the optical domain. Recently, direct up-mixing at 152 GHz was achieved by employing thin-film lithium niobate (TFLN) modulators, demonstrating 80 Gbit s^−1^ back-to-back transmission^26^. While TFLN allow for low optical insertion losses, the electro-optic bandwidth drops beyond 100 GHz due to the difficulty of phase matching the RF and optical wave. Plasmonic modulators on the other hand have brought up a modulator solution offering bandwidths in excess of 500 GHz^27^ and most recently up to 1 THz^28^ that in addition requires low driving voltages and small footprint^29^. Such plasmonic-based up-mixer offer larger bandwidths than electronic receivers and integrate seamlessly with fiber-optic networks. This concept is first introduced by Salamin et al.^30^ and has meanwhile led to demonstrations showing 50 Gbit s^−1^ over 16 m^31^, and 240 Gbit s^−1^ over 5 m and 192 Gbit s^−1^ over 115 m^32^. However, a sub-THz wireless link combining net rates above 100 Gbit s^−1^ and distances above 1 km has not yet been shown.

In this work, we present an all-photonic sub-THz wireless link at 226 GHz over a distance of 1400 m, achieving a line rate of 184 Gbit s^−1^, an achievable information rate of 158.7 Gbit s^−1^, and a net rate of 153.2 Gbit s^−1^ - representing, to our knowledge, the highest net-rate-distance product above 100 GHz carrier frequency reported to date (see Table 1 and Fig. 2 below). The receiver utilizes direct conversion through an organic-plasmonic Mach-Zehnder modulator (MZM). Compared to prior art, a dual-sideband receiver scheme that provides a signal-to-noise ratio (SNR) gain of up to 2 dB^33^ has been employed. We report outdoor measurements of atmospheric turbulence effects on a high-data-rate sub-THz link, with scintillation indices staying below 0.019 even under strong turbulence. These results confirm the known resilience of sub-THz links to turbulence^34,35^ and, unlike many lab-based studies, provide valuable evidence to the community that sub-THz links perform reliably under real-world outdoor conditions over practical distances exceeding 1 km. All-photonic sub-THz links could provide a cost-effective and easily integrable alternative to fiber for the dense radio access networks of the future.

**Table 1:** State-of-the-art sub-THz-band (100–300 GHz), E-band (60–90 GHz) and W-band (75–110 GHz) links sorted by net-rate-distance product

Type^a^	Net-rate-distance product per polarization (Gbit s^−1^ km)	Net rate per polarization (Gbit s^−1^)	Distance (m)	Carrier frequency (GHz)	BER (pre-FEC)	FEC Details (Type / Overhead / Threshold)	Technology TX/RX	Modulation Format	Year	Author	Reference
P-E	0.715	255	2.8	350	2.4E-2	SD-FEC / 20% / 2.7E-2	UTC-PD/SBD	PS-64QAM-OFDM	2020	Jia et al.	^18^
All-P	0.98	196	5	231	4.6E-2	SD-FEC^c^ / 20% / -	UTC-PD/PM	QPSK, 16QAM	2022	Horst et al.	^32^
P-E	1.18	381	3.1	126, 150	<4E-2	SD-FEC / 27% / 4E-2	UTC-PD/D-Band Mixer	64QAM-PS5.5	2019	Li et al.	^17^
P-E	1.40	131	10.7	400	2.4E-2	SD-FEC / 20% / 2.7E-2	UTC-PD/SBD	16QAM	2022	Jia et al.	^20^
P-E	1.87	93.5	20	238	4.5E-3	HD-FEC / 7% / 4.5E-3	UTC-PD/InGaAs MMIC	16QAM	2013	Koenig et al.	^16^
P-E	8.68	167	52	300	6.0E-3	SD-FEC / 20% / 2.7E-2	UTC-PD/GaAs MMIC	16QAM	2024	Dittmer et al.	^13^
P-E	12.7	115	110	300	1.9E-3	SD-FEC / 15% / 1.25E-2	UTC-PD/SBD	16QAM	2020	Harter et al.	^19^
All-P	18.9	164	115	231	3.2E-2 (Offline)	SD-FEC^c^ / 17% / -	UTC-PD/PM	QPSK, 16QAM	2022	Horst et al.	^32^
P-E	19.3	96.7	200	300	2E-2	SD-FEC / 20% / 2E-2	UTC-PD/HM	PDM-QPSK	2024	Cai et al.	^21^
P-E	20.4	102	200	300	-	SD-FEC^c^ / 25% / -	UTC-PD/	PS-16QAM DP-OFDM	2024	Tong et al.	^22^
P-E	43.7	204	214	300	3.8E-3	HD FEC^b^ / - / 3.8E-3	UTC-PD/SHM	32QAM	2024	Maekawa et al.	^23^
All-E	50.8	59.8	850	240	7.9E-5	HD FEC^b^ / 7% / 4.5E-3	GaAs HEMT MMIC/”	QPSK	2015	Kallfass et al.	^11^
All-E	58.0	10	5800	120	-	HD FEC / 6% / -	InGaAs/InP HEMT MMIC/”	ASK	2010	Hirata et al.	^12^
P-E	90.3	19.6	4600	135	8.6E-3	SD-FEC / 15% / 1E-2	-	OFDM-16QAM	2022	Li et al.	^24^
P-E	384	83.5	4600	87.5	<2.4E-2	SD-FEC / 15% / 2.4E-2	PD/E-Band Mixer	PDM-16QAM	2023	Li et al.	^6^
All-P	746	27.8	26800	76.0	2.0E-2	SD-FEC / 15% / 2E-2	PD/Phase Modulator	QPSK	2024	Liu et al.	^7^
All-P	214	153	1400	230	3.8E-2	SD-FEC^c^ / 20% / -	UTC-PD/Plas. Modulator	QPSK, 8QAM, 16QAM	2024		This work

^a^The type of the link is indicated by All-E (all-electronic), P-E (photonic-electronic), and All-P (all-photonic). ^b^On-line digital signal processing (DSP) ^c^Forward error correction (FEC) type and overhead determined by normalized generalized mutual information (NGMI)

The content of the paper is an extended version of results first presented at the Optical Fiber Communications (OFC) conference 2024^36^.

## Results

### The case for Sub-THz communications

**Fig. 1:**
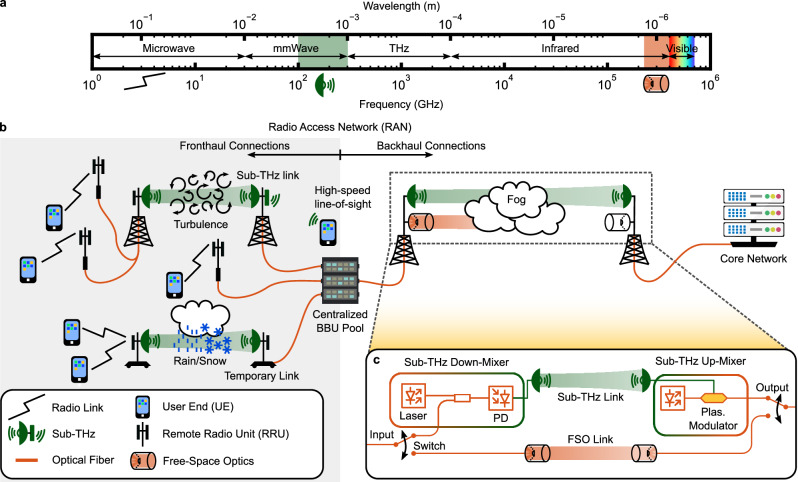
High-capacity sub-THz front- and backhaul connections for radio access networks as envisioned for future mobile communications. **a** Location of the sub-THz (green) and free-space optical (FSO) (red) bands in the electro-magnetic spectrum. **b** Vision of the radio access networks (RAN) with sub-THz wireless front- and backhaul connections. Remote radio units (RRUs) are connected through a centralized baseband unit (BBU) pool, which performs digital signal processing and network coordination for multiple cells. Sub-THz links offer not only high bandwidths but also robustness against adverse weather like rain, snow, fog, and atmospheric turbulence. **c** Scenario, with a sub-THz and FSO hybrid link which can be seamlessly switched to ensure reliable communication under all weather conditions. The all-photonic sub-THz link allows seamless integration with the fiber link and is enabled by employing a high-speed photodetector (PD) and a plasmonic modulator that offer bandwidths exceeding 500 GHz.

Sub-THz frequencies, positioned between microwave and optical bands, are located between 100 GHz and 300 GHz and belong to the upper part of the mmWave band (Fig. 1a, green-marked band). The vision of this work is depicted in Fig. 1, where the RAN integrates various communication systems, including fiber-based, radio, free-space optical (FSO), and sub-THz systems. Sub-THz channels are crucial because they can, unlike microwave links, match the bandwidth of the fiber backbone. While FSO can achieve high bandwidths, its performance is highly sensitive to weather conditions like fog and turbulence. Sub-THz frequencies may offer a balance of high throughput and resilience towards adverse weather, making them an ideal complement to FSO to ensure high-capacity, reliable wireless communications.

A high-speed sub-THz down- and up-mixer enables a seamless transition between the optical and sub-THz domains, as shown in Fig. 1c. During adverse weather conditions that disrupt free-space optical (FSO) links, the optical signal at the transmitter can be rerouted through the sub-THz down-mixer and sent over a sub-THz carrier. At the receiver, the signal is directly up-mixed back into the optical domain using a high-speed plasmonic modulator. This seamless switching capability allows the optical and sub-THz links to complement each other in a confluent way, effectively addressing the limitations of each technology.

**Fig. 2:**
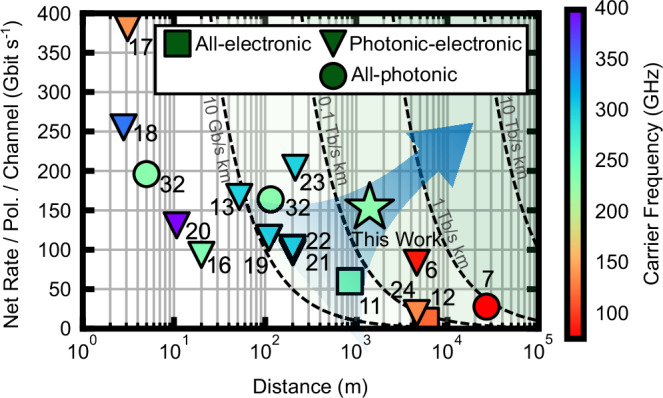
State-of-the-Art THz-band (0.3–10 THz), sub-THz-band (100–300 GHz), E-band (60–90 GHz) and W-band (75–110 GHz) links. Net rate per polarization and channel versus transmission distance of state-of-the-art wireless links. The color represents the carrier frequency used in the experiment.

To show the potential of the all-photonic links, we plot in Fig. 2 the transmission distance and achieved net rates for a selection of state-of-the-art all-electronic, photonic-electronic, and all-photonic link demonstrations. Further details to each demonstration are summarized in Table 1. This work shows that high-data rate and long-distance sub-THz links are indeed achievable by using photonic and plasmonic components. Raising the carrier frequency above 100 GHz offers higher bandwidth but also introduces challenges such as atmospheric attenuation, free-space path loss, turbulence, and adverse weather effects. These challenges, along with a comparison to free-space optical (FSO) links, are discussed in Supplementary Note 1, highlighting the complementary nature of the two technologies. To demonstrate the feasibility of a long-distance sub-THz link, we carried out a 1400 m field trial at Dübendorf Air Base, achieving the highest net-rate-distance product reported to date for links above 100 GHz, enabled by high-speed plasmonic modulators and a dual-sideband receiver.

### Setup of the kilometer-range Sub-THz link

**Fig. 3:**
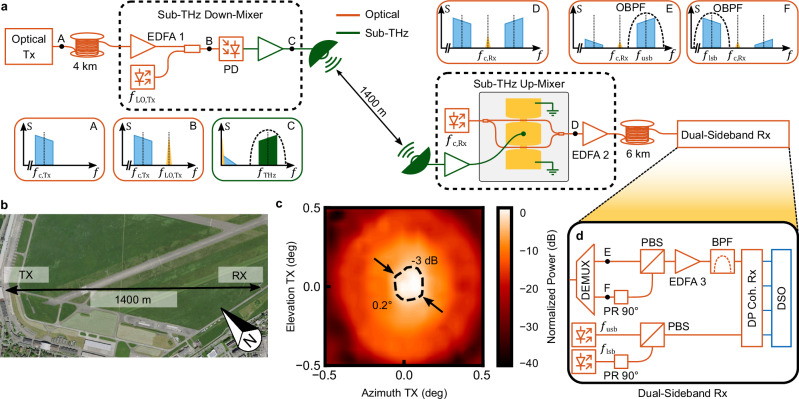
Experimental setup of the all-photonic sub-THz link at the airbase Dübendorf. **a** A coherent optical signal is generated in the optical transmitter (Tx) with a carrier frequency ${f}_{{{\mathrm{c}}},{{\mathrm{Tx}}}}$ and propagates through 4 km of standard-single mode fiber (SSMF). At the sub-THz transmitter, the optical signal is amplified by a an erbium doped fiber amplifier (EDFA 1) and combined with a local oscillator laser with frequency ${f}_{{{\mathrm{LO}}},{{\mathrm{Tx}}}}$. A sub-THz signal is generated through heterodyne mixing in a uni-travelling carrier photodiode (UTC-PD). The sub-THz signal is amplified and fed through a high-gain THz antenna. After propagation through 1400 m of free space, the sub-THz signal is captured by the receiver antenna and electrically amplified, driving a plasmonic Mach-Zehnder modulator (MZM), which up-mixes the sub-THz signal back to the optical domain. A low-noise EDFA 2 is used to amplify the optical signal before propagation through 6 km of SSMF to the dual-sideband coherent optical receiver, where both sidebands are selected using optical band-pass filters (OBPF) and used to detect the signal. **b** Arial view of the airbase Dübendorf in Switzerland, where the experiment has been conducted, taken from Federal Office for Topography swisstopo. **c** Measurement of the half-power beam width of the antenna, which was measured to be 0.2 degree. **d** Implementation of the dual-sideband receiver which shifts one sideband to the orthogonal polarization and uses a commercial dual-polarization coherent receiver (DP Coh. Rx) and a digital sampling oscilloscope (DSO) to receive and sample the signals. The dual-sideband Rx consists of a demultiplexer (DEMUX) separating the sidebands, polarization rotators (PRs) rotating the polarization by 90°, a polarization beam splitter (PBS) for recombination, and a band-pass filter (BPF) to filter the sidebands.

Figure 3a shows the experimental setup, which comprises a photonic sub-THz transmitter and a photonic based sub-THz receiver, capable of directly converting the sub-THz signal into the optical domain. The direct conversion is enabled by the high bandwidth of the plasmonic modulator.

The optical transmitter (Optical Tx) utilizes a laser with a carrier frequency ${f}_{{{{\mathrm{c}}}},{{{\mathrm{Tx}}}}}$ in the C-band at 1551.8 nm. A Nyquist frequency division multiplexed (NFDM)^37^ and power-loaded data signal is encoded onto this laser via a commercial IQ modulator with a 3 dB bandwidth of 38 GHz. The electrical signal is generated using an arbitrary waveform generator (AWG). Emulating the connection from the remote radio unit (RRU), the optical signal (point A) is transmitted through 4 km of standard single-mode fiber (SSMF).

The sub-THz down-mixer has a fiber-optic interface, where the optical signal from the transmitter is first amplified by an erbium doped fiber amplifier (EDFA) and subsequently combined with a free-running laser detuned by the wireless carrier frequency of ${f}_{{{\mathrm{THz}}}}=$ 226 GHz. The combined optical signal (point B) is sent into a UTC-PD, where a sub-THz signal at 226 GHz is generated by heterodyne mixing of the two lasers. The optical signal and detuned laser are set to equal power and are fed with a combined power of 14 dBm into the UTC-PD. The sub-THz signal is amplified by a commercial sub-THz amplifier with a gain of 24 dB, yielding a typical launch power of 12 dBm (point C). The saturated output power and the 1 dB compression point (P1dB) of the Sub-THz amplifiers employed in the down- and up-mixer are frequency dependent, as shown in Supplementary Fig. 1. The carrier frequency of 226 GHz has been chosen to maximize the system’s throughput and was limited by the combined bandwidth of the UTC-PD and the sub-THz amplifier.

The sub-THz signal is transmitted over a 1400 m free-space distance, 2 m above a predominantly meadow terrain with an asphalt runway in between, to the receiver. The channel loss comprises 142.5 dB of FSPL and, based on measured conditions (12.5 °C, 86% relative humidity), 5 dB of atmospheric attenuation, which is partially compensated by two identical antennas having a combined measured gain of 111.7 dB. Each antenna is mounted on a motorized antenna positioner for alignment. The setups are housed inside an office container positioned in front of an open window, offering partial protection from wind exposure. Figure 3c shows the measurement of the radiation pattern, which was obtained by moving the transmitter antenna in azimuth and elevation in a spiral shape and recording the received power by an electrical spectrum analyzer and extender box. A half-power beam width (HPBW) of 0.2° was measured. Therefore, the half-power beam size at the receiver is expected to have a diameter of 4.9 m.

The signal after the receiving antenna is amplified by a second sub-THz amplifier having a gain of 24 dB which directly drives an organic-plasmonic imbalanced Mach-Zehnder modulator^29^ (MZM) via a ground-signal-ground (GSG) RF probe with a characteristic impedance of 50 $\Omega$. The total sub-THz power at the plasmonic MZM was −5.6 dBm. The electrodes of the MZM are modeled as a capacitive load, with an estimated capacitance of 5 fF. Operating well below the cut-off frequency, the MZM presents a high impedance and behaves effectively as an open circuit, resulting in the reflection of the incident RF power back toward the probe and amplifier. Under these conditions, the voltage amplitude at the modulator input is 0.3 V. The plasmonic MZM, which is biased to the null point through wavelength detuning, is used to modulate the amplitude of an optical carrier at 1557.33 nm. The MZM offers a ${V}_{{{{\rm{\pi }}}}}$ of 12.3 V and a fiber-to-fiber insertion loss of 17.5 dB. At the output of the MZM, two sidebands emerge, carrying the identical information as the driving sub-THz signal (point D). The optical signal is amplified by a low-noise EDFA 2 with a gain of 35 dB and a noise figure of 3.4 dB, transmitted through 6 km of SSMF before it is received in the dual-sideband optical coherent receiver^33^, emulating the connection to the baseband unit (BBU). The dual-sideband receiver incorporates a wavelength demultiplexer to separate the two sidebands (point E and F). The polarization of the lower sideband (LSB) is rotated by 90° and is combined again with the upper sideband (USB) via a polarization beam splitter. The signal is amplified by a high-gain EDFA 3, bandpass filtered, and processed by a dual-polarization heterodyne coherent receiver (DP Coh. Rx). EDFA 3 has a noise figure of 4.5 dB and is operated in power-controlled mode, which keeps the input power into the coherent receiver stable. Both sidebands are converted to baseband, where the electrical baseband signal is sampled by a high-speed digital sampling oscilloscope (DSO). Digital signal processing subsequently combines these basebands. The dual-sideband receiver makes use of the fact that both sidebands carry the same information, and the noise picked up after point D to each sideband is uncorrelated. By exploiting the information redundancy, the impact of the noise can be mitigated, resulting in an SNR gain.

### Data Experiment

The transmitted signal was multiplexed into 8 NFDM tributaries, each with a bandwidth of 8 GHz, culminating in a total bandwidth of 64 GHz and an aggregated line rate of 184 Gbit s^−1^. More precisely, the tributary 1-8 were bit-loaded with QAM of order 8, 16, 16, 16, 4, 4, 4 and 4. Additionally, tributary 1-8 were power-loaded with 1.4, −0.1, −2.9, 0.6, −2.4, −0.3, −0.8, and 2.7 dB to compensate for the frequency response of the RF components. The SNRs of the optical back-to-back (B2B) measurements of the respective NFDM tributary were 17.2, 19.7, 21.3, 23.1, 21.9, 20.9, 18.6, and 16.2 dB. Averaging over all 8 tributaries, the highest achievable general mutual information (GMI) is 2.875 bit per symbol, aggregating to a combined bit-error ratio (BER) of 3.83·10^−^^2^.

After recording the two baseband signals at the dual-sideband receiver, the signals were further processed in offline digital signal processing (DSP). The DSP incorporates a blind constant modulus algorithm (CMA) and a blind radius-directed equalizer (RDE), followed by carrier recovery, blind phase search (BPS) and a T/2-spaced feedforward equalizer (FFE). The number of filter taps of the CMA, RDE, and FFE was set to 41, 41, and 223, respectively. The number of taps was not optimized and could potentially be reduced without loss of performance. Combining the two baseband signals was achieved by utilizing a butterfly extension of the FFE. Despite the expected non-linear distortion of the two sub-THz amplifiers in the link, non-linear equalization was omitted, reducing the computational complexity of the DSP. Furthermore, phase noise introduced by the free-running lasers could be corrected by the BPS, without any significant penalty.

**Fig. 4:**
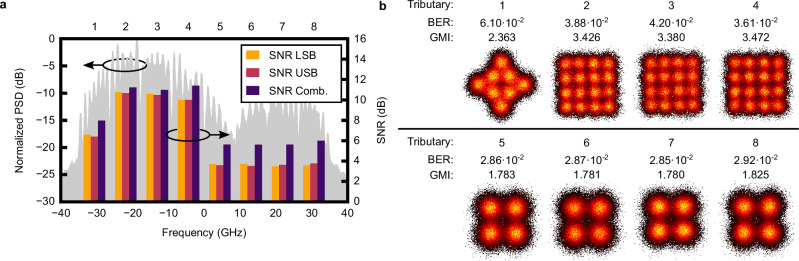
Signal spectra and performance of the dual-sideband receiver. **a** Normalized power spectral density (PSD) of the received electrical baseband signal (grey) and signal-to-noise ratio (SNR) of the received signal of the lower sideband (LSB) and upper sideband (USB) of each tributary (yellow and red) and their combination (purple) in best case conditions where scintillation was at a minimum. The combined SNR of the tributaries with lower baseband power could be improved by the dual-sideband receiver scheme. **b** Constellation diagram of each tributary including bit error rate (BER) and generalized mutual information (GMI). Better performing channels use higher order modulation formats to boost the total capacity.

The resulting baseband power spectral density is shown in Fig. 4a (grey color), along with the SNRs for each tributary. The calculation of the SNR is described in Supplementary Note 3. The SNRs of the lower sideband (LSB) and upper sideband (USB) are also presented, alongside the SNR of the combined signal from the dual-sideband receiver. Tributaries 5-8 have diminished powers, attributable to the reduced output power of the UTC-PD and sub-THz amplifiers at these frequencies, see Supplementary Note 5. By contrast, tributaries 1–4 operate in a frequency range where the sub-THz amplifier exhibits a higher saturated output power and a higher 1 dB compression point (P1dB), see Supplementary Fig. 6. This operating regime helps to suppress nonlinear distortion and supports the use of higher-order modulation formats. The dual-sideband reception was particularly efficient in improving these tributaries with a low SNR. The SNR of tributaries 5-8 benefited by up to 2 dB, indicating that the dominant noise source is the amplified spontaneous emission noise introduced by the low-noise EDFA 2 after point D, see Fig. 3a. On the other hand, the dual-sideband receiver’s SNR improvement of tributaries 1–4 was below 2 dB, indicating that the dominant noise in both sidebands is correlated and originates prior to the plasmonic modulator. This finding agrees well with the theoretical link budget, as discussed in Supplementary Note 4.

Figure 4b presents the constellation diagrams, the bit-error rate (BER), and general mutual information (GMI) of each tributary. These measurements are performed in best case conditions, where scintillation was at a minimum. Since forward error correction (FEC) is applied prior to bit demultiplexing, the overall GMI is obtained by averaging the values across all tributaries. This results in an average GMI of 2.48 bit per symbol. Multiplying this value by the 64-GBaud symbol rate yields an achievable information rate (AIR) of 158.7 Gbit s^−1^. To determine the appropriate FEC scheme, we evaluate the normalized GMI (NGMI), which is a more reliable predictor of post-FEC performance compared to the pre-FEC BER^38^. Normalizing the total GMI by the average bits per symbol gives an NGMI of 0.8613. Using a concatenated SD + HD FEC with a 0.8574 NGMI threshold and 20 % overhead^39^ results in a net information rate of 153 Gbit s^−1^. For comparison, we also evaluate the system performance when only the lower sideband (LSB) is detected. In this single-sideband configuration, the average GMI is 2.25 bit per symbol, corresponding to an AIR of 144.0 Gbit s^−1^. Including the upper sideband (USB) in a dual-sideband reception scheme increases the GMI to 2.48 bit per symbol and the AIR to 158.7 Gbit s^−1^. This represents an improvement of approximately 10 percent. The gain requires an additional coherent receiver and wavelength-demultiplexing stage, but it provides a substantial increase in spectral efficiency.

Table 1 summarizes a selection of state-of-the-art link demonstrations sorted by the product of distance and net rate per polarization and channel. For sub-THz links with frequencies above 100 GHz, this work shows, to the best of our knowledge, the highest combination of distance and net rate.

### Influence of atmospheric turbulence on Sub-THz links

Beyond the data transmission over large distances, we further investigate the above-discussed challenge of atmospheric turbulence. In comparison to many laboratory-based works, here we measure the sub-THz link outside of laboratory conditions in a field experiment.

**Fig. 5:**
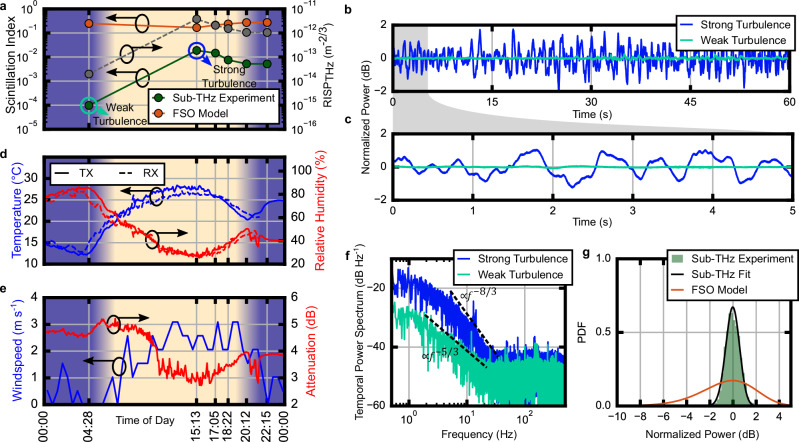
Impact of atmospheric turbulence on sub-THz compared with free-space optic (FSO) links. **a** Scintillation index (SI) and refractive index structure parameter (RISP) of the sub-THz link measured over the course of the day taken on 2^nd^ June 2023 compared with calculated SIs of the equivalent FSO link for the same conditions. The time stamps indicate the times when scintillation measurements have been taken. **b** Recorded time traces of the normalized power for worst-case and best-case conditions, (**c**) time traces zoomed in to the first 5 seconds. **d** Measured temperature and humidity values at the transmitter (solid line) and receiver (dashed line). **e** Wind speed measurement at the airbase Dubendorf^50^ and estimated atmospheric attenuation according to ITU-R P.676^51^. **f** Fourier transform of the measured normalized power bandpass filtered between 0.5 Hz and 500 kHz, and **g** Measured histogram of the normalized received power of the 1.4 km sub-THz link in worst-case turbulence conditions and fitted $\Gamma \Gamma$-model to extract a RISP of 3.92·10^−12^ m^−2/3^ (solid green line). The extracted RISP was used to calculate the expected probability density function (PDF) of a hypothetical 1.4 km FSO link operating at 1550 nm, showing much higher power fluctuations (solid orange line).

To quantify the effect of atmospheric turbulence on the sub-THz link, the optical power of a single sideband at the receiver at point D was recorded for 60 s at 6 different time instances during the day using a power meter with an averaging time of 200 µs. The relative sub-THz power fluctuations were derived from the optical sideband measurements with an estimated accuracy of ±0.05 dB. To confirm that the origin of the power fluctuations stem from the free-space channel, short range indoor experiments in the laboratory over 5 m have been conducted in prior studies^32^, showing that the received power is stable. Therefore, the power fluctuations in this study are attributed to atmospheric turbulence and potentially wind-induced pointing error effects. We use a $\Gamma \Gamma$-distribution and Andrew’s method^40^ to extract the scintillation index (SI) ${\sigma }_{{{\mathrm{I}}}}^{2}$ and the refractive index structure parameter (RISP) from the measurements. The extracted SI (orange) and RISP (grey) are provided in Fig. 5a. Details on the theory and the calculations are given in Supplementary Notes 1 and 2. The full and zoomed-in time traces of the measured normalized power in the weakest (light blue) and strongest (dark blue) turbulence conditions are shown in Figs. 5b, c, respectively. One can see that for the recorded time, the SI varied between 1·10^–4^ during weak and 0.019 during strong turbulence conditions. To filter out non-turbulence related low-frequency variations due to the system’s drift^41^ and high frequency noise of the power meter^42^, a second-order butter-worth bandpass filter was applied to the recorded time traces with an upper- and lower cut-off frequency of 0.5 Hz and 500 Hz, respectively. The filtered temporal power spectra of power scintillation are shown in Fig. 5f. One can see that the temporal power spectrum of scintillation decreases with frequency with a power law according to $\propto {f}^{-8/3}$ and $\propto {f}^{-5/3}$ and plateaus beyond 30 Hz, which is in good agreement with the Kolmogorov power-law spectrum^43^. However, it should be noted that the SI and therefore RISP in this study is likely overestimated, as the observed power fluctuations are possibly driven by a combination of atmospheric turbulence and wind-induced pointing errors. Although the antennas were located indoors, wind effects cannot be entirely excluded. Figure 5d shows the measured temperature and relative humidity at the transmitter and receiver, while Fig. 5e presents the calculated atmospheric attenuation and measured average wind speed at the airport. Scintillation levels are highest in the afternoon, coinciding with peak temperature, but also increase when wind speeds are strongest, as shown in Fig. 5e. This suggests that scintillation is the dominant factor, though some contribution from wind effects remains possible.

Despite strong turbulence conditions with RISP values as high as 3.92·10^−12^ m^−2/3^ the sub-THz link fading loss (see Supplementary Note 2) is below 2.4 dB. This means that one does not require further mitigation techniques and can be taken care of by the dynamic range of the optical receiver.

To showcase the above-discussed resilience of sub-THz compared to FSO frequencies, we used the extracted RISP, i.e., grey curve in Fig. 5a, to calculate the SI of an equivalent FSO link model with an aperture radius of 2.5 cm. The SI of the equivalent FSO link is shown as an orange curve in Fig. 5a. By comparing the SI of FSO and sub-THz, one can see that the scintillation index of FSO in weak turbulence is still one order of magnitude higher compared to sub-THz link in strong turbulence. Interestingly, the SI of the theoretical FSO link is not proportional to the RISP values, indicating that the saturation regime is reached, which can also be observed in Supplementary Fig. 2a. Figure 5g shows the probabilistic density function of the measured power fluctuations in sub-THz link in the strong turbulence condition, alongside the fitted $\Gamma \Gamma$-distribution. The expected power distribution of the FSO model in similar conditions with the same RISP indicates much stronger power fluctuations and therefore higher fading loss of 13 dB. To achieve reliable communication, the link budget needs to be adjusted to compensate for these fluctuations, as discussed in Supplementary Fig. 2b. This fading can be improved by use of adaptive optics, which add to the system complexity and cost.

## Discussion

In this work, we present a sub-THz communication link operating at a carrier frequency of 226 GHz, achieving, to the best of our knowledge, the highest net-rate-distance product of 242.2 Gbit s^−1^ km for carrier frequencies above 100 GHz. We compared sub-THz technology with free-space optical (FSO) systems, concluding that sub-THz offers high-capacity, low-complexity connectivity with enhanced robustness against adverse weather. The main challenges to overcome and allow for long-term deployment are the precise and periodic realignment of the antennas with 0.1 degree precision and RF-packaging of the plasmonic modulator. In this work, the plasmonic modulator was probed using a sensitive RF probe, rendering the experimental setup susceptible to vibrations and environmental exposure. These limitations could be mitigated in future implementations through device packaging. For other sub-THz components, low-loss coupling from a rectangular waveguide to an on-chip transmission line has been demonstrated using an on-chip dipole antenna positioned at a quarter-wavelength from the waveguide termination. This technique is established in sub-THz amplifier packaging^44,45^ and insertion losses of 1 dB up to 380 GHz are achievable^46^. Plasmonic modulators have been directly integrated with on-chip antennas, enabling efficient capture of the incident RF field and direct electrical driving of the modulator^30,47,48^. Taking together, these established approaches indicate a pathway toward packaged plasmonic modulators with similarly low insertion loss, although such an implementation has not yet been experimentally demonstrated. Within this broader context, our transparent, all-photonic link, enabled by plasmonic modulators with bandwidths exceeding 1 THz, allows seamless integration into fiber optic networks through direct sub-THz to optical conversion. Additionally, we report outdoor measurements of atmospheric turbulence effects on a THz link, showing low scintillation index of 0.019 even under strong turbulence. The promising performance observed in this study highlights the need for future work involving long-term sub-THz link deployment and assessment of BER and EVM across diverse weather conditions. All-photonic sub-THz links offer high data rates in the kilometer range, making them a viable solution for backhauling in future radio access networks.

## Supplementary information

Supplementary Information

Transparent Peer Review file

## Data Availability

Data from this work is provided in the manuscript, supplementary information figures and from the corresponding author upon request.

## Code availability

The open-source software LabExT^49^ was used to control the laboratory instruments, perform the measurements, and collect the data. LabExT is publicly available on GitHub. Code from published literature is used for offline digital signal processing (DSP) of the received data. The implemented algorithms are well established, and all relevant processing parameters are provided in the main text. The DSP code is available from the corresponding author upon request.
