# Supplementary material for: Plasmonic modulator enabling kilometer-range high-throughput sub-THz links for radio access networks
Source: Nat Commun. 2026 Apr 18;17:5384. doi: 10.1038/s41467-026-72053-z (PMC13275729; doi:10.1038/s41467-026-72053-z)
Supplement: Supplementary file 1 — Supplementary Information [file 41467_2026_72053_MOESM1_ESM.pdf]

Supplementary Materials for

Plasmonic Modulator Enabling Kilometer-Range High-Throughput Sub-THz Links for Radio Access Networks

Boris Vukovic[1]*, Laurenz Kulmer[1], Tobias Blatter[1], Yannik Horst[1], Marcel Destraz[2], Wolfgang Heni[2], Stefan M. Koepfli[1], Hande Ibili[1], Michael Baumann[1], Yuriy Fedoryshyn[1], Jasmin Smajic[1], Sarperi Luciano[3], and Juerg Leuthold[1]*

[1]ETH Zurich, Institute of Electromagnetic Fields (IEF), 8092 Zurich, Switzerland
[2]Polariton Technologies, 8134 Adliswil, Switzerland
[3]ZHAW, Institute of Signal Processing and Wireless Communications (ISC), 8401 Winterthur, Switzerland

* *corresponding email:* boris.vukovic@ief.ee.ethz.ch, juerg.leuthold@ief.ee.ethz.ch

The PDF file includes:

Supplementary Notes 1 – 5
Supplementary Figures 1 – 6
Supplementary Tables 1 – 4
Supplementary References 1 – 18

Supplementary Note 1: Comparison of Sub-THz and FSO Communications

Moving from current microwave carrier frequencies to the sub-THz introduces increased atmospheric attenuation, even in clear weather conditions. This attenuation is primarily influenced by the absolute water content in the atmosphere (measured in g m^{-3}) and absorption lines from oxygen at 60 GHz, as illustrated in Supplementary Figure 1a. For communication links over distances in the kilometer range or greater, two frequency bands are of particular interest, namely 100–170 GHz and 200–300 GHz. Secondly, another challenge is the rise in free-space path loss (FSPL), which is expressed as $L_{\mathrm{fspl}} = \left(\frac{4\pi d f}{\mathrm{c}}\right)^2$, where the FSPL increases quadratically with both the frequency f and the distance d[1]. Supplementary Figure 1b presents the total channel loss for various link distances, incorporating both FSPL and atmospheric absorption.

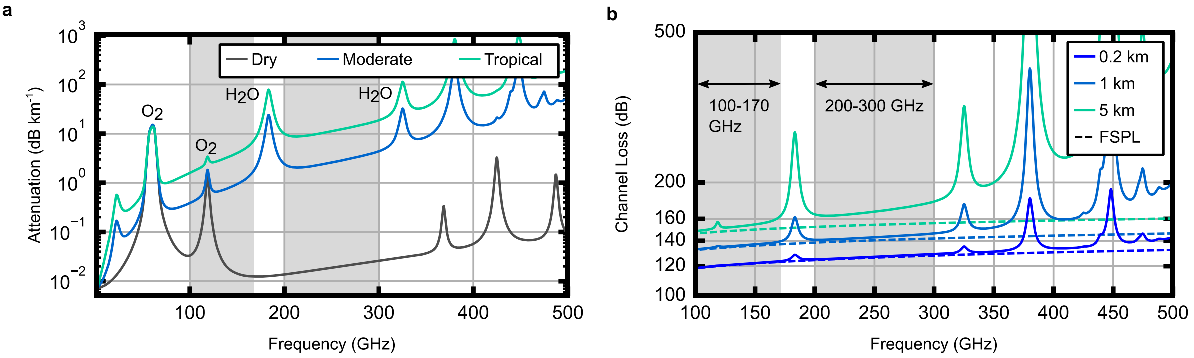

Supplementary Fig. 1: Channel losses of sub-THz waves due to molecular attenuation and free-space path loss (FSPL) according to ITU-R P.676[2]. a Attenuation of sub-THz frequencies mainly comes from molecular absorption of water molecules in the air. Calculations are done for moderate climate (*T* = 15 °C, *RH* = 50 %, *ρ* = 6.4 g m^{-3}) and tropical climate (*T* = 30 °C, *RH* = 80 %, *ρ* = 24.3 g m^{-3}). **b** The total channel loss is comprised of attenuation (moderate climate) and FSPL. The frequency windows between 100 – 170 GHz and 200 – 300 GHz are of particular interest for kilometer range communication.

The increased FSPL must be compensated for by employing high-gain antennas, which feature very directive, beam-like radiation patterns. The link loss, excluding molecular absorption, can be expressed as

$$L_{\mathrm{link}} = \frac{1}{D_{0,\mathrm{tx}} D_{0,\mathrm{rx}}} \left(\frac{4\pi d f}{\mathrm{c}}\right)^2 = \frac{\mathrm{c}^2 d^2}{A_{\mathrm{eff,tx}} A_{\mathrm{eff,rx}} f^2}, \qquad \text{(S1)}$$

where $D_{0,\mathrm{tx}}$ and $D_{0,\mathrm{rx}}$ represent the directivities of the transmitter and receiver antennas, which can be rewritten in terms of the effective area of the antennas $A_{\mathrm{eff,tx}}$ and $A_{\mathrm{eff,rx}}$, as the effective area and directivity are related by $A_{\mathrm{eff}} = \frac{\mathrm{c}^2}{4\pi f^2} D_0$. As evident from (S1), increasing carrier frequency f while keeping the effective area A_{eff} and therefore geometrical size of the antenna constant, reduces the link loss. This loss reduction comes from the fact that the beam becomes more directive, and more power is collected by the receiver antenna. Antenna gains of up to 60 dBi are achievable, allowing for compensation of 120 dB of FSPL, though this requires an alignment accuracy within 0.1 degrees. FSO links are an extreme example, where the aperture of the terminals, e.g. effective areas, are much bigger compared to the wavelength. Therefore, FSO links have very highly directive beams which also requires even higher accuracy below 0.01° (200 µrad) range[3].

With the potential to compensate for the FSPL, we next discuss the influence of adverse weather conditions. To highlight the resilience under different weather conditions and to facilitate the selection between FSO and sub-THz links for each scenario, we present a comparative analysis of signal attenuation across various weather conditions in Table 1. FSO exhibits low attenuation in clear weather and strong resilience to heavy rain; however, under foggy conditions where visibility drops below 50 meters, attenuation can reach up to 272 dB per km, rendering communication impossible. In contrast, sub-THz links are primarily attenuated by rain, as the raindrop size is comparable to the wavelength, leading to high losses due to Mie scattering. In heavy rain conditions, with rainfall rates of 20 mm per hour, attenuation levels can reach 25 dB per km, a factor that must be accounted for in link budget calculations. During snowfall, sub-THz links experience lower absorption compared to FSO, providing a more reliable communication channel under such conditions. Each weather scenario can therefore be covered by these two technologies.

Supplementary Tab. 1: Comparison of weather induced attenuation for free-space optics at a wavelength of 1550 nm and sub-THz link at frequencies between 200-300 GHz.

	Clear[4,5,2] (Visibility: >20 km)[a]	Haze and Pollution[4,5,6] (Visibility: 4 – 2 km)	Fog and Clouds[5,7,8,9] (Visibility: 0.5 – 0.05 km)	Rain[10,11] (Rainfall rate: 20 mm per hour)	Snow[4, 12] (Snowfall rate: 4 mm per hour)
FSO (dB km^{-1}) 1550 nm	0.2	2 - 4	21 – 272	10	Wet snow: 11<br>Dry snow: 38
Sub-THz (dB km^{-1}) 200 – 300 GHz	2 – 5	0.1	0.75 – 7.5	25	Wet snow: 15<br>Dry snow: 10

[a]Weather induced losses are added to the clear weather attenuation.

In addition to weather-related attenuation, the resilience of sub-THz links to atmospheric turbulence is another crucial factor. Measurements conducted in turbulence chambers show that sub-THz links experience significantly less power fading due to atmospheric turbulence compared to FSO systems[13]. Power fading can disrupt communication, and in FSO systems, compensation often requires adaptive optics, increasing both system complexity and cost[4]. While higher optical launch powers can be employed to improve FSO performance in earth-to-satellite communication, ground-based FSO links are constrained by eye-safety regulations designed to protect both humans and wildlife.

Supplementary Figure 2a illustrates the scintillation index based on Andrew's method[14] under weak, moderate, and strong turbulence conditions. Notably, sub-THz links exhibit a reduction in turbulence-induced effects by more than two orders of magnitude compared to FSO links. This difference arises from the fact that the wavelength of sub-THz waves is roughly three orders of magnitude larger than that of FSO waves. Scintillation is caused by refractive-index inhomogeneities in the atmosphere, which act as random phase-distorting elements. To accumulate the same level of phase distortion, a sub-THz beam would need to propagate over distances that are orders of magnitude longer than those required for an FSO beam[15]. Consequently, sub-THz links are significantly less susceptible to atmospheric turbulence. The strength of the turbulence is quantified by the refractive index structure parameter (RISP), while the resulting power fluctuations at the receiver are quantified by the scintillation index σ_I^2, which is calculated according to

$$\sigma_I^2 = \frac{\langle P^2 \rangle}{\langle P \rangle^2} - 1, \qquad \text{(S2)}$$

where P represents the measured power at the receiver and $\langle\cdot\rangle$ denotes the time-averaged value. To operate the wireless link reliably even when deep power fades occur, an additional power margin needs to be considered in the link budget. Supplementary Figure 2b shows the power fading loss that one needs to consider in order to operate the link at a 99.99 % reliability. This loss must be compensated in the link budget, negatively impacting transmission distance and capacity. Fading loss at sub-THz frequencies is in the order of 1 dB even in strong turbulence conditions, making them resilient and reliable. FSO links on the other hand can have fading losses exceeding 20 dB, which needs to be compensated by either higher launch powers or adaptive optics. Supplementary Figures 2c and 2d indicate that, under the aforementioned conditions, the scintillation index and fading losses fall within the green-shaded region, indicating a favorable regime for sub-THz relative to FSO links. More information on the calculations can be found in the Supplementary Note 2.

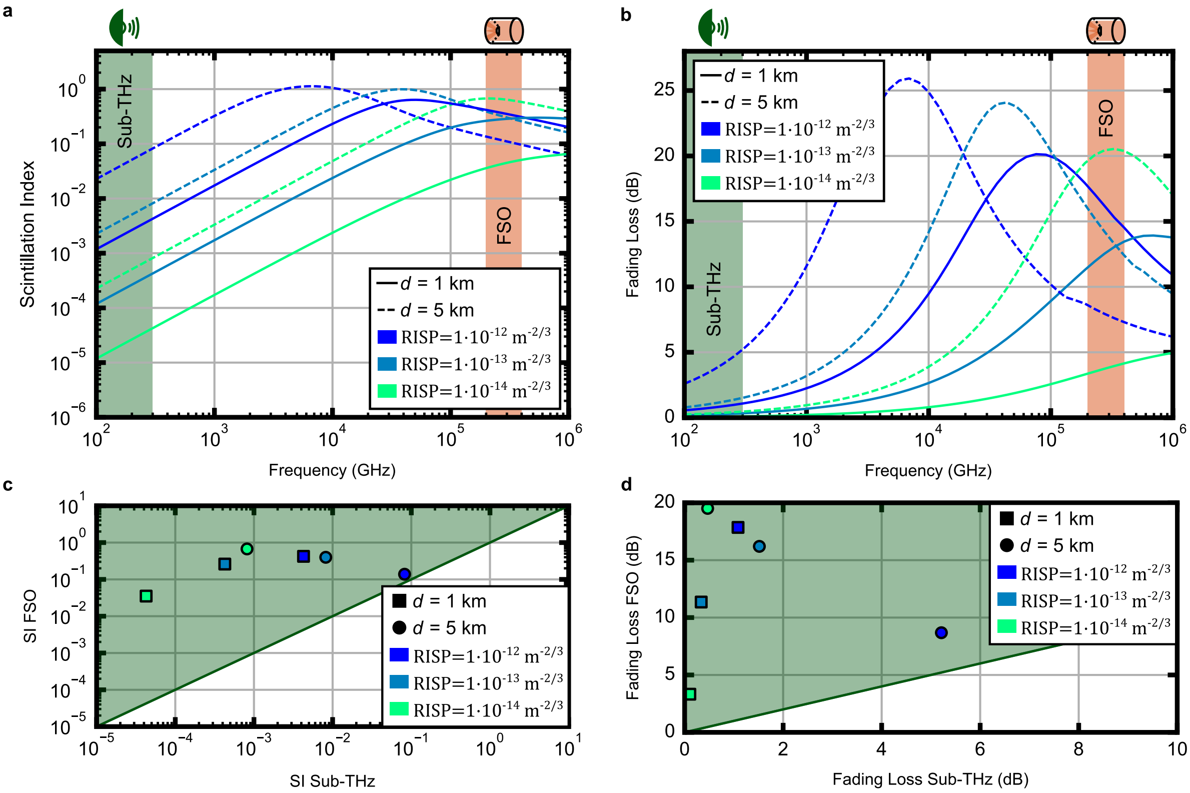

Supplementary Fig. 2: Comparison of scintillation index (SI) and fading loss according to Andrew's method between sub-THz and free-space optics (FSO) in weak, moderate, and strong turbulence conditions at link distances of 1 km and 5 km assuming a receiver aperture radius of 2.5 cm. a Scintillation index for three turbulence conditions, where the turbulence strengths relate to the refractive index structure parameter values (RISP). **b** Additional fading loss that needs to be compensated to have a 99.99 % reliability of the wireless link. **(c, d)** Comparison of SI and fading loss between FSO (1550 nm) and Sub-THz (300 GHz) in above mentioned conditions. The green shaded area is where sub-THz is advantageous compared to FSO.

Supplementary Note 2: Modeling of Turbulence-Induced Impairments in Sub-THz Wireless Links

Atmospheric turbulence is air movement caused by wind or ground heating from the sun, creating turbulent eddies that break into smaller ones until their energy dissipates as heat. Local temperature, pressure and humidity differences lead to refractive index variations, which have different effects on a beam of electromagnetic radiation propagating through the turbulent air, namely beam wander, beam scintillation and beam spreading[16]. The relation between the state of the atmosphere and the resulting power fluctuations at the receiver can be modeled

by a ΓΓ-distribution, which is suitable for weak and strong turbulence regimes[16]. The probability density function (PDF) of the ΓΓ-distribution is given by

$$f_{\Gamma\Gamma}(I) = \frac{2(\alpha\beta)^{\frac{\alpha+\beta}{2}}}{\Gamma(\alpha)\Gamma(\beta)} I^{\left(\frac{\alpha+\beta}{2}-1\right)} K_{\alpha-\beta}\left(2\sqrt{\alpha\beta I}\right), \quad \text{(S3)}$$

where $I = \langle P\rangle^2/\langle P^2\rangle$ is the normalized power collected by the receiver aperture, α and β the effective number of small- and large-scale eddies, $K_v(\cdot)$ is the modified Bessel function of second kind of order v and $\langle\cdot\rangle$ the time average[4]. By fitting the ΓΓ-distribution described in (S1) to the measured normalized power distributions (see Supplementary Figure 3), α_{THz} and β_{THz} can be extracted for the sub-THz link. From α and β, the refractive index structure parameter (RISP) C_{n}^2 can be calculated according to (S2), which indicates the strength of the atmospheric turbulence.

$$\alpha = \left(\exp\left(\frac{0.49\chi^2}{\left(1 + 0.18\delta^2 + 0.56\chi^{\frac{12}{5}}\right)^{\frac{7}{6}}}\right) - 1\right)^{-1}$$

$$\beta = \left(\exp\left(\frac{0.51\chi^2\left(1 + 0.69\delta^2\chi^{\frac{12}{5}}\right)^{-\frac{5}{6}}}{\left(1 + 0.9\delta^2 + 0.62\chi^{\frac{12}{5}}\right)^{\frac{7}{6}}}\right) - 1\right)^{-1} \quad \text{(S4)}$$

The parameters $\chi^2 = 0.5C_{\text{n}}^2 k^{7/6} d^{11/6}$ and $\delta = \left(\frac{kr_{\text{rx}}^2}{d}\right)^{1/2}$ are given by the wavevector k of air, the link distance $d = 1400$ m and the receiver aperture radius $r_{\text{rx}} = 26$ cm. The normalized power fluctuations at the receiver are captured by the scintillation index (SI) σ_{I}^2, which can be calculated from

$$\sigma_I^2 = \frac{1}{\alpha} + \frac{1}{\beta} + \frac{1}{\alpha\beta}. \quad \text{(S5)}$$

The fits and histogram of the measured data are shown in Supplementary Figure 3 and the fitted parameters are listed in Supplementary Table 3.

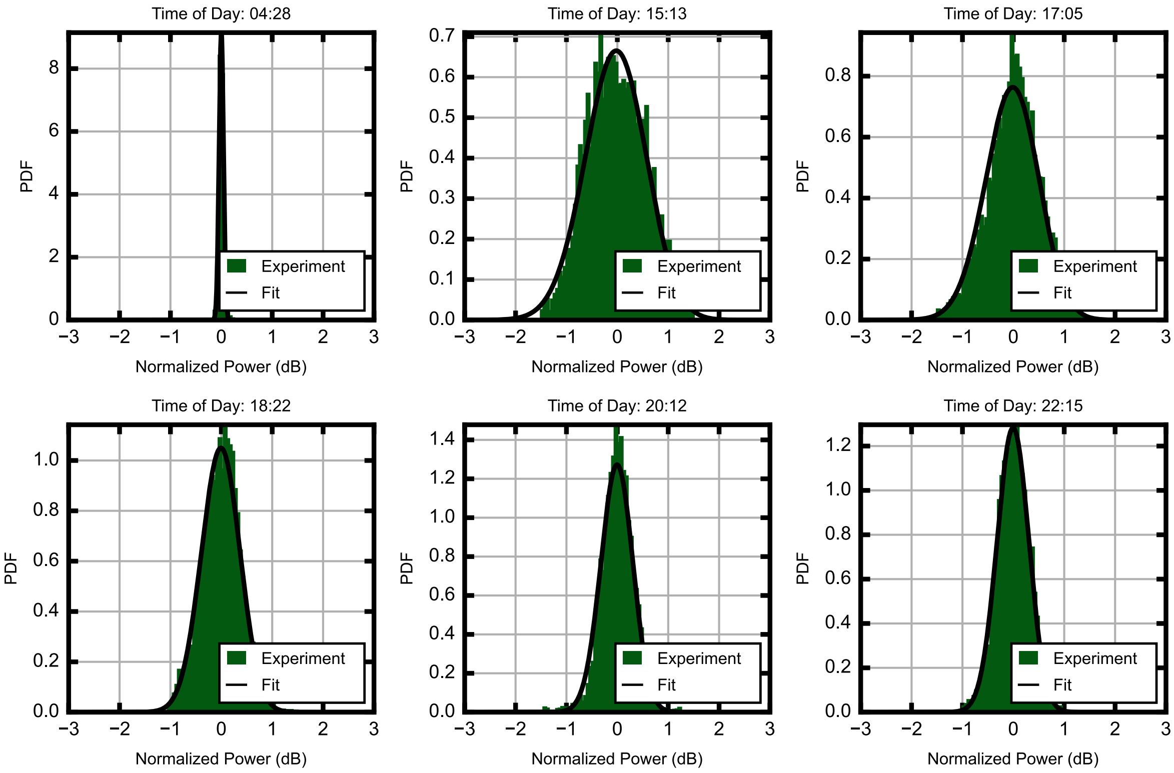

Supplementary Fig. 3: Histogram of normalized received power. The green bars indicate the histogram of the recorded time traces at different time instances which is fitted with by the ΓΓ-distribution (black solid line). Lowest spread is measured at 04:28 and highest spread at 15:13.

Supplementary Tab. 2: Extracted parameters from the fit and calculated scintillation index and RISP for the sub-THz link.

Time of Day	α_{THz}	β_{THz}	$\sigma^2_{\text{I,THz}}$	$C^2_{\text{n,THz}}$ ($\text{m}^{-2/3}$)	$L_{\text{fade,dB}}$ (dB)
04:28	19017.9	20459.6	$1.0\cdot10^{-4}$	$2.04\cdot10^{-14}$	0.16
15:13	103.3	103.6	0.01943	$3.92\cdot10^{-12}$	2.39
17:05	90.0	290.0	0.01460	$2.06\cdot10^{-12}$	2.08
18:22	267.6	264.3	0.00753	$1.52\cdot10^{-12}$	1.45
20:12	384.8	383.5	0.00521	$1.05\cdot10^{-12}$	1.20
22:15	394.4	393.6	0.00508	$1.03\cdot10^{-12}$	1.19

The RISP at free-space optics (FSO) wavelengths can be estimated from the RISP at sub-THz and the temperature-dependence of the refractive index of FSO and sub-THz[15] according to

$$C^2_{\text{n,FSO}} = C^2_{\text{n,THz}} \cdot \frac{\left(\frac{\partial n_{\text{THz}}(T)}{\partial T}\right)^2}{\left(\frac{\partial n_{\text{FSO}}(T)}{\partial T}\right)^2} = C^2_{\text{n,THz}} \cdot \frac{6021.76 \cdot 10^{-12}\left(\frac{P_{\text{a}}}{T^2} + 9620 \cdot \frac{P_{\text{v}}}{T^3}\right)^2}{6241 \cdot 10^{-12}\frac{P_{\text{a}}^2}{T^4}}, \quad \text{(S6)}$$

where $C^2_{\text{n,FSO}}$ and $C^2_{\text{n,THz}}$ are the RISP and n_{FSO} and n_{THz} the refractive index at FSO and sub-THz wavelengths, respectively. P_{a} denotes the atmospheric pressure in millibar, P_{v} the water vapor pressure in millibar and T the temperature in Kelvin. The temperature-dependent refractive indices in (S4) are given by (S5)[13].

$$n_{\text{FSO}} = 1 + 79 \cdot 10^{-6} \frac{P_\text{a}}{T}$$

$$n_{\text{THz}} = 1 + 77.6 \cdot \frac{10^{-6}}{T} \left(P_\text{a} + 4810 \frac{P_\text{v}}{T} \right) \quad \text{(S7)}$$

The local temperature and humidity values have been tracked using a local weather station located at the transmitter. The measured values are shown in Figure 7d. A reduced atmospheric pressure of 1013 mbar was measured in a nearby weather station, corresponding to a pressure of $P_\text{a} \approx 962$ mbar at the measurement location.

Using the RISP for FSO wavelength, the SI $\sigma^2_{\text{I,FSO}}$, α_{FSO}, and β_{FSO} can be calculated according to (S2). The values are listed in Supplementary Table 2. For the hypothetical FSO link we assumed an aperture radius of 2.5 cm, which is a common terminal size for FSO links.

Supplementary Tab. 3: Parameters for the FSO link calculated from the RISP from the sub-THz link.

Time of Day	α_{FSO}	β_{FSO}	$\sigma^2_{\text{I,FSO}}$	$C^2_{\text{n,FSO}}$ ($\text{m}^{-2/3}$)	$L_{\text{fade,dB}}$ (dB)
04:28	5.41	17.16	0.25	$4.11 \cdot 10^{-14}$	10.8
15:13	6.15	881.69	0.16	$6.73 \cdot 10^{-12}$	9.6
17:05	4.75	377.29	0.21	$3.61 \cdot 10^{-12}$	11.2
18:22	4.29	269.82	0.24	$2.81 \cdot 10^{-12}$	11.8
20:12	3.79	177.82	0.27	$2.05 \cdot 10^{-12}$	12.9
22:15	3.74	171.08	0.27	$1.99 \cdot 10^{-12}$	13.1

The fading loss $L_{\text{fade,dB}}$ can be calculated by

$$F_{\Gamma\Gamma}(I) = \int_0^I f_{\Gamma\Gamma}(x) \mathrm{d}x$$

$$L_{\text{fade,dB}} = -10\log_{10} \left(F^{-1}_{\Gamma\Gamma}(p_{\text{fade}} | \alpha, \beta) \right), \quad \text{(S8)}$$

where $F_{\Gamma\Gamma}(I)$ is the cumulative distribution function of the $\Gamma\Gamma$-distribution, $F^{-1}_{\Gamma\Gamma}(\cdot)$ the inverse of $F_{\Gamma\Gamma}(I)$, p_{fade} the probability that the normalized received power I is below the receiver threshold and the communication link is disrupted. For all calculations, $p_{\text{fade}} = 1 - 0.9999$ was set to achieve a 99.99% reliability.

Supplementary Note 3: Calculation of SNR

This section describes how the signal-to-noise ratio (SNR) of each tributary is calculated after digital signal processing (DSP). The SNR of the received tributary signal $Y = N(0, \sigma_\text{s} + \sigma_\text{n})$ is calculated by comparing it to the transmitted signal $X = N(0, \sigma_\text{s})$ and assuming white Gaussian noise (AWGN) is added to Y. The noise power N_0 is defined as

$$N_0 = \text{Var}(Y - hX) = \text{Var}(Y) + h^2\text{Var}(X) - 2h\text{Cov}(X, Y), \quad \text{(S9)}$$

where X is the normalized transmitted signal, Y is the normalized received signal and h a proportionality factor. The noise power N_0 is minimized by choosing $h = \text{Cov}(X, Y)/\text{Var}(X)$. Inserting h into (S7) yields

$$N_0 = \text{Var}(Y) - \frac{\text{Cov}(X, Y)^2}{\text{Var}(X)}. \quad \text{(S10)}$$

Calculating the SNR yields

$$\mathrm{SNR} = \frac{\mathrm{Var}(Y) - N_0}{N_0} = \frac{\mathrm{Var}(Y)}{N_0} - 1 = \frac{1}{1 - \frac{\mathrm{Cov}(X,Y)^2}{\mathrm{Var}(X)\mathrm{Var}(Y)}} - 1. \tag{S11}$$

Supplementary Note 4: Link Budget

Supplementary Table 3 presents the theoretical link budget for each tributary. Signal power spectral density (PSD) is propagated through the channel using both measured and theoretical power spectra of the system components and the free-space link, see Supplementary Figure 2 and Figure 7e. Noise from sub-THz amplifiers is added to the signal and propagated through the channel. The noise figure of the sub-THz amplifier is assumed to be 20 dB. The SNR of the preamplifier coherent receiver is limited by the ASE-LO beat noise and is estimated using

$$\mathrm{SNR} = \frac{P_\mathrm{s}}{hfn_\mathrm{sp}B}, \tag{S12}$$

where P_s is the optical signal power, hf the photon energy, n_sp the SNR degradation from the shot-noise limit and B the bandwidth[17]. For high gain values, the SNR degradation of the amplifier chain is approximately $n_\mathrm{sp} \approx F_\mathrm{tot}/2$, where F_tot is the combined noise factor of the receiver chain, which consist of low-noise EDFA 2, loss of the demultiplexer and polarization beam splitter and preamplifier EDFA 3, see Figure 4d. According to the Friis formula, the total noise figure can be calculated by

$$F_\mathrm{tot} = F_\mathrm{EDFA2} + \frac{L_\mathrm{DEMUX}F_\mathrm{EDFA3} - 1}{G_\mathrm{EDFA2}}, \tag{S13}$$

where F_EDFA2 and G_EDFA2 are the noise factor and gain of the low-noise EDFA 2, L_DEMUX the loss of the demultiplexer and polarization beam splitter and F_EDFA3 the noise factor of the preamplifier EDFA 3. The noise figure of EDFA2 is 3.7 dB. To estimate the SNR after the dual-sideband receiver, the ASE-LO beat noise power was reduced by 3 dB. Supplementary Figure 4 shows the power spectral density of the signal after the plasmonic modulator (see point D in Fig. 3) and the contributing noise sources. The dominant noise sources were the sub-THz amplifier and the low-noise EDFA 2 at the receiver. The dual-sideband receiver's SNR gain matches the measured results well, showing that tributaries with lower optical sideband power are primarily limited by ASE-LO beat noise and benefit the most from the dual-sideband approach.

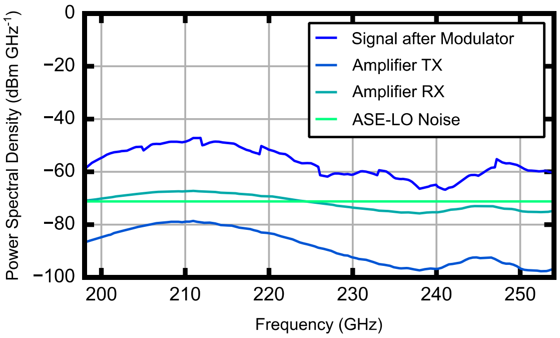

Supplementary Fig. 4: Calculated power spectral density of the signal and different noise sources. The dominant noise sources are identified to be the sub-THz amplifier at the sub-THz receiver (Amplifier RX) and the amplified spontaneous emission-local oscillator beat noise (ASE-LO) beat noise.

Supplementary Tab. 4: Theoretical link budget calculation for each tributary.

	Unit	Tributary							
Tributary Number		1	2	3	4	5	6	7	8
Bandwidth	GHz	8	8	8	8	8	8	8	8
Optical Launch Power (point A)	dBm	3.2	1.6	-1.2	2.3	-0.7	1.4	0.9	4.5
Optical Launch SNR (point A)	dB	17.2	19.7	21.3	23.1	21.9	20.9	18.6	16.2
Sub-THz Launch Power (point C)	dBm	2.5	4.7	4.3	2.7	-0.3	0.0	-0.3	5.1
Sub-THz Launch SNR (point C)	dB	17.1	19.6	21.1	22.8	21.4	20.6	18.4	16.2
Free-Space Path Loss	dB	-141.5	-141.7	-142	-142.3	-142.6	-142.8	-143.1	-143.3
Attenuation	dB	-5	-4.5	-4.5	-4.6	-4.8	-5	-5.3	-5.7
Antenna Gain	dB	111.7	111.7	111.7	111.7	111.7	111.7	111.7	111.7
Excess Loss	dB	7.0	7.0	7.0	7.0	7.0	7.0	7.0	7.0
Sub-THz Received Power	dBm	-39.2	-36.8	-37.5	-39.4	-42.9	-43.1	-44.1	-39.1
Sub-THz Power at Modulator	dBm	-14.1	-9.9	-11.1	-15.0	-21.8	-23.6	-22.9	-19.2
Optical Sideband Power (point D)	dBm	-44.5	-40.3	-41.5	-45.4	-52.1	-54	-53.2	-49.6
SNR Single Sideband (point E/F)	dB	12.3	15.8	15.6	12.7	6.2	4.4	5.0	8.3
SNR Dual-Sideband	dB	13.5	16.8	16.9	14.5	8.3	6.5	7.0	10.0

Supplementary Note 5: Sub-THz Components

Supplementary Figure 5 shows the normalized response and gain spectrum of the UTC-PD and sub-THz amplifiers used in the experiment. The high differences in output power across the band could be mitigated by splitting the band into 8 NFDM tributaries and perform power loading, see Supplementary Table 4. The optimal center frequency of 226 GHz was chosen to maximize the data rate through the channel. Supplementary Figure 6 shows the typical frequency-dependent saturated output power and 1 dB compression point (P1dB) of the sub-THz amplifiers, provided by Virginia Diodes[18]. As P1dB increases at lower carrier frequencies, distortion is reduced, enabling the use of higher-order modulation formats for tributaries 1–4, see Figure 4.

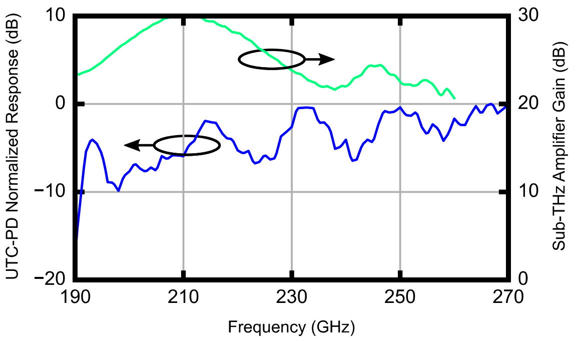

Supplementary Fig. 5: Normalized response of the sub-THz components. The normalized response of the uni-travelling photodiode (UTC-PD) is shown in blue while the gain spectrum of the sub-THz amplifier is shown in green.

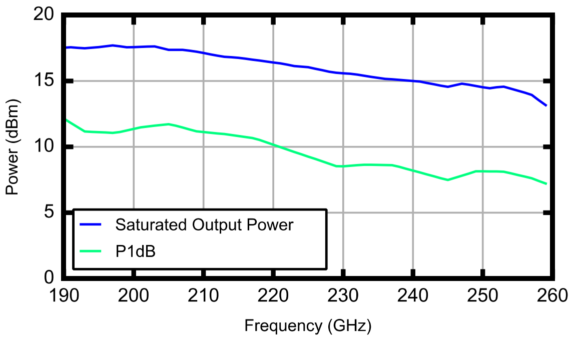

Supplementary Fig. 6: Saturated output power and 1 dB compression point (P1dB) of the sub-THz amplifier. The values shown represent typical device performance rather than that of the specific amplifier used in this work and are provided by VDI[18].

Supplementary References

1. Balanis CA. *Antenna Theory: Analysis and Design, 3rd Edition* (2005).

2. Recommendation ITU. Recommendation ITU-R P.676, Attenuation by Atmospheric Gases. (2012).

3. Feng XL*, et al.* Experimental demonstration of bidirectional up to 40 Gbit/s QPSK coherent free-space optical communication link over ~1 km. *Optics Communications* **410**, 674-679 (2018).

4. Trichili A, Cox MA, Ooi BS, Alouini MS. Roadmap to free space optics. *Journal of the Optical Society of America B-Optical Physics* **37**, A184-A201 (2020).

5. Kim II, McArthur B, Korevaar E. Comparison of laser beam propagation at 785 nm and 1550 nm in fog and haze for optical wireless communications. *Optical Wireless Communications* **4214**, 26-37 (2001).

6. Jing QF, Liu DM, Tong JC. Study on the Scattering Effect of Terahertz Waves in Near-Surface Atmosphere. *Ieee Access* **6**, 49007-49018 (2018).

7. S. Bloom EKJSHW. Understanding the Performance of free-space optics [Invited]. **2**, 178-200 (2003).

8. Schneider T, Wiatrek A, Preussler S, Grigat M, Braun RP. Link Budget Analysis for Terahertz Fixed Wireless Links. *Ieee Transactions on Terahertz Science and Technology* **2**, 250-256 (2012).

9. Fiorino ST*, et al.* A first principles atmospheric propagation & characterization tool - the Laser Environmental Effects Definition and Reference (LEEDR). *Atmospheric Propagation of Electromagnetic Waves Ii* **6878**, (2008).

10. Soni GG, Tripathi A, Shroti M, Agarwal K. Experimental study of rain affected optical wireless link to investigate regression parameters for tropical Indian monsoon. *Optical and Quantum Electronics* **55**, 1-10 (2023).

11. Norouzian F*, et al.* Rain Attenuation at Millimeter Wave and Low-THz Frequencies. *Ieee Transactions on Antennas and Propagation* **68**, 421-431 (2020).

12. Amarasinghe Y, Zhang W, Zhang R, Mittleman DM, Ma J. Scattering of Terahertz Waves by Snow. *Journal of Infrared, Millimeter, and Terahertz Waves* **41**, 215-224 (2019).

13. Ma JJ, Moeller L, Federici JF. Experimental Comparison of Terahertz and Infrared Signaling in Controlled Atmospheric Turbulence. *Journal of Infrared Millimeter and Terahertz Waves* **36**, 130-143 (2015).

14. Al-Habash MA, Andrews LC, Phillips RL. Mathematical model for the irradiance probability density function of a laser beam propagating through turbulent media. *Optical Engineering* **40**, 1554-1562 (2001).

15. Gao W, Han C, Chen Z. Scintillation and Attenuation Modelling of Atmospheric Turbulence for Terahertz UAV Channels.) (2024).

16. Akinwumi JA, Bandele JO. *Free Space Optical Communication : Review Paper* (2018).

17. Kikuchi K, Tsukamoto S. Evaluation of sensitivity of the digital coherent receiver. *Journal of Lightwave Technology* **26**, 1817-1822 (2008).

18. Waveguide Amplifiers Operational Manual. https://vadiodes.com/wp-content/uploads/2026/01/VDI-792.34-VDI-Waveguide-Amplifier-Product-Manual-1.pdf, (accessed 2026).
