# Supplementary material for: Plasmonic modulator enabling kilometer-range high-throughput sub-THz links for radio access networks
Source: Nat Commun. 2026 Apr 18;17:5384. doi: 10.1038/s41467-026-72053-z (PMC13275729; doi:10.1038/s41467-026-72053-z)
Supplement: Supplementary file 2 — Transparent Peer Review file [file 41467_2026_72053_MOESM2_ESM.pdf]

nature portfolio

Peer Review File

Plasmonic Modulator Enabling Kilometer-Range High-Throughput Sub-THz Links for Radio Access Networks

Corresponding Author: Mr Boris Vukovic

This file contains all reviewer reports in order by version, followed by all author rebuttals in order by version.

Version 0:

Reviewer comments:

Reviewer #1

(Remarks to the Author)
The paper reports the achievement of a THz/optical link using plasmonic MZM, and including tests on the field (outdoor). While the paper is interesting, I am not sure to catch the main claims. So far, the work is a combination of several building blocks that are already reported by the same authors, with a new study related to the atmospheric effects. I don't fully understand why the plasmonic modulator approach is helping to analyze the resilience of the THz link over propagation in the atmosphere. To my opinion, the main findings and step forward compared to SoTA should be more clearly claimed to ensure the paper reach good impact. Some comments related to this:
About comparison with FSO, the higher robustness of the THz transmission is expected and was experimentally demonstrated already in 2012 by Moeller & Federici (DOI: 10.1364/JOSAA.29.000179) and several recent studies reported this as well, as an example:
Jianjun Ma, Yuheng Song, Mingxia Zhang, Guohao Liu, Weiming Li, John F. Federici, Daniel M. Mittleman, Terahertz channels in atmospheric conditions: Propagation characteristics and security performance, Fundamental Research, 2024.
In order to assess the effects of the atmospheric transmission and discuss the atmospheric contribution, a back-to-back transmission without the air link would be necessary, to highlight the effect of the turbulences and also the stability of the EVM, SNR of the THz link itself, but without air propagation.
This also stands for the system optical performance: it should also be discussed to properly compare the FSO performance with the THz one in a clear sky condition or with a back-to-back optical transmission.
If the focus is to study the effects of the THz signal, why not detecting directly the THz signal at 226 GHz? A lot of receivers in the THz range are available now. If the main claim is the use of the plasmonic modulator that helps to study the atmospheric effect, this should be discussed.
In the figure 5, the I/Q maps and SNR correspond to optimal situation or worse case? This is not clear here. I would expect to see some BER variations linked to weather variations here. If the study aim to link atmospheric effects to the THz link performance compared to FSO, impact on the system metrics (like BER) should be shown.
About the product data-rate.distance, a full comparison for the readers needs to include additional metrics. In the figure 6, in order to compare the system to the other works, the EVM (%) or BER (Bit Error Rate) achieved should be mentioned. This is also applicable in the table, where the EVM (% or dB) and BER should be included. Here the authors measured the BER performance of their system thus they can compare their performance with others.
About the distance, as 12 dBm of output power is used using amplifier, the transmission distance is obviously enhanced compared to short distance demos, I think this is more an engineering step forward rather that a new finding.
I have also some technical questions that are not described:
- How the SNR is calculated?
- Why the tap number of the FFE is very high compared to CMA and RDE?
- The received signals show very noisy I/Q maps with limited signal to noise ratio. Did the authors analyzed the origin of this noise (or how they confirm that the ASE noise is the main limitation)?
- It is said that main noise source in the system is the EDFA noise (ASE), what is the noise figure of this EDFA?
- As mentioned by authors, the plasmonic modulator is one of the important part of the system, however I didn't find any details of the impedance matching, the interconnection of the 226 GHz for modulator driving as well as the driving power to the modulator. I believe this is important parameters and this should be described. Also, the noise figure/ the P1dB (compression point) of the amplifiers used should be discussed as this typically impacts a lot the performances of the overall system as the noise figure of THz amplifier is yet not very good.

Reviewer #2

(Remarks to the Author)
I've reviewed the paper titled "Photonic-Plasmonic Sub-THz Wireless Link for 6G Demonstrating 160 Gbps over 1400 m in Strong Atmospheric Turbulence" submitted to Nature Communication. This is an extension work which previously reported in OFC 2024. The results are solid, the topics is of significance to next generation network. However, the paper's innovation requires further clarification, and several details need to be addressed to strengthen the presentation.

General comments:
1.The primary contribution of this paper is the outdoor system demonstration of a sub-THz link over 1.4 km using Plasmonic MZMs. There are already 500GHz THz-to-optical conversion by plasmonic MZM as reported in Ref. 24, and also some experimental demonstrations been demonstrated in Ref. 28. The novelty of this work compared to these references remains unclear. Have there been any device-level improvements? The paper should clearly articulate its advancements over the state-of-the-art.
2.The title may cause confusion among readers. It is well established that atmospheric turbulence is generally not an issue for millimeter-wave (MMW) and THz signals but poses significant challenges for free-space optical (FSO) channels. The current title might mislead readers to believe that turbulence has become a problem for THz communication or that the paper solves the turbulence issue for FSO. Clarifying this or change the title is recommended.
3.While the authors compare THz and FSO performance under turbulent conditions, no clear technical contributions or innovations are evident in this comparison. Section III, "Influence of Atmospheric Turbulence on Wireless Link," merely reiterates a well-known result. Therefore, the innovation or contributions requires further clarification in this section.
4.The authors employ terms like All-E (all-electronic), P-E (photonic-electronic), and P-P (photonic-plasmonic) to describe system setups. For instance, P-E refers to a photonics-based transmitter (UTC-PD) and an electronics-based receiver. However, the term "photonic-photonic" would be more appropriate as it encompasses a broader range of solutions, whereas "photonic-plasmonic" excludes other photonic up (or down)-conversion methods, such as thin-film lithium niobate-based THz down-conversion.
Some of the latest experimental achievements in all-photonic up- and down-conversion solutions have not been included. Relevant references such as:
P. T. Dat et al., "151.5-GHz Sub-THz Signal Reception and Down-Conversion Using All-Optical Technology," 2024 Optical Fiber Communications Conference and Exhibition (OFC), San Diego, CA, USA, 2024, pp. 1–3.

5.Additionally, Table 2 only lists the distance-rate product for frequencies above 100 GHz, omitting notable demonstrations around 100 GHz in the E or W band (75–110 GHz). Including these would provide a more comprehensive comparison.

Major comments:
6.In the dual-band receiver, which factor has a greater impact on system performance: the insertion loss caused by the wavelength demultiplexer or the gain provided by combining two sidebands? How can these factors be balanced in the system design?
7.How were the atmospheric conditions' effects on sub-THz propagation experimentally verified? Specifically, under high humidity, were there significant signal attenuation changes? How does the system design compensate for these variations?
8.What are the bandwidth and operational frequency range of the UTC-PD and sub-THz amplifiers used in the system? Were the gain spectrum, frequency response, and attenuation characteristics of these components considered when selecting the central frequency? Is the chosen central frequency optimized for the system's best performance?
9.The authors state, "since the free-space channel is linear, only linear equalizers were required." However, this is not true. Other system components could also introduce nonlinear distortions? There is no discussion of nonlinear processing in the DSP section. Given the use of high-gain sub-THz amplifiers, could nonlinearities be introduced in these components? If so, how are these nonlinear effects addressed or mitigated in the system?
10.Deploying sub-THz links will require infrastructure such as transmitter/receiver installations, antenna alignment systems, and power supplies. What are the practical challenges and requirements for deploying these links in various environments? How can the process be streamlined to support large-scale rollouts for 6G networks?
11.The paper uses RF probes for signal modulation, rather than packaged devices. What methods are planned for packaging these devices in the future? What bandwidth losses are anticipated in the packaging process, and are there specific techniques to minimize degradation while maintaining performance?
12.The paper describes two photoelectric conversions, and from Figure 4, it appears that the two optical signals at the UTC-PD are not phase-locked. Similarly, the optical signals at the coherent receiver (Coh.Rx) also operate independently. This would result in poor phase noise in the electrical signals. Does the offline signal processing section address phase noise mitigation?
13.The demonstrated system has a low bandwidth utilization of the ultra-wideband MZM, and to improve the SNR, two coherent receivers were used. This trade-off in cost versus performance needs to be carefully analyzed. Could the system be scaled to higher THz center frequencies?.
14.It is recommended to provide key THz device parameters, such as operating frequency and bandwidth, to enhance the clarity of the demonstration.

Minor comments
15.Some typos: please check your definitions of up-mixer and down mixer. For example, the descriptions in Figure 4(a) are inconsistent with the subtitles. Should "Plasmonic Sub-THz Down-Mixer" be "Plasmonic Sub-THz Up-Mixer"?

Reviewer #3

(Remarks to the Author)
The authors present a photonic-plasmonic sub-THz wireless link and achieve 160-Gbps transmission over 1400 m. This result is remarkable; however, it is similar to the work presented at OFC 2024. It would be beneficial if the authors could extend this research further.
The authors have added discussions and comparisons with the FSO system; however, no solutions are proposed to overcome the limitations of each technology. Additionally, the two works presented in the manuscript are not closely related to each other. It would be better to separate them into two papers, each with extended results.
It is evident that RF systems, including those in the THz band, are robust to atmospheric turbulence due to the nature of signal reception. RF signals are received using large-diameter antennas. In contrast, FSO systems are more affected by atmospheric turbulence due to the use of very small-diameter photodetectors for signal detection. It is also well known that aperture averaging with a large-diameter lens can reduce the effects of turbulence. This effect is particularly significant when using direct fiber coupling at the receiver, as the incoming free-space signals are directly focused into a 10-μm core fiber. The turbulence can alter the angle of arrival of these signals. Given these factors, it is clear that the impact of turbulence in RF systems is minimal.
For the data shown in Fig. 7, why does the scintillation index for the FSO system remain unchanged during the measurement period, while the scintillation index for the THz system varies? What were the weather conditions and temperature at the time of the measurement? Typically, turbulence is stronger in hot weather, and it is usually most intense around noon. If the scintillation index (SI) of the FSO system does not change, it indicates that the atmospheric turbulence remained relatively constant during the measurement period. In that case, we cannot conclude that the experiment was conducted under strong turbulence conditions, as stated in the paper. The change in the scintillation index (SI) of the THz system could be due to other factors, such as wind effects, power fluctuations in signal generation and transmission, and the overall stability of the systems. It is recommended that additional measurements be taken over multiple days and under varying weather conditions. Additionally, more data should be recorded and analyzed throughout a single day. A comparison of measurements taken over the same link would help reach a more precise conclusion.

Version 1:

Reviewer comments:

Reviewer #1

(Remarks to the Author)
Authors added several details as requested. I think there are some errors in the text mixing dB and dBm. Authors should check them carefully. As an exemple, "9 dB" in line 322 should be 9 dBm I think.

I am still a bit worried about the non-linear and noise analysis done, as authors used THz/sub-THz amplifiers. It is quite difficult here to assess the real contributions of these amplifiers, making comparisons somehow difficult. Can authors update this point?

Reviewer #2

(Remarks to the Author)
The authors have revised the manuscript as requested, and the overall quality has improved. However, upon careful re-reading, several issues remain that need to be addressed before the paper meets the rigorous standards of Nature Communications.
1. As noted by all reviewers, the manuscript title has been revised as requested. However, I strongly recommend removing numerical values from the title to prioritize emphasis on the innovative concepts—particularly since the reported distance is not exceptional when compared to other published works.
2. Regarding new measurements of environmental parameters along the channel, the authors report the 24-hour variations of temperature and relative humidity at the transmitter. For a long-distance transmission experiment, environmental conditions along the propagation path are equally important; segment-wise measurements along the link would allow a more precise attribution of environmental effects. In this experiment, did the authors also measure the relevant parameters at the receiver? Are the variations consistent with those at the transmitter, and do they show consistent trends in their impact on the link?
3. For the turbulence strength, the authors compute it from the power fluctuations of the optical signal modulated by the received THz signal. While such fluctuations may partially reflect variations in the received THz power, how accurate is this power-based approach for evaluating both the THz and FSO links? How does it compare with directly measuring power using a THz power meter at the THz receiving antenna?
4. The authors state that they have replaced “photonic-plasmonic” with “all-photonic” throughout the manuscript. However, footnote “a” below Table 2 still annotates “P–P” as “photonic-plasmonic.” Please carefully check the entire manuscript for similar instances and ensure consistency. Furthermore, the types of all-electronic systems should be consistent (e.g., using “All-E” throughout instead of a mix of “All-E” and “E-E”) to prevent confusion.

5. The authors have added the BER and FEC overhead from previous works in Table 2 but have not specified the BER threshold and corresponding overhead used in this paper. It is unfair to only compare the line rate or line rate-distance product, with very different BER thresholds. The author should give clear net-data rate after the FEC corrections. In addition,

the BER results in Figure 6(b) appear not to meet common pre-FEC BER thresholds (e.g., 1e-2 or 2e-2). The authors must explicitly state what kind of BER overhead used to justify the achieved line rate and net data rate, ensuring the claim is convincing.

Reviewer #3

(Remarks to the Author)
The authors have made efforts in responding to comments and revising the manuscript. However, the paper remains unclear. It is difficult to determine the main focus—whether it is on high-capacity, long-distance transmission, or on a comparison with the FSO link. While several results and discussions on the effects of atmospheric turbulence on THz and FSO links are presented, along with their comparison, these appear rather trivial and not significant enough to be included in a main Nature Communications paper. Moreover, the revised title does not fully reflect the scope of the work.
The authors are advised to substantially reorganize the paper, focusing on high-data-rate and long-distance transmission. However, more details and extensions should be provided compared to their previous work, including the OFC 2024 paper [36] and the Optics Express paper [33]. The influence of atmospheric turbulence on the THz link can then be included to highlight its advantages over the FSO link. I suggest that the discussion and theoretical results currently presented in the "Comparison of Sub-THz and FSO Communications" section be moved to the Supplementary Information.
Additional technical comments:
1. In Fig. 7(a), is there an underlying physical explanation for why the scintillation index of the THz link does not saturate in the same way as the FSO link?
2. The authors state that a short-link experiment was previously conducted to confirm that no power fading is caused by the system itself. However, system stability depends heavily on operational conditions—such as cable placement, temperature, and vibrations. In particular, for field operations, the conditions differ from those of short-link experiments conducted indoors. Given these factors, it is difficult to conclude that the system is completely stable without any power fading. Therefore, results under different turbulence conditions should be analyzed and compared to confirm the actual effect of turbulence on the THz links.
3. For the THz link, wind can have a significant impact, potentially causing power fading due to misalignment of directive antennas. While the authors present temperature and humidity data, measured wind speed at the time of measurement should also be included and discussed.

Version 2:

Reviewer comments:

Reviewer #1

(Remarks to the Author)
The corrections and suggestions were taken into account, but one thing is not technically clear in my understanding of the paper.

Authors said that output power at point C (Figure 3) is 12 dBm, ie 3 dB beyond amplifier P1dB which is 9 dBm. Operating a QAM-16 3 dB beyond P1dB induces that the QAM-16 signal should be commpressed a lot, which is not the case. This is surprising as any-non linear equalization is done. Authors should cross-check before publication.

Reviewer #2

(Remarks to the Author)
The revised manuscript title now better highlights the technical innovation. The overall quality paper after revision has been improved a lot. However, I suggest further removing "for 6G Networks". Since the use of the sub-THz band in 6G is not yet definitively standardized and the demonstrated modulator/link represent the physical-layer advancement, a more general title would avoid limiting the work's applicability. Additionally, to maintain consistency throughout the manuscript and to ensure the focus remains on the innovative concepts, I recommend that the authors also revise any similar statements in the abstract and main text that may overly associate this work exclusively with 6G.

Reviewer #3

(Remarks to the Author)
The authors have made efforts to improve the manuscript in response to the reviewer's previous comments. By moving the "Comparison of Sub-THz and FSO Communications" section to the Supplementary Information and revising the title, the manuscript is now more focused and easier for readers to follow.
Although the authors disagreed with some of the reviewer's earlier comments, the reviewer's intention was not to impose additional burdens or challenges, but rather to help strengthen the manuscript. If the authors and the editor consider the current content, largely based on the OFC 2024 paper, sufficient for publication in Nature Communications, the reviewer has no objection.

Minor comments on the revised manuscript:

- The section titles “Influence of Atmospheric Turbulence on Wireless Links” (main text) and “Influence of Atmospheric Turbulence on Sub-THz Links” (Supplementary Note 2) are very similar. The authors are encouraged to revise one of these titles to better distinguish them.
- The “Discussion” section in the main text is quite brief. It would be helpful to include additional details on packaging approaches and the development of the modulator module to support practical implementation.

Version 3:

Reviewer comments:

Reviewer #1

(Remarks to the Author)
The authors clarified the issue with the output power, it is ok now.

Open Access This Peer Review File is licensed under a Creative Commons Attribution 4.0 International License, which permits use, sharing, adaptation, distribution and reproduction in any medium or format, as long as you give appropriate credit to the original author(s) and the source, provide a link to the Creative Commons license, and indicate if changes were made.
In cases where reviewers are anonymous, credit should be given to 'Anonymous Referee' and the source.
The images or other third party material in this Peer Review File are included in the article’s Creative Commons license, unless indicated otherwise in a credit line to the material. If material is not included in the article’s Creative Commons license and your intended use is not permitted by statutory regulation or exceeds the permitted use, you will need to obtain permission directly from the copyright holder.
To view a copy of this license, visit https://creativecommons.org/licenses/by/4.0/

Below are detailed responses to each of the reviewer's comments (italic gray). Additionally, alterations made to the initial manuscript are clearly marked in green (additional) and red (removal) which are directly added below each comment.

Reviewer 1

We thank the reviewer for his overall assessment of our work. We are happy to see that the reviewer finds the paper of interest. We regret that the main points of our manuscripts have not been communicated clearly enough and that the SOTA comparison has been lacking. We answer below the reviewer's comments below in detail to clarify these open points:

The paper reports the achievement of a THz/optical link using plasmonic MZM, and including tests on the field (outdoor). While the paper is interesting, I am not sure to catch the main claims. So far, the work is a combination of several building blocks that are already reported by the same authors, with a new study related to the atmospheric effects. I don't fully understand why the plasmonic modulator approach is helping to analyze the resilience of the THz link over propagation in the atmosphere. To my opinion, the main findings and step forward compared to SoTA should be more clearly claimed to ensure the paper reach good impact.

We are happy to see that the reviewer finds the paper of interest. We regret that the main points of our manuscripts have not been communicated clearly enough. The novelties are as follows:

- This is the highest distance-data-rate product of a sub-THz free-space link
- The success is in part a combination of multiple factors
  - Use of plasmonic components that offer a flat frequency response up to highest speed.
  - Development of system-level improvements based on a novel dual-sideband receiver to increase the SNR and therefore data rate.
- Analysis and measurement of the effect of turbulences on a high data rate 200-300 GHz sub-THz signal in a field trail.

We want to stress that there is no claim that the plasmonic modulator helps to study the atmospheric effect. We apologize that the manuscript failed to communicate the main claim clearly. Plasmonic components help in performing communications at sub-THz frequencies because they offer a flat frequency response up to 1 THz and directly up-mix the sub-THz signal into the optical domain.

To stress the achievement of highest distance-rate product of this work more, we restructured the manuscript to show the comparison of state-of-the-art (Figure 2) at the beginning of the results section.

We have revised the manuscript as follows:

Title:

Photonic-Plasmonic Sub-THz Wireless Link for 6G Demonstrating 160 Gbps over 1400 m in Strong Atmospheric Turbulence

All-Photonic Sub-THz Wireless Link Demonstrating 160 Gbps over 1400 m for 6G Radio Access Networks

	Abstract:<br>[…] Broadband photonic and plasmonic components enabled flat frequency response at high speeds. […] (P.1, L.24)<br><br>[…] We assessed power variations due to atmospheric turbulence; the scintillation index remained below 0.019 under strong turbulence, confirming sub-THz link resilience. A theoretical comparison […] (P.1, L.26)<br><br>Introduction:<br>[…] The receiver utilizes direct conversion through an organic-plasmonic Mach-Zehnder modulator (MZM) and a dual-sideband receiver scheme that provides a SNR gain of up to 2 dB[33]. The receiver utilizes direct conversion through an organic-plasmonic Mach-Zehnder modulator (MZM). Compared to prior art, a dual-sideband receiver scheme that provides a SNR gain of up to 2 dB[33] has been employed. We report outdoor measurements of atmospheric turbulence effects on a high data rate sub-THz link, with scintillation indices staying below 0.019 even under strong turbulence. These results confirm the known resilience of sub-THz links to turbulence[34, 35] and, unlike many lab-based studies, provide valuable evidence to the community that sub-THz links perform reliably under real-world outdoor conditions over practical distances exceeding 1 km. For the first time outside laboratory conditions, we measured the impact of atmospheric turbulence on received sub-THz power, with the scintillation index remaining below 0.019 even in strong turbulence. […] (P.3, L.35)<br><br>Results:<br>[…] Sub-THz frequencies may offer a balance of high throughput and resilience towards adverse weather, making them an ideal complement to FSO to ensure high-capacity, reliable wireless communications.<br><br>The seamless transition from the optical domain to the sub-THz domain is enabled by the high-speed sub-THz down- und up-mixer as illustrated in Figure 1c. In case of adverse weather conditions, where FSO is blocked, the optical signal at the transmitter side can be re-routed to the sub-THz down-mixer and transmitted via a sub-THz carrier. On the receiver side, the signal is directly up-mixed back to the optical domain by means of a high-speed plasmonic modulator.<br>A high-speed sub-THz down- and up-mixer enables a seamless transition between the optical and sub-THz domains, as shown in Figure 1c. During adverse weather conditions that disrupt free-space optical (FSO) links, the optical signal at the transmitter can be rerouted through the sub-THz down-mixer and sent over a sub-THz carrier. At the receiver, the signal is directly up-mixed back into the optical domain using a high-speed plasmonic modulator. This seamless switching capability allows the optical and sub-THz links to complement each other in a confluent way, effectively addressing the limitations of each technology.

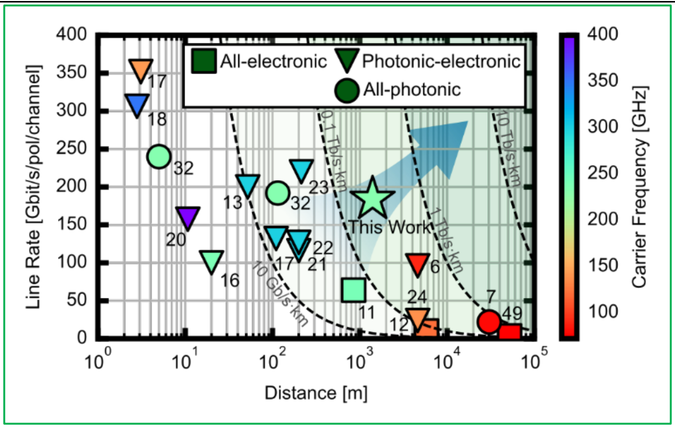

Fig. 2: ***State-of-the-Art THz-band (0.3-10 THz), sub-THz-band (100-300 GHz), E-band (60-90 GHz) and W-band (75-110 GHz) links.*** *Line rate per polarization and channel versus transmission distance of state-of-the-art wireless links. The color represents the carrier frequency used in the experiment.*

To show the potential of the all-photonic links, we plot in Figure 2 the transmission distance and achieved line rates for a selection of state-of-the-art all-electronic, photonic-electronic, and all-photonic link demonstrations. Further details to each demonstration are summarized in Table 2. This work shows that high data rate and long distance sub-THz links are indeed achievable by using photonic and plasmonic components. While raising the carrier frequency above 100 GHz brings the advantage of higher bandwidth, it raises a set of technological and operational challenges that need to be addressed. Firstly, we discuss the theoretical considerations for a long distance sub-THz link like atmospheric attenuation and turbulence, including a comparison to FSO communication. Secondly, we demonstrate in a field trial over 1400 m the sub-THz link demonstration (above 100 GHz) with the highest product of line-rate and distance to date, which is enabled by high-speed plasmonic modulators and the dual-sideband receiver. […] (P.5, L.9)

1.*[…] About comparison with FSO, the higher robustness of the THz transmission is expected and was experimentally demonstrated already in 2012 by Moeller & Federici (DOI: 10.1364/JOSAA.29.000179) and several recent studies reported this as well, as an example: Jianjun Ma, Yuheng Song, Mingxia Zhang, Guohao Liu, Weiming Li, John F. Federici, Daniel M. Mittleman, Terahertz channels in atmospheric conditions: Propagation characteristics and security performance, Fundamental Research, 2024. […]*

We thank the reviewer for this comment and the references. We have now referenced these papers. We also agree that there are published studies on atmospheric turbulence and that it is a known result, that sub-THz is more resilient to atmospheric turbulence compared to FSO. However, to the best of our knowledge, all these studies use turbulence chambers to precisely control the conditions in a reproducible way. We believe that measurements of the effect of atmospheric turbulence in a field trial transmitting high data rates over kilometer distances, as one would encounter in a deployed link, are valuable to the community.

We included the reference and adjusted the introduction to stress this point in the following way:

Introdcution:

[…] We report outdoor measurements of atmospheric turbulence effects on a high data rate sub-THz link, with scintillation indices staying below 0.019 even under strong turbulence. These results confirm the known resilience of sub-THz links to turbulence[34, 35] and, unlike many lab-based studies, provide valuable evidence to the community that sub-THz links perform reliably under real-world outdoor conditions over practical distances exceeding 1 km. For the first time outside laboratory conditions, we measured the impact of atmospheric turbulence on received sub-THz power, with the scintillation index remaining below 0.019 even in strong turbulence. […] (P. 3, L. 40)

[…] In order to assess the effects of the atmospheric transmission and discuss the atmospheric contribution, a back-to-back transmission without the air link would be necessary, to highlight the effect of the turbulences and also the stability of the EVM, SNR of the THz link itself, but without air propagation. […]

We agree with the reviewer, that a back-to-back transmission of the THz link without the air channel are needed to precisely isolate the effect of the sub-THz channel. We have indeed performed short distance indoor experiments to confirm the stability of the system before going to the field trial. Some of these short distance results have been published by members of our group:

Horst Y*, et al.* Transparent Optical-THz-Optical Link at 240/192 Gbit/s Over 5/115 m Enabled by Plasmonics. *Journal of Lightwave Technology* **40**, 1690-1697 (2022).

With respect to the comment on the EVM and SNR properties: We answer this comment further below in more detial

We adjusted the manuscript in the following way to emphasize this point:

Results:

[…] instances during the day using a power meter with an averaging time of 200 µs. To confirm that the origin of the power fluctuations stem from the free-space channel, short range indoor experiments in the laboratory over 5 m have been conducted in prior studies[32], showing that the received power is stable. Therefore, the power fluctuations in this study are attributed to atmospheric turbulence effects. We use a $\Gamma\Gamma$-distribution and Andrew's method[47] to extract the […] (P.15, L.8)

[…] This also stands for the system optical performance: it should also be discussed to properly compare the FSO performance with the THz one in a clear sky condition or with a back-to-back optical transmission. […]

We thank the reviewer for his commentary on the comparison of an FSO link to a sub-THz link under more weather conditions. We note that clear sky conditions can still include various forms of turbulences due to temperature gradients, winds, relative humidity, etc. We therefore expanded on our theoretical analysis on how a sub-THz link compares to the FSO link.

We added the new subplots Fig. 3c and 3d that illustrate the advantages in scintillation index and fading loss by comparing their calculated values.

While beyond the scope of this paper, it would indeed be preferable that future studies could directly compare all systems side by side: fiber link, FSO link and sub-THz link where we monitor the stability of each property over extended periods of time.

Results:

[…] adaptive optics. Figures 4c and 4d indicate that, under the aforementioned conditions, the scintillation index and fading losses fall within the green-shaded region, indicating a favorable regime for sub-THz relative to FSO links. More information on the calculations can be found in the Supplementary Note 1. […] (P.8, L.28)

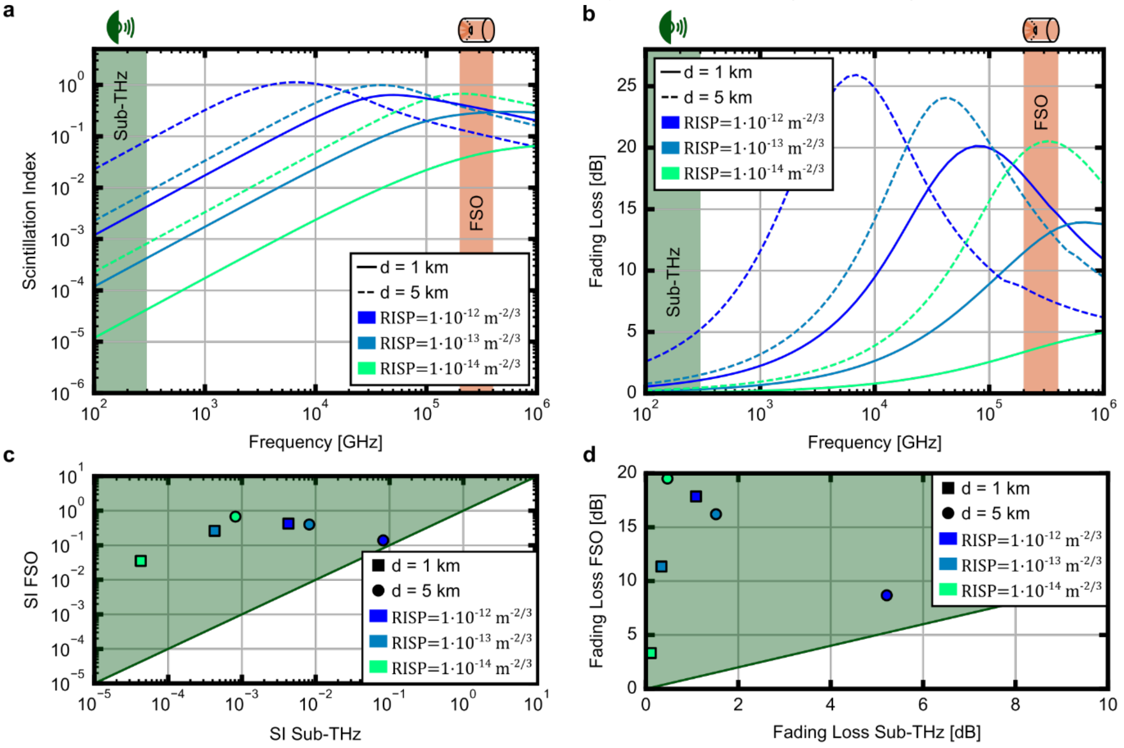

Fig. 4: ***Comparison of scintillation index (SI) and fading loss according to Andrew's method between sub-THz and free-space optics (FSO) in weak, moderate, and strong turbulence conditions at link distances of 1 km and 5 km assuming a receiver aperture radius of 2.5 cm. a*** *Scintillation index for three turbulence conditions, where the turbulence strengths relate to the refractive index structure parameter values (RISP).* ***b*** *Additional fading loss that needs to be compensated to have a 99.99 % reliability of the wireless link.* ***(c, d)*** *Comparison of SI and fading loss between FSO (1550 nm) and Sub-THz (300 GHz) in above mentioned conditions. The green shaded area is where sub-THz is advantageous compared to FSO.*

[…] If the focus is to study the effects of the THz signal, why not detecting directly the THz signal at 226 GHz? A lot of receivers in the THz range are available now. If the main claim is the use of the plasmonic modulator that helps to study the atmospheric effect, this should be discussed. […]

We want to stress that there is no claim that the plasmonic modulator helps to study the atmospheric effect. We apologize that the manuscript failed to communicate the main claim clearly. Plasmonic components help in performing communications at sub-THz frequencies because they offer a flat frequency response up to 1 THz and directly up-mix the sub-THz signal into the optical domain.

We have revised the manuscript as follows:

Title:

Photonic-Plasmonic Sub-THz Wireless Link for 6G Demonstrating 160 Gbps over 1400 m in Strong Atmospheric Turbulence

	All-Photonic Sub-THz Wireless Demonstrating 160 Gbps over 1400 m for 6G Radio Access Networks<br><br>Abstract:<br>[…] We assessed power variations due to atmospheric turbulence; the scintillation index remained below 0.019 under strong turbulence, confirming sub-THz link resilience. A theoretical comparison […] (P.1, L.26)
2.<br>*In the figure 5, the I/Q maps and SNR correspond to optimal situation or worse case? This is not clear here.*	
	We thank the reviewer for this comment. The I/Q maps and SNR correspond to the best case situation, where scintillation is at a minimum.<br><br>We adjusted the manuscript and the caption of Figure 5 in the following ways:
	Results:<br>[…] combination (purple) in best case conditions where scintillation was at a minimum. The combined SNR of the tributaries with lower baseband power could be improved by the dual-sideband receiver scheme. **b** Constellation […] (P.12, L.35)<br><br>[…]. In Figure 6b, we show the constellation diagram, the bit-error rate (BER) and general mutual information (GMI) of each tributary. These measurements are performed in best case conditions, where scintillation was at a minimum. The achievable information rate (AIR) is […] (P.13, L.28)
[…] I would expect to see some BER variations linked to weather variations here. If the study aim to link atmospheric effects to the THz link performance compared to FSO, impact on the system metrics (like BER) should be shown. […]	
	We agree with the reviewer that measurements of the BER in different atmospheric turbulence conditions would be valuable to study the impact on link performance. This paper shows the potential of the technology and is a demonstrates one of the highest distance-data rate products so far shown. As discussed above, a more thorough is planned to fully assess the stability of the BER in many more conditions.<br><br>We adjusted the manuscript and the following way:
	Discussion:<br>[…] effects on a THz link, showing low scintillation index of 0.019 even under strong turbulence. The promising performance observed in this study highlights the need for future work involving long-term sub-THz link deployment and assessment of BER and EVM across diverse weather conditions. […] (P.17, L.42)
3<br>*About the product data-rate.distance, a full comparison for the readers needs to include additional metrics. In the figure 6, in order to compare the system to the other works, the EVM (%) or BER (Bit Error Rate) achieved should be mentioned. This is also applicable in the table, where the EVM (% or dB) and BER should be included. Here the authors measured the BER performance of their system thus they can compare their performance with others.*	
	We agree with the reviewer, that adding BER and EVM helps comparing the performance of each link.

Table 2 in the manuscript has been adjusted by adding the pre-FEC BER, together with the type of FEC (HD-FEC/SD-FEC), the overhead in percent and if the FEC is performed online or offline, if this information is available in the publications. As almost none of the publications give information about the EVM, we decided to only show and compare the BER in Table 2 to keep the table readable.

Results:

Type[a]	Line-rate-distance product	Line rate [Gbit/s/pol/	Distance [m]	Carrier frequency [GHz]	BER (pre-FEC)	Technology TX/RX	Modulation Format	Year	Author	Reference
P-E	0.8568	306	2.8	350	2.4E-02 (Offline)	UTC-PD/SBD	PS-64QAM-OFDM	2020	Jia et al.	18
P-E	1.0912	352	3.1	140	< 4E-02 (Offline)	UTC-PD/D-Band Mixer	64QAM-PS5.5	2019	Li et al.	17
P-P	1.2	240	5	231	4.6E-02 (Offline)	UTC-PD/PM	QPSK, 16QAM	2022	Horst et al.	32
P-E	1.67	157	10.7	400	2.4E-02 (Offline)	UTC-PD/SBD	16QAM	2022	Jia et al.	20
P-E	2	100	20	237.5	4.5E-03 (Offline)	UTC-PD/InGaAs MMIC	16QAM	2013	Koenig et al.	16
P-E	10.4	200	52	300	6E-03 (Offline)	UTC-PD/GaAs MMIC	16QAM	2024	Dittmer et al.	13
P-E	14.52	132	110	300	1.9E-03 (Offline)	UTC-PD/SBD	16QAM	2020	Harter et al.	19
P-P	22.08	192	115	231	3.2E-02 (Offline)	UTC-PD/PM	QPSK, 16QAM	2022	Horst et al.	32
P-E	23.2	116	200	300	2E-02 (Offline) (20% SD-FEC)	UTC-PD/HM	PDM-QPSK	2024	Cai et al.	21
P-E	25.3	127	200	300	-	UTC-PD/	PS-16QAM DP-OFDM	2024	Tong et al.	22
P-E	47.08	220	214	300	3.75E-03 (Online) (HD-FEC)	UTC-PD/SHM	32QAM	2024	Maekawa et al.	23
All-E	54.4	64	850	240	7.9E-05 (Online)	GaAs HEMT MMIC/"	QPSK	2015	Kallfass et al.	11
All-E	58	10	5800	120	-	InGaAs/InP HEMT MMIC/"	ASK	2010	Hirata et al.	12
P-E	110.4	24	4600	135	8.6E-03 (Offline) (15% SD-FEC)	-	OFDM-16QAM	2022	Li et al.	24
E-E	187.2	3.6	52000	72	7E-02 (Offline)	E-Band Mixer/''	64QAM	2024	Guo et al.	49
P-E	441.6	96	4600	87.5	2.4E-02 (Offline) (15% SD-FEC)	PD/E-Band Mixer	PDM-16QAM	2023	Li et al.	6
P-P	857.6	32	26800	76.0	2.0E-02 (Offline)	PD/Phase Modulator	QPSK	2024	Liu et al.	7

					(15% SD-FEC)					
P-P	257.6	184	1400	230	3.83E-02 (Offline)	UTC-PD/Plas. Modulator	QPSK, 8QAM, 16QAM	2024		This work

4

About the distance, as 12 dBm of output power is used using amplifier, the transmission distance is obviously enhanced compared to short distance demos, I think this is more an engineering step forward rather that a new finding.

We thank the reviewer for the comment, and we agree that higher distances require more output power and the use of amplifiers. As the scope of the journal also involves applied, and engineering sciences, we argue that the engineering aspect of this work is of value to the community. Compared to laboratory demonstrations, bridging the distance of 1.4 km involves engineering, like positioning and alignment of the antenna, the antenna themselves, the use of amplifiers, which introduce bandwidth limitations, noise and nonlinear effects that come with higher power. Furthermore, turbulence effects may limit the systems performance.

5

I have also some technical questions that are not described:
- How the SNR is calculated?

We thank the reviewer for this comment. We added the calculation of the SNR for each tributary in a new section "Calculation of SNR" in the supplementary material.

We adjusted the manuscript and supplementary information in the following way:

Supplementary Information:

Supplementary Note 2: Calculation of SNR

This section describes how the signal-to-noise ratio (SNR) of each tributary is calculated after digital signal processing (DSP). The SNR of the received tributary signal $Y = N(0, \sigma_s + \sigma_n)$ is calculated by comparing it to the transmitted signal $X = N(0, \sigma_s)$ and assuming white Gaussian noise (AWGN) is added to Y. The noise power N_0 is defined as

$$N_0 = \mathrm{Var}(Y - hX) = \mathrm{Var}(Y) + h^2\mathrm{Var}(X) - 2h\mathrm{Cov}(X, Y), \qquad (7)$$

where X is the normalized transmitted signal, Y is the normalized received signal and h a proportionality factor. The noise power N_0 is minimized by choosing $h = \mathrm{Cov}(X, Y)/\mathrm{Var}(X)$. Inserting h into (7) yields

$$N_0 = \mathrm{Var}(Y) - \frac{\mathrm{Cov}(X, Y)^2}{\mathrm{Var}(X)}. \qquad (8)$$

Calculating the SNR yields.

$$\mathrm{SNR} = \frac{\mathrm{Var}(Y) - N_0}{N_0} = \frac{\mathrm{Var}(Y)}{N_0} - 1 = \frac{1}{1 - \frac{\mathrm{Cov}(X, Y)^2}{\mathrm{Var}(X)\mathrm{Var}(Y)}} - 1. \qquad (9)$$

Results:
[…] The resulting baseband power spectral density is shown in Figure 6a (grey color), along with the SNRs for each tributary. The calculation of the SNR is described in Supplementary Note 2. The SNRs of the lower sideband (LSB) and upper sideband (USB) are also presented, […] (P.13, L.15)

6

- Why the tap number of the FFE is very high compared to CMA and RDE?

We thank the reviewer for this comment, and we agree that the tap number of the FFE is very high. The tap number of the FFE could potentially be scaled down and optimized.

We adjusted the manuscript in the following way:

[…] filter taps of the CMA, RDE, and FFE was set to 41, 41 and 223, respectively. The number of taps was not optimized and could potentially be reduced without loss of performance. Combining the two baseband signals was achieved by utilizing a butterfly extension of the FFE. […] (P.13, L.7)

7

- The received signals show very noisy I/Q maps with limited signal to noise ratio. Did the authors analyzed the origin of this noise (or how they confirm that the ASE noise is the main limitation)?

We thank the reviewer for this remark. The I/Q maps look noisy indeed. We could easily resort to I/Q maps that look less noisy by reducing the symbol rate. Yet, these diagrams show the community up to what speed error free operation (error free if FEC is applied) can be performed. Therefore, the I/Q maps are supposed to be taken at the point where we transition to the limit of where data can still be recovered.

With respect to the reviewer's question on the noise source: We analyzed the limiting noise sources in a link budget, which we added to the supplementary information. We found that the ASE-LO beat noise of EDFA 2, and noise of the second sub-THz amplifier are the dominant noise sources in the system. Additionally, we have analyzed the measured SNR for each tributary of both sidebands separately and the combined SNR. By employing the dual-sideband receiver and by comparing those SNRs we can distinguish that the noise source is either added prior of after the plasmonic modulator. If the dominant noise is added after the modulator, e.g. in the EDFA 2, the noise will be uncorrelated, and an SNR gain is observed. However, if the noise is already present in the RF signal prior to the modulator, the noise in both sidebands is correlated and receiving both sidebands will not give any SNR improvement. Therefore, in tributaries 5-8 the limiting noise is the ASE-LO beat noise of EDFA 2, while tributaries 1-4 show more correlated noise that results in less gain. Based on a link budget, we attribute the noise source to the sub-THz amplifier.

To make this clearer, we adjusted the manuscript in the following way:

Results:
[…] is the amplified spontaneous emission noise introduced by the low-noise EDFA 2 after point D, see Figure 5a. On the other hand, the dual-sideband receiver's SNR improvement of tributaries 1-4 was below 2 dB, indicating that the dominant noise in both sidebands is correlated and originates prior to the plasmonic modulator. This finding agrees well with the theoretical link budget, as discussed in the supplementary note 3. […] (P.13, L.22)

Supplementary Information:

Supplementary Note 3: Link Budget

Supplementary Table 3 presents the theoretical link budget for each tributary. Signal power spectral density (PSD) is propagated through the channel using both measured and theoretical power spectra of the system components and the free-space link, see Supplementary Figure 2 and Figure 7e. Noise from sub-THz amplifiers is added to the signal and propagated through the channel. The SNR of the preamplifier coherent receiver is limited by the ASE-LO beat noise and is estimated using

$$\mathrm{SNR} = \frac{P_s}{hf n_{sp} B}, \qquad \text{(S10)}$$

where P_s is the optical signal power, hf the photon energy, n_{sp} the SNR degradation from the shot-noise limit and B the bandwidth[5]. For high gain values, the SNR degradation of the amplifier chain is approximately $n_{sp} \approx F_{tot}/2$, where F_{tot} is the combined noise factor of the receiver chain, which consist of low-noise EDFA 2, loss of the demultiplexer and polarization beam splitter and preamplifier EDFA 3, see Figure 4d. According to the Friis formula, the total noise figure can be calculated by

$$F_{tot} = F_{EDFA2} + \frac{L_{DEMUX} F_{EDFA3} - 1}{G_{EDFA2}}, \qquad \text{(S11)}$$

where F_{EDFA2} and G_{EDFA2} are the noise factor and gain of the low-noise EDFA 2, L_{DEMUX} the loss of the demultiplexer and polarization beam splitter and F_{EDFA3} the noise factor of the preamplifier EDFA 3. To estimate the SNR after the dual-sideband receiver, the ASE-LO beat noise power was reduced by 3 dB. The dominant noise sources were the sub-THz amplifier and the low-noise EDFA 2 at the receiver. The dual-sideband receiver's SNR gain matches the measured results well, showing that tributaries with lower optical sideband power are primarily limited by ASE-LO beat noise and benefit the most from the dual-sideband approach.

Supplementary Tab. 3: Theoretical link budget calculation for each tributary.

	Unit	Tributary							
Tributary Number		1	2	3	4	5	6	7	8
Bandwidth	GHz	8	8	8	8	8	8	8	8
Optical Launch Power (point A)	dBm	3.2	1.6	-1.2	2.3	-0.7	1.4	0.9	4.5
Optical Launch SNR (point A)	dB	17.2	19.7	21.3	23.1	21.9	20.9	18.6	16.2
Sub-THz Launch Power (point C)	dBm	2.5	4.7	4.3	2.7	-0.3	0.0	-0.3	5.1
Sub-THz Launch SNR (point C)	dB	17.1	19.6	21.1	22.8	21.4	20.6	18.4	16.2
Free-Space Path Loss	dB	-141.5	-141.7	-142	-142.3	-142.6	-142.8	-143.1	-143.3
Attenuation	dB	-5	-4.5	-4.5	-4.6	-4.8	-5	-5.3	-5.7
Antenna Gain	dB	111.7	111.7	111.7	111.7	111.7	111.7	111.7	111.7
Excess Loss	dB	7.0	7.0	7.0	7.0	7.0	7.0	7.0	7.0
Sub-THz Received Power	dBm	-39.2	-36.8	-37.5	-39.4	-42.9	-43.1	-44.1	-39.1
Sub-THz Power at Modulator	dBm	-14.1	-9.9	-11.1	-15.0	-21.8	-23.6	-22.9	-19.2
Optical Sideband Power (point D)	dBm	-44.5	-40.3	-41.5	-45.4	-52.1	-54	-53.2	-49.6
SNR Single Sideband (point E/F)	dB	12.3	15.8	15.6	12.7	6.2	4.4	5.0	8.3
SNR Dual-Sideband	dB	13.5	16.8	16.9	14.5	8.3	6.5	7.0	10.0

8

- It is said that main noise source in the system is the EDFA noise (ASE), what is the noise figure of this EDFA?

We adjusted the manuscript in the following way, to add the noise figure and gain of the EDFA.

[…] The optical signal is amplified by a low-noise EDFA EDFA 2 with a gain of 35 dB and a noise figure of 3.4 dB, transmitted through 6 km of SSMF before it is received in the dual-sideband optical coherent receiver[33], emulating the […] (P.12, L.4)

9

- As mentioned by authors, the plasmonic modulator is one of the important part of the system, however I didn't find any details of the impedance matching, the interconnection of the 226 GHz for modulator driving as well as the driving power to the modulator. I believe this is important parameters and this should be described. […]

We thank the reviewer for this remark and apologize that we didn't describe this important aspect in the original manuscript.

	We adjusted the manuscript to describe the impedance matching and connection with the modulator in the following way. Furthermore, we added the "Sub-THz Power at Modulator" to the link budget in the Supplementary Note 3.
	Results:<br>[…] modulator[28] (MZM) via a ground-signal-ground (GSG) RF probe with a characteristic impedance of 50 Ω. The total sub-THz power at the plasmonic MZM was -5.6 dB. The electrodes of the MZM are modeled as a capacitive load, with an estimated capacitance of 5 fF. Operating well below the cut-off frequency, the MZM presents a high impedance and behaves effectively as an open circuit, resulting in the reflection of the incident RF power back toward the probe and amplifier. Under these conditions, the voltage amplitude at the modulator input is 0.3 V. The plasmonic MZM, which is biased to the null point through wavelength We thank the reviewer for this remark. […] (P.11, L.38)
10<br>*[…] Also, the noise figure/ the P1dB (compression point) of the amplifiers used should be discussed as this typically impacts a lot the performances of the overall system as the noise figure of THz amplifier is yet not very good.*	
	We thank the reviewer for this comment.<br><br>We added the saturated output power and P1dB of the sub-THz amplifier. We agree with the reviewer, that noise figure of these amplifiers is not very good yet. Unfortunately, the datasheet of the manufacturer didn't specify the noise figure of the amplifiers and to date, we don't have the equipment to characterize it by ourselves.<br><br>In our case, as we operate the sub-THz amplifier at the transmitter 4 dB below the saturated output power, we couldn't observe any non-linear distortions. This is underlined by two instances in the measured IQ diagrams.<br>• The symbols are equally spaced with gaussian noise which points away from non-linearities.<br>• Applying a Volterra algorithm with 1 second and 1 third-order tap does not improve the SNR pointing away from non-linearities.<br><br>We added the following sentences to the manuscript to point this out more clearly.
	Results:<br>[…] The sub-THz signal is amplified by a commercial electrical sub-THz amplifier with a gain of 24 dB, yielding a typical launch power of 12 dBm (point C). The sub-THz amplifiers at the down- and up-mixer have a saturated output power of 16 dBm and 1 dB compression point (P1dB) of 9 dB. […] (P.11, L.18)<br><br>[…] Combining the two baseband signals was achieved by utilizing a butterfly extension of the FFE. Despite the two sub-THz amplifiers in the link, non-linear equalization could not improve the SNR and BER as the amplifiers were not operated close to saturation. Therefore, non-linear equalization was omitted, reducing the computational complexity of the DSP. […] (P.13, L.9)

We thank the reviewer again for his constructive comments. By addressing each of the remarks and improving our manuscript with the provided feedback, we hope that now the paper clearly communicates the open points on the main achievements. Moving from distinct component based lab experiments to large, combined field trials show the potential and challenges of this

technology for future use in industry. Using an all-photonic approach with plasmonic modulators could show the highest distance-data rate product for sub-THz links (> 100 GHz carrier frequency).

Reviewer 2

I've reviewed the paper titled "Photonic-Plasmonic Sub-THz Wireless Link for 6G Demonstrating 160 Gbps over 1400 m in Strong Atmospheric Turbulence" submitted to Nature Communication. This is an extension work which previously reported in OFC 2024. The results are solid, the topics is of significance to next generation network. However, the paper's innovation requires further clarification, and several details need to be addressed to strengthen the presentation.

We are happy to see that the reviewer finds the results solid, and we are thankful for the comments, which improve the presentation of the paper. We address each of the reviewer's inputs in detail below.

1

The primary contribution of this paper is the outdoor system demonstration of a sub-THz link over 1.4 km using Plasmonic MZMs.
-There are already 500GHz THz-to-optical conversion by plasmonic MZM as reported in Ref. 24, and also some experimental demonstrations been demonstrated in Ref. 28.
The novelty of this work compared to these references remains unclear. Have there been any device-level improvements? The paper should clearly articulate its advancements over the state-of-the-art.

We thank the reviewer for the comment.

We would like to communicate the novelty of this paper more clearly, which are:

- This is the highest distance-rate product of a sub-THz free-space link
- The success is in part a combination of multiple factors
    - Use of plasmonic components that offer a flat frequency response up to highest speed.
    - Development of system-level improvements based on a novel dual-sideband receiver to increase the SNR and therefore data rate.
    - Assembly of the devices and components such as plasmonic modulators, antennas, antenna-positioners, etc. into a system demonstration in a field trial.
- Analysis and measurement of the effect of turbulences on a high data rate 200-300 GHz sub-THz signal in a field trial.

The advancement over state-of-the-art is the record high distance-rate product of a sub-THz free-space link. To stress this more, we restructured the manuscript to show the comparison of state-of-the-art (Figure 2) at the beginning of the results section.

Compared to Ref. 28, the dual-sideband receiver was developed and incorporated successfully in the system. Furthermore, the air channel is changed from 115 m indoors to 1.4 km outdoors, which comes with challenges, such as alignment, increased FSPL, attenuation, atmospheric turbulence, etc.

We do not report on any device level improvements.

To clarify these points, the manuscript has been adjusted in the following way:

Abstract:

[…] We assessed power variations due to atmospheric turbulence; the scintillation index remained below 0.019 under strong turbulence, confirming sub-THz link resilience. A theoretical comparison […] (P.1, L.26)

Introduction:

[…] The receiver utilizes direct conversion through an organic-plasmonic Mach-Zehnder modulator (MZM) and a dual-sideband receiver scheme that provides a SNR gain of up to 2 dB[33]. The receiver utilizes direct conversion through an organic-plasmonic Mach-Zehnder modulator (MZM). Compared to prior art, a dual-sideband receiver scheme that provides a SNR gain of up to 2 dB[33] has been employed. We report outdoor measurements of atmospheric turbulence effects on a high data rate sub-THz link, with scintillation indices staying below 0.019 even under strong turbulence. These results confirm the known resilience of sub-THz links to turbulence[34, 35] and, unlike many lab-based studies, provide valuable evidence to the community that sub-THz links perform reliably under real-world outdoor conditions over practical distances exceeding 1 km. For the first time outside laboratory conditions, we measured the impact of atmospheric turbulence on received sub-THz power, with the scintillation index remaining below 0.019 even in strong turbulence. […] (P.3, L.35)

Results:

[…] Sub-THz frequencies may offer a balance of high throughput and resilience towards adverse weather, making them an ideal complement to FSO to ensure high-capacity, reliable wireless communications.

The seamless transition from the optical domain to the sub-THz domain is enabled by the high-speed sub-THz down- und up-mixer as illustrated in Figure 1c. In case of adverse weather conditions, where FSO is blocked, the optical signal at the transmitter side can be re-routed to the sub-THz down-mixer and transmitted via a sub-THz carrier. On the receiver side, the signal is directly up-mixed back to the optical domain by means of a high-speed plasmonic modulator.

A high-speed sub-THz down- and up-mixer enables a seamless transition between the optical and sub-THz domains, as shown in Figure 1c. During adverse weather conditions that disrupt free-space optical (FSO) links, the optical signal at the transmitter can be rerouted through the sub-THz down-mixer and sent over a sub-THz carrier. At the receiver, the signal is directly up-mixed back into the optical domain using a high-speed plasmonic modulator. This seamless switching capability allows the optical and sub-THz links to complement each other in a confluent way, effectively addressing the limitations of each technology.

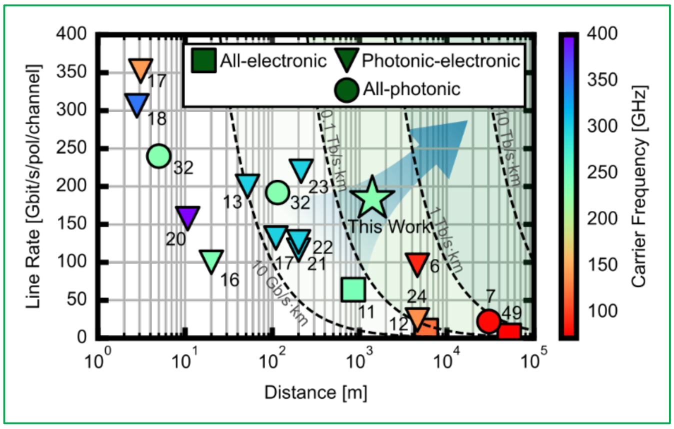

Fig. 2: State-of-the-Art THz-band (0.3-10 THz), sub-THz-band (100-300 GHz), E-band (60-90 GHz) and W-band (75-110 GHz) links. *Line rate per polarization and channel versus transmission distance of state-of-the-art wireless links. The color represents the carrier frequency used in the experiment.*

To show the potential of the all-photonic links, we plot in Figure 2 the transmission distance and achieved line rates for a selection of state-of-the-art all-electronic, photonic-electronic, and all-photonic link demonstrations. Further details to each demonstration are summarized in Table 2. This work shows that high data rate and long distance sub-THz links are indeed achievable by using photonic and plasmonic components. While raising the carrier frequency above 100 GHz brings the advantage of higher bandwidth, it raises a set of technological and operational challenges that need to be addressed. Firstly, we discuss the theoretical considerations for a long distance sub-THz link like atmospheric attenuation and turbulence, including a comparison to FSO communication. Secondly, we demonstrate in a field trial over 1400 m the sub-THz link demonstration (above 100 GHz) with the highest product of line-rate and distance to date, which is enabled by high-speed plasmonic modulators and the dual-sideband receiver. […] (P.5, L.9)

2

The title may cause confusion among readers. It is well established that atmospheric turbulence is generally not an issue for millimeter-wave (MMW) and THz signals but poses significant challenges for free-space optical (FSO) channels. The current title might mislead readers to believe that turbulence has become a problem for THz communication or that the paper solves the turbulence issue for FSO. Clarifying this or change the title is recommended.

We thank the reviewer and agree that the title might lead to confusion. We adjusted the title to stress the main achievement of the publication which is the product of distance and data rate.

Title:

~~Photonic-Plasmonic Sub-THz Wireless Link for 6G Demonstrating 160 Gbps over 1400 m in Strong Atmospheric Turbulence~~

All-Photonic Sub-THz Wireless Link Demonstrating 160 Gbps over 1400 m for 6G Radio Access Networks

3

While the authors compare THz and FSO performance under turbulent conditions, no clear technical contributions or innovations are evident in this comparison. Section III, "Influence of Atmospheric Turbulence on Wireless Link," merely reiterates a well-known result. Therefore, the innovation or contributions requires further clarification in this section.

We thank the reviewer for this remark. We agree that there are published studies on atmospheric turbulence and that it is a known result, that sub-THz is more resilient to atmospheric turbulence compared to FSO. To the best of our knowledge, all these studies use turbulence chambers to precisely control the conditions in a reproducible way. We believe that measurements of the effect of atmospheric turbulence in a field trial transmitting high data rates over kilometer distances, as one would encounter in a deployed link, are valuable to the community. More importantly, we believe that is necessary to verify that turbulences do not matter when both distance and modulation format and bit rates have been increased.

We adjusted the manuscript in the following way:

	Introduction:<br>[…] We report outdoor measurements of atmospheric turbulence effects on a high data rate sub-THz link, with scintillation indices staying below 0.019 even under strong turbulence. These results confirm the known resilience of sub-THz links to turbulence[34, 35] and, unlike many lab-based studies, provide valuable evidence to the community that sub-THz links perform reliably under real-world outdoor conditions over practical distances exceeding 1 km. For the first time outside laboratory conditions, we measured the impact of atmospheric turbulence on received sub-THz power, with the scintillation index remaining below 0.019 even in strong turbulence. The paper also compares FSO and sub-THz links, highlighting sub-THz's greater […] (P.3, L.40)
4<br>*The authors employ terms like All-E (all-electronic), P-E (photonic-electronic), and P-P (photonic-plasmonic) to describe system setups. For instance, P-E refers to a photonics-based transmitter (UTC-PD) and an electronics-based receiver. However, the term "photonic-photonic" would be more appropriate as it encompasses a broader range of solutions, whereas "photonic-plasmonic" excludes other photonic up (or down)-conversion methods, such as thin-film lithium niobate-based THz down-conversion.*<br>*Some of the latest experimental achievements in all-photonic up- and down-conversion solutions have not been included. Relevant references such as:*<br>*P. T. Dat et al., "151.5-GHz Sub-THz Signal Reception and Down-Conversion Using All-Optical Technology," 2024 Optical Fiber Communications Conference and Exhibition (OFC), San Diego, CA, USA, 2024, pp. 1–3.*	
	We thank the reviewer and agree that photonic-plasmonic excludes other technologies like TFLN. We adjusted the manuscript and replaced "photonic-plasmonic" with "all-photonic" throughout the manuscript. Furthermore, we added the reference the reviewer suggested and adjusted the introduction in the following way:
	Introduction:<br>[…] prevented a direct up-mixing of the sub-THz signals to the optical domain. Progress with the development of plasmonic modulators has brought up a modulator solution offering bandwidths in excess of 500 GHz[26] and most recently up to 1 THz[27] that in addition requires low driving voltages and small footprint[28]. Recently, direct up-mixing at 152 GHz was achieved by employing thin-film lithium niobate (TFLN) modulators, demonstrating 80 Gb/s back-to-back transmission[29]. While TFLN allow low optical insertion losses, the electro-optic bandwidth drops beyond 100 GHz due to the difficulty of phase matching the RF and optical wave. Plasmonic modulators on the other hand have brought up a modulator solution offering bandwidths in excess of 500 GHz[26] and most recently up to 1 THz[27] that in addition requires low driving voltages and small footprint[28]. Such plasmonic-based. Such plasmonic-based […] (P.3, L.17)
5<br>*Additionally, Table 2 only lists the distance-rate product for frequencies above 100 GHz, omitting notable demonstrations around 100 GHz in the E or W band (75–110 GHz). Including these would provide a more comprehensive comparison.*	
	We thank the reviewer for this comment and agree, that there are notable demonstrations in the E and W band which lie at the edge of the sub-THz regime.<br><br>We adjusted the manuscript in the following way:
	[…] Yet, FSO links can be strongly degraded by atmospheric turbulence effects and adverse weather, especially fog[4]. A solution offering sufficient bandwidth to transmit the high capacities signals as well as robustness against adverse weather and atmospheric turbulences[5] are wireless links operating at sub-THz above frequencies above 100 GHz. A solution offering sufficient bandwidth to transmit the high capacitiy signals as well as

robustness against adverse weather and atmospheric turbulences[5] are wireless links operating in the E/W (60 – 110 GHz) and in the sub-THz band (100 – 300 GHz). Demonstrations in the E/W band have achieved channel line rates of 96 Gbit/s over 4.6 km[6] and 32 Gbit/s over 28.8 km[7]. In case even higher data rates above 100 Gbit/s are required, sub-THz frequencies offer more bandwidths. Unlike fiber, these sub-THz links can be rapidly deployed permanently or on a temporary basis to enhance network capacity in specific areas during events such as emergency situations. These sub-THz links offer high data rates of >100 Gbit/s and unlike fiber, wireless links can be rapidly deployed permanently or on a temporary basis to enhance network capacity in specific areas during events such as emergency situations. Furthermore, in line-of-sight conditions, a direct […] (P.2, L.22)

Results:

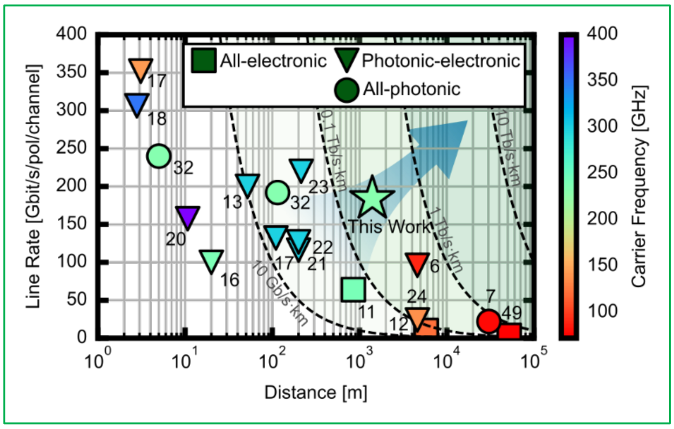

Fig. 2: State-of-the-Art THz-band (0.3-10 THz), sub-THz-band (100-300 GHz), E-band (60-90 GHz) and W-band (75-110 GHz) links. *Line rate per polarization and channel versus transmission distance of state-of-the-art wireless links. The color represents the carrier frequency used in the experiment.*

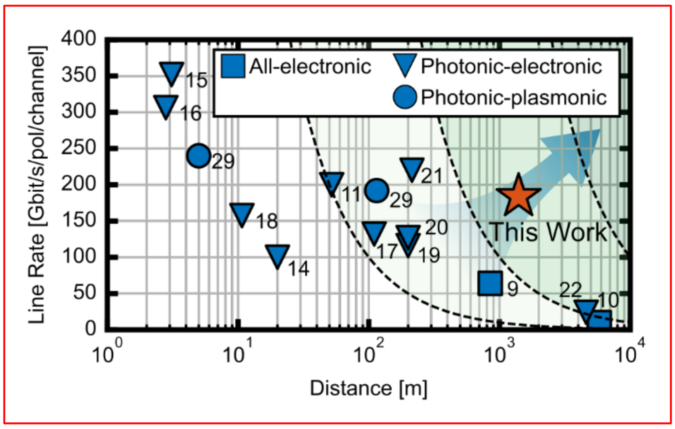

Fig. 6: State-of-the-Art Sub-THz links. *Line rate per polarization and channel versus distance of state-of-the-art sub-THz link carrier frequencies above 100 GHz.*

Tab. 2: State-of-the-Art sub-THz-band (100-300 GHz), E-band (60-90 GHz) and W-band (75-110 GHz) links sorted by distance-rate-product.

Tab. 2: State-of-the-Art sub-THz links with carrier frequencies above 100 GHz sorted by distance-rate-product.

Type[a]	Distance-rate-product [Gbit/s·km]	Line rate [Gbit/s/pol/channel]	Distance [m]	Carrier frequency [GHz]	BER (pre-FEC)	Technology TX/RX	Modulation Format	Year	Author	Reference
…	…	…	…	…	…	…	…	…	…	…

P-E	110.4	24	4600	135	8.6E-03 (Offline) (15% SD-FEC)	-	OFDM-16QAM	2022	Li et al.	24
E-E	187.2	3.6	52000	72	7E-02 (Offline)	E-Band Mixer/''	64QAM	2024	Guo et al.	49
P-E	441.6	96	4600	87.5	2.4E-02 (Offline) (15% SD-FEC)	PD/E-Band Mixer	PDM-16QAM	2023	Li et al.	6
P-P	857.6	32	26800	76.0	2.0E-02 (Offline) (15% SD-FEC)	PD/Phase Modulator	QPSK	2024	Liu et al.	7
P-P	257.6	184	1400	230	3.83E-02 (Offline)	UTC-PD/Plas. Modulator	QPSK, 8QAM, 16QAM	2024		This work

6

In the dual-band receiver, which factor has a greater impact on system performance: the insertion loss caused by the wavelength demultiplexer or the gain provided by combining two sidebands? How can these factors be balanced in the system design?

We thank the reviewer for this interesting question.

The quality of the signal depends on the SNR at the receiver. The total noise figure ($\mathrm{NF_{tot}} = 10\log(F_{\mathrm{tot}})$) of the receiver chain (low-noise EDFA 2 → DEMUX → EDFA 3 → Coherent RX) can be calculated using the Friis formula. In our case this yields

$$F_{\mathrm{tot}} = F_{\mathrm{EDFA2}} + \frac{L_{\mathrm{DEMUX}} F_{\mathrm{EDFA3}} - 1}{G_{\mathrm{EDFA2}}},$$

where F_{EDFA2} and G_{EDFA2} are the noise factor and gain of the low-noise EDFA, L_{DEMUX} the loss of the demultiplexer and polarization beam splitter and F_{EDFA3} the noise factor of the preamplifier EDFA 3. With the components used in our experiment, the total noise figure only increases by 0.2% with the additional 8 dB of loss of the demultiplexer, which is negligible. Therefore, in this regime, there is no trade-off between the demultiplexer loss and dual-sideband receiver gain.

To highlight, that there is a second EDFA (EDFA 3) used as a preamplifier for the coherent receiver, we added a subfigure d in Figure 5 showing the detailed schematic of the dual-sideband receiver. Furthermore, we added a theoretical link budget calculation in the supplementary information.

Results:

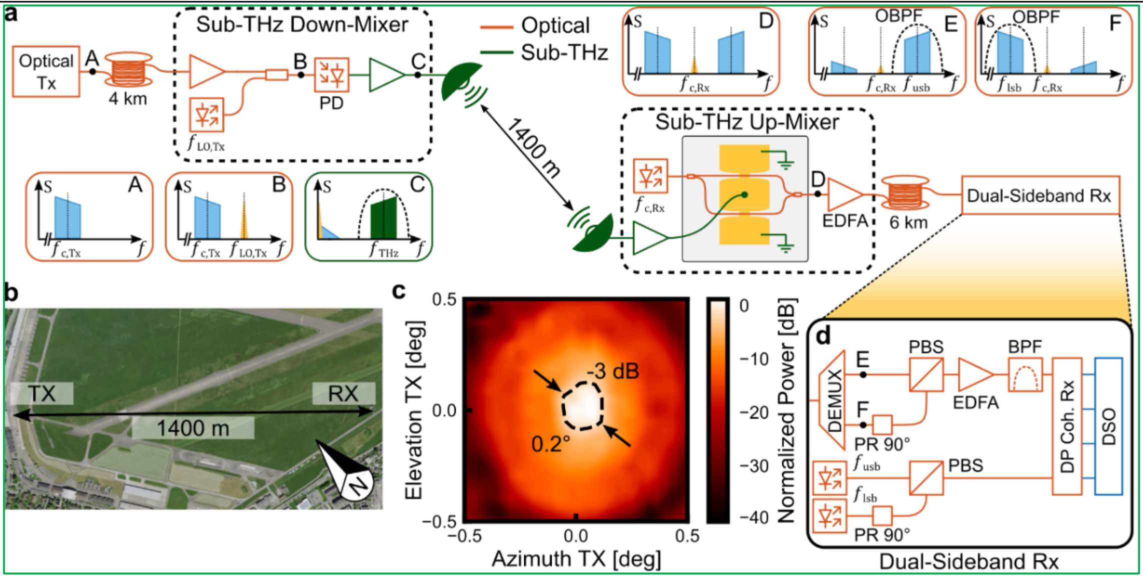

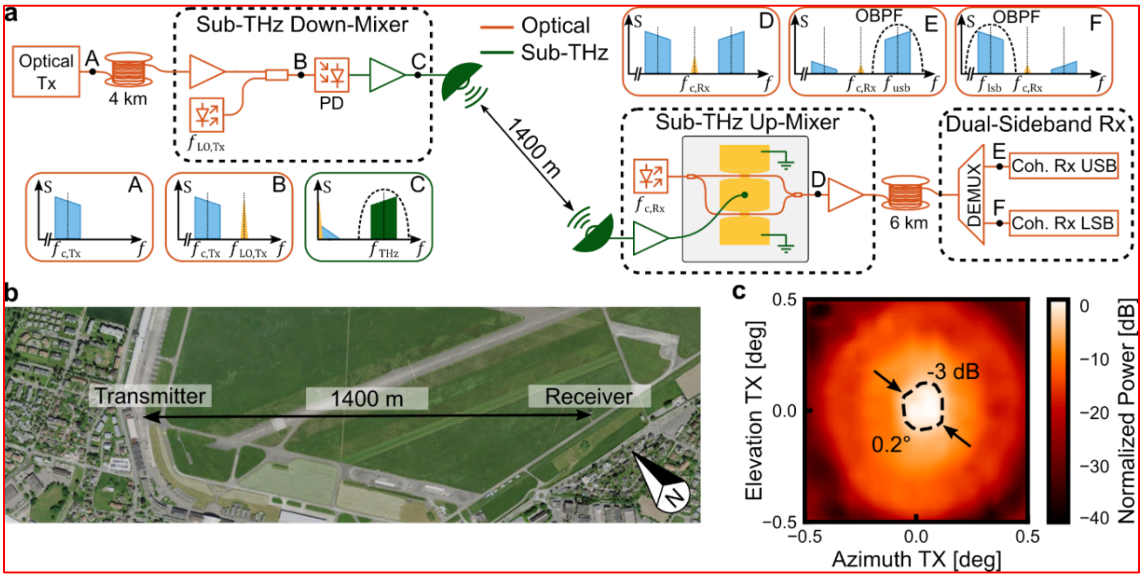

[…] **d** Implementation of the dual-sideband receiver which shifts one sideband to the orthogonal polarization and uses a commercial dual-polarization coherent receiver (DP Coh. Rx) and a digital sampling oscilloscope (DSO) to receive and sample the signals. […] (P.10, L.19)

[…] connection to the baseband unit (BBU). The dual-sideband receiver incorporates a wavelength demultiplexer to separate the two sidebands (point E and F). The polarization of the lower sideband (LSB) is rotated by 90° and is combined again with the upper sideband (USB) via a polarization beam splitter. The signal is amplified by a high-gain EDFA, bandpass filtered and processed by a dual-polarization heterodyne coherent receiver (DP Coh. Rx). This EDFA has a noise figure of 4.5 dB and is operated in power-controlled mode, which keeps the input power into the coherent receiver stable. Both sidebands are converted to baseband, where the electrical baseband signal is sampled by a high-speed digital sampling oscilloscope (DSO). The dual-sideband receiver incorporates a wavelength demultiplexer to separate the two sidebands (point E and F), which are then processed by two heterodyne coherent receivers (Coh. Rx), converting both sidebands to baseband, where the electrical baseband signal is sampled by a high-speed digital sampling oscilloscope (DSO). Digital signal processing […] (P.12, L.6)

Supplementary Information:

Supplementary Note 3: Link Budged

Supplementary Table 3 presents the theoretical link budget for each tributary. Signal power spectral density (PSD) is propagated through the channel using both measured and theoretical power spectra of the system components and the free-space link, see Supplementary Figure 2 and Figure 7e. Noise from sub-THz amplifiers is added to the signal and propagated through the channel. The SNR of the preamplifier coherent receiver is limited by the ASE-LO beat noise and is estimated using

$$\mathrm{SNR} = \frac{P_\mathrm{s}}{hf n_\mathrm{sp} B}, \tag{S10}$$

where P_s is the optical signal power, hf the photon energy, n_sp the SNR degradation from the shot-noise limit and B the bandwidth[5]. For high gain values, the SNR degradation of the amplifier chain is approximately $n_\mathrm{sp} \approx F_\mathrm{tot}/2$, where F_tot is the combined noise factor of the receiver chain, which consist of low-noise EDFA 2, loss of the demultiplexer and polarization beam splitter and preamplifier EDFA 3, see Figure 4d. According to the Friis formula, the total noise figure can be calculated by

$$F_\mathrm{tot} = F_\mathrm{EDFA2} + \frac{L_\mathrm{DEMUX} F_\mathrm{EDFA3} - 1}{G_\mathrm{EDFA2}}, \tag{S11}$$

where F_EDFA2 and G_EDFA2 are the noise factor and gain of the low-noise EDFA 2, L_DEMUX the loss of the demultiplexer and polarization beam splitter and F_EDFA3 the noise factor of the preamplifier EDFA 3. To estimate the SNR after the dual-sideband receiver, the ASE-LO beat noise power was reduced by 3 dB. The dominant noise sources were the sub-THz amplifier and the low-noise EDFA 2 at the receiver. The dual-sideband receiver's SNR gain matches the measured results well, showing that tributaries with lower optical sideband power are primarily limited by ASE-LO beat noise and benefit the most from the dual-sideband approach.

Supplementary Tab. 3: Theoretical link budget calculation for each tributary.

	Unit	Tributary							
Tributary Number		1	2	3	4	5	6	7	8
Bandwidth	GHz	8	8	8	8	8	8	8	8
Optical Launch Power (point A)	dBm	3.2	1.6	-1.2	2.3	-0.7	1.4	0.9	4.5
Optical Launch SNR (point A)	dB	17.2	19.7	21.3	23.1	21.9	20.9	18.6	16.2
Sub-THz Launch Power (point C)	dBm	2.5	4.7	4.3	2.7	-0.3	0.0	-0.3	5.1
Sub-THz Launch SNR (point C)	dB	17.1	19.6	21.1	22.8	21.4	20.6	18.4	16.2
Free-Space Path Loss	dB	-141.5	-141.7	-142	-142.3	-142.6	-142.8	-143.1	-143.3
Attenuation	dB	-5	-4.5	-4.5	-4.6	-4.8	-5	-5.3	-5.7
Antenna Gain	dB	111.7	111.7	111.7	111.7	111.7	111.7	111.7	111.7
Excess Loss	dB	7.0	7.0	7.0	7.0	7.0	7.0	7.0	7.0
Sub-THz Received Power	dBm	-39.2	-36.8	-37.5	-39.4	-42.9	-43.1	-44.1	-39.1
Sub-THz Power at Modulator	dBm	-14.1	-9.9	-11.1	-15.0	-21.8	-23.6	-22.9	-19.2
Optical Sideband Power (point D)	dBm	-44.5	-40.3	-41.5	-45.4	-52.1	-54	-53.2	-49.6
SNR Single Sideband (point E/F)	dB	12.3	15.8	15.6	12.7	6.2	4.4	5.0	8.3
SNR Dual-Sideband	dB	13.5	16.8	16.9	14.5	8.3	6.5	7.0	10.0

7

How were the atmospheric conditions' effects on sub-THz propagation experimentally verified? Specifically, under high humidity, were there significant signal attenuation changes? How does the system design compensate for these variations?

We thank the reviewer for this comment.

We tracked the humidity and temperature during the experiment and calculated the expected attenuation according to ITU-R models to be between 3 and 5 dB on the day of the measurement (2nd of June 2023). In our system, these changes are compensated by the gain of the preamplifier EDFA, which was operated in power-controlled mode, therefore adjusting the gain to reach the optimal power into the coherent receiver.

We adjusted the manuscript in the following way:

Results:

[…] EDFA 3 has a noise figure of 4.5 dB and is operated in power-controlled mode, which keeps the input power into the coherent receiver stable. Both sidebands are converted to baseband […] (P.12, L.11)

[…] The sub-THz signal is transmitted over a 1400 m free-space distance, 2 m above a predominantly meadow terrain with an asphalt runway in between, to the receiver. The channel loss comprises 142.5 dB of FSPL and, based on measured conditions (12.5 °C, 86% relative humidity), 5-7 dB of atmospheric attenuation, which is partially compensated by two identical […] (P.11, L.25)

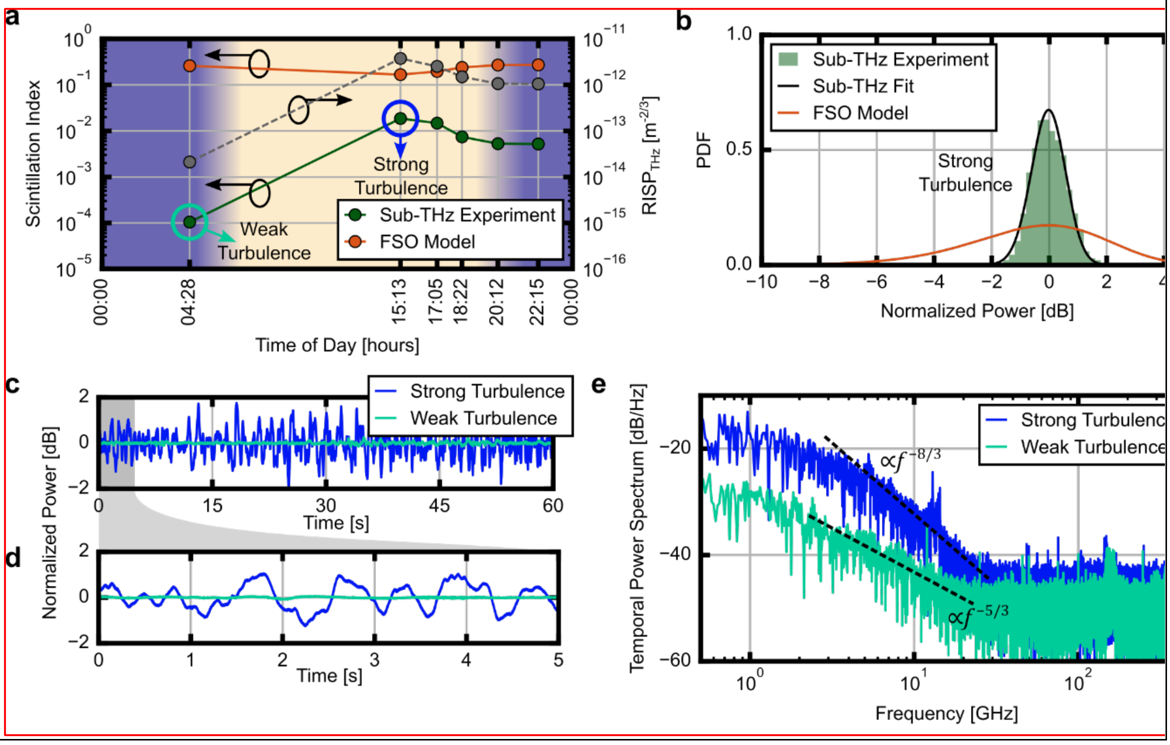

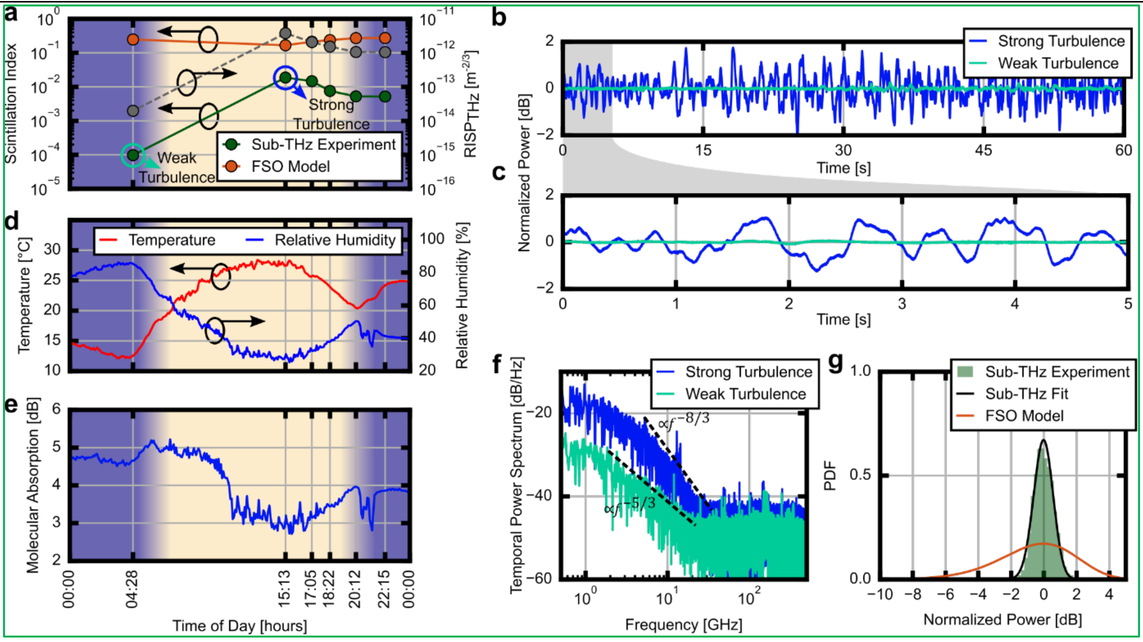

Fig. 7: Impact of atmospheric turbulence on sub-THz compared with FSO links. a *Scintillation index (SI) and refractive index structure parameter (RISP) of the sub-THz link measured over the course of the day taken on 2nd June 2023 compared with calculated SIs of the equivalent free-space optical (FSO) link for the same conditions. The time stamps indicate the times where scintillation measurements have been taken.* ***b*** *Recorded time traces of the normalized power for worst-case and best-case conditions,* ***c*** *time traces zoomed in to the first 5 seconds.* ***d*** *Measured temperature and humidity values at the transmitter and* ***e*** *estimated atmospheric attenuation according to ITU-R P.676.* ***f*** *Fourier transform of the measured normalized power bandpass filtered between 0.5 Hz and 500 kHz, and* ***g*** *Measured histogram of the normalized received power of the 1.4 km sub-THz link in worst-case turbulence conditions and fitted $\Gamma\Gamma$-model to extract a RISP of $3.92 \cdot 10^{-12}$ $m^{-2/3}$ (solid green line). The extracted RISP was used to calculate the expected probability density function (PDF) of a hypothetical 1.4 km FSO link operating at 1550 nm, showing much higher power fluctuations (solid red line).*

Fig. 7: Impact of atmospheric turbulence on sub-THz compared with FSO links. a *Scintillation index (SI) and refractive index structure parameter (RISP) of the sub-THz link measured over the course of the day taken on 2nd June 2023 compared with calculated SIs of the equivalent free-space optical (FSO) link for the same conditions.* ***b*** *Measured histogram of the normalized received power of the 1.4 km sub-THz link in worst-case turbulence conditions and fitted $\Gamma\Gamma$-model to extract a RISP of $3.92 \cdot 10^{-12}$ $m^{-2/3}$ (solid green line). The extracted RISP was used to calculate the expected probability density function (PDF) of a hypothetical 1.4 km FSO link operating at 1550 nm, showing much higher power fluctuations (solid red line).* ***c*** *Recorded time traces of the normalized power for worst-case and best-case conditions,* ***d*** *time traces zoomed in to the first 5 seconds, and* ***e*** *Fourier transform of the normalized power bandpass filtered between 0.5 Hz and 500 kHz.*

8

What are the bandwidth and operational frequency range of the UTC-PD and sub-THz amplifiers used in the system? Were the gain spectrum, frequency response, and attenuation characteristics of these components considered when selecting the central frequency? Is the chosen central frequency optimized for the system's best performance?

We thank the reviewer for this comment. Indeed, the carrier frequency was chosen to optimize the performance and is determined by the combined response of the UTC-PD and sub-THz amplifiers. We adjusted the manuscript in the following way:

Results:

[…] The carrier frequency of 226 GHz has been chosen to maximize the system's throughput and was limited by the combined bandwidth of the UTC-PD and the sub-THz amplifier. […] (P.11, L.21)

[…] amplifiers at these frequencies, see Supplementary Note 4. The dual-sideband […] (P.13, L.20)

Supplementary Information:

Supplementary Note 4: Sub-THz Components

Supplementary Figure 2 shows the normalized response and gain spectrum of the UTC-PD and sub-THz amplifiers used in the experiment. The high differences in output power across the band could be mitigated by splitting the band into 8 NFDM tributaries and perform power loading, see Supplementary Table 3. The optimal center frequency of 226 GHz was chosen to maximize the data rate through the channel.

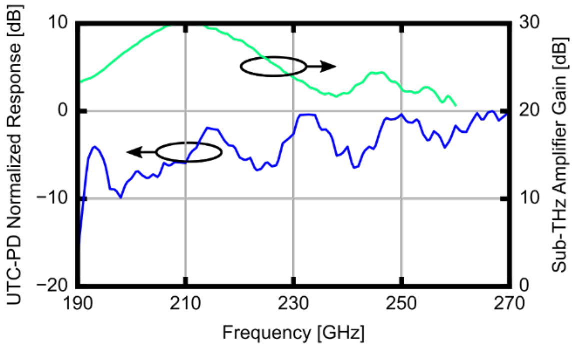

Supplementary Fig. 2: Normalized response of the sub-THz components. *The normalized response of the uni-travelling photodiode (UTC-PD) is shown in blue while the gain spectrum of the sub-THz amplifier is shown in green.*

9

The authors state, "since the free-space channel is linear, only linear equalizers were required." However, this is not true. Other system components could also introduce nonlinear distortions? There is no discussion of nonlinear processing in the DSP section. Given the use of high-gain sub-THz amplifiers, could nonlinearities be introduced in these components? If so, how are these nonlinear effects addressed or mitigated in the system?

We thank the reviewer very much for the comment. We totally agree with the reviewer that amplifiers can introduce severe non-linearities, especially when driven close to saturation. In our case we were below saturation and do not expect any severe non-linearities. This is underlined by two instances in the measured IQ diagrams.

- The symbols are equally spaced with gaussian noise which points away from non-linearities.
- Applying a Volterra algorithm with 1 second and 1 third-order tap does not improve the SNR again pointing away from non-linearities.

We added the following sentences to the manuscript to point this out more clearly.

Results:

[…] Combining the two baseband signals was achieved by utilizing a butterfly extension of the FFE. Despite the two sub-THz amplifiers in the link, non-linear equalization could not improve the SNR and BER as the amplifiers were not operated close to saturation. Therefore, non-linear equalization was omitted, reducing the computational complexity of the DSP. […] (P.13, L.9)

10

Deploying sub-THz links will require infrastructure such as transmitter/receiver installations, antenna alignment systems, and power supplies. What are the practical challenges and requirements for deploying these links in various environments? How can the process be streamlined to support large-scale rollouts for 6G networks?

We thank the reviewer for this comment.

For kilometer range point-to-point communication, high-gain antennas with low beam divergence and small half-power beam widths in the range of 0.2 degrees are required. One of the main challenges are:

- Aligning the antennas to this precision requires motorized alignment and, in our case, periodic realignment of the antennas.
- Protection of the antenna from the elements (wind, snow, ice) as contamination and movement will have higher penalties compared to lower frequencies.
- RF Packaging of the plasmonic devices to reduce the setup complexity.

We adjusted the manuscript in the following manner:

Discussion:

[…] systems, concluding that sub-THz offers high-capacity, low-complexity connectivity with enhanced robustness against adverse weather. The main challenges to overcome and allow for long-term deployment is the precise and periodic realignment of the antennas with 0.1 degrees precision and RF-packaging of the plasmonic modulator. Packaging techniques with only 0.8 dB of insertion loss over 70 GHz of bandwidth are available[53], which would improve the link budget and at the same time allow for a simple setup. Our transparent […] (P.17, L.33)

11

The paper uses RF probes for signal modulation, rather than packaged devices. What methods are planned for packaging these devices in the future? What bandwidth losses are anticipated in the packaging process, and are there specific techniques to minimize degradation while maintaining performance?

We thank the reviewer for this interesting question. RF packaging of the plasmonic modulator is necessary to make large scale deployment possible.

One packaging technique could be by coupling from a hollow metal waveguide to the chip by an on-chip antenna, as demonstrated in [1]. Between 200 and 270 GHz a loss of 0.8 dB could be expected. This is a lower loss compared to the loss of the probe we currently use, which is specified to be 2.3 dB. Therefore, packaging the device can potentially improve the performance, without significantly limiting the bandwidth.

A. Zamora, K. M. K. H. Leong, T. Reck, G. Chattopadhyay, and W. Deal, “A 170-280 GHz InP HEMT low noise amplifier,” *Int. Conf. Infrared, Millimeter, Terahertz Waves, IRMMW-THz*, pp. 1–2, 2014, doi: 10.1109/IRMMW-THz.2014.6956402.

We adjusted the manuscript in the following manner:

Discussion:

[…] systems, concluding that sub-THz offers high-capacity, low-complexity connectivity with enhanced robustness against adverse weather. The main challenges to overcome and allow for long-term deployment is the precise and periodic realignment of the antennas with 0.1 degrees precision and RF-packaging of the plasmonic modulator. Packaging techniques with only 0.8 dB of insertion loss over 70 GHz of bandwidth are available[53], which would improve the link budget and at the same time allow for a simple setup. Our transparent […] (P.17, L.33)

12

The paper describes two photoelectric conversions, and from Figure 4, it appears that the two optical signals at the UTC-PD are not phase-locked. Similarly, the optical signals at the

coherent receiver (Coh.Rx) also operate independently. This would result in poor phase noise in the electrical signals. Does the offline signal processing section address phase noise mitigation?

We agree with the reviewer, that the two free-running lasers at the transmitter and receiver introduce phase noise. Yes, the phase noise mitigation is done using blind phase search (BPS) phase recovery. We could not observe a SNR penalty.

We adjusted the manuscript in the following manner:

Results:
[…] carrier recovery, blind phase search (BPS) and a T/2-spaced feedforward equalizer (FFE). […] (P.11, L.36)

[…] Furthermore, phase noise introduced by the free-running lasers could be corrected by the BPS, without any significant penalty. The resulting baseband power spectral density is shown […] (P.13, L.5)

13
The demonstrated system has a low bandwidth utilization of the ultra-wideband MZM, and to improve the SNR, two coherent receivers were used. This trade-off in cost versus performance needs to be carefully analyzed. Could the system be scaled to higher THz center frequencies?

(1) With respect to the bandwidth utilization
We agree with the reviewer, that the full bandwidth of the MZM is not utilized. To date, the bandwidth of our system is primarily limited by the combined spectrum of the following components:

- The packaged UTC-PD
- Sub-THz amplifiers
- The WR-03 hollow metal waveguide of the packaged components.

More fundamentally, the bandwidth is additional limited by the atmospheric attenuation, see Figure 3. We could improve the bandwidth with future amplifier developments and by using more broadband high-speed PDs.

(2) With respect to the higher THz center frequencies
The system could be scaled to higher frequencies as the plasmonic modulators work up to 1 THz. However, for kilometer-range communication there is no value in going to higher THz frequencies due to the higher free space attenuation losses (see Figure 2a). Furthermore, current THz electrical amplifiers and source have less gain and power output at higher frequencies which would further limit the reach. However, due to the broadband nature of the modulator it is possible to switch operation to lower carrier frequencies for example to achieve better SNR in adverse weather conditions.

(3) With respect to the cost of the system
Regarding cost, we agree with the reviewer that the SNR gain of 2 dB per channel comes at a cost of doubling the amount of coherent receivers. The precise analysis of the cost-throughput trade-off in operation depends on many variables which are not available to us and would be beyond the scope of this paper. However, we added the sentence to the manuscript to note the problem:

[…] which is a 10 % increase in capacity compared to single sideband reception, which comes at the cost of one additional coherent receiver and wavelength demultiplexing. More precisely, the single-received LSB […] (P.13, L.32)

14
It is recommended to provide key THz device parameters, such as operating frequency and bandwidth, to enhance the clarity of the demonstration.

We thank the reviewer for the comment and added the response of the UTC-PD and gain of the sub-THz amplifier.

We adjusted the manuscript in the following way:

Results:

[…] amplifiers at these frequencies, see Supplementary Note 4. The dual-sideband […] (P.13, L.120)

Supplementary Information:

Supplementary Note 4: Sub-THz Components

Supplementary Figure 2 shows the normalized response and gain spectrum of the UTC-PD and sub-THz amplifiers used in the experiment. The high differences in output power across the band could be mitigated by splitting the band into 8 NFDM tributaries and perform power loading, see Supplementary Table 3. The optimal center frequency of 226 GHz was chosen to maximize the data rate through the channel.

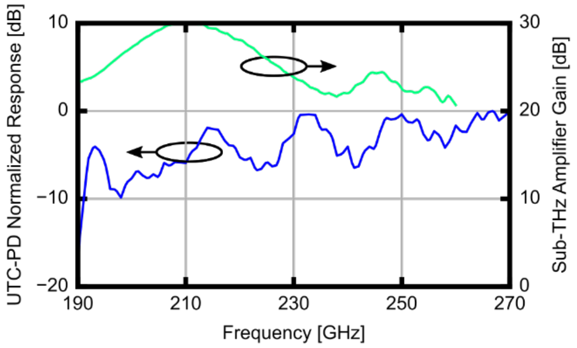

Supplementary Fig. 2: Normalized response of the sub-THz components. *The normalized response of the uni-travelling photodiode (UTC-PD) is shown in blue while the gain spectrum of the sub-THz amplifier is shown in green.*

15

Some typos: please check your definitions of up-mixer and down mixer. For example, the descriptions in Figure 4(a) are inconsistent with the subtitles. Should “Plasmonic Sub-THz Down-Mixer” be “Plasmonic Sub-THz Up-Mixer”?

We thank the reviewer for catching these typos. We corrected the one pointed out and re-checked the manuscript for any typos and inconsistency.

We thank the reviewer for all comments and interesting questions. We hope that the paper communicates the innovation and advancements over the state-of-the-art more clearly. We showed that the all-photonic approach using high-speed modulators (like plasmonic modulators) offers highest bandwidth and therefore data rate and can be used for kilometer ranged communication. The transition from isolated lab-based experiments to large-scale integrated field trials highlights both the potential and the challenges of this technology for future industrial applications.

Reviewer 3

1

The authors present a photonic-plasmonic sub-THz wireless link and achieve 160-Gbps transmission over 1400 m. This result is remarkable; however, it is similar to the work presented at OFC 2024. It would be beneficial if the authors could extend this research further. The authors have added discussions and comparisons with the FSO system; however, no solutions are proposed to overcome the limitations of each technology. Additionally, the two works presented in the manuscript are not closely related to each other. It would be better to separate them into two papers, each with extended results.

We thank the reviewer for his overall positive assessment are happy to hear that the reviewer finds the result remarkable.

(1) With respect to splitting the work into two papers:
We think that keeping the content in one paper helps to compare results and therefore suggest keeping it because we have to disagree that the two works are not closely related. Future communication systems will rely on fiber optics, FSO and wireless technologies. A potential solution to overcome the limitations of each technology is to employ both technologies complementary to each other as we tried to outline in Figure 1 b, c. The here demonstrated work shows how we can switch from the optical domain to the sub-THz domain and vice-versa while keeping the same established optical Rx and Tx.

We clarify this further in the main text with the following changes:

Results:

[…] The seamless transition from the optical domain to the sub-THz domain is enabled by the high-speed sub-THz down- und up-mixer as illustrated in Figure 1c. In case of adverse weather conditions, where FSO is blocked, the optical signal at the transmitter side can be re-routed to the sub-THz down-mixer and transmitted via a sub-THz carrier. On the receiver side, the signal is directly up-mixed back to the optical domain by means of a high-speed plasmonic modulator. […]
[…] A high-speed sub-THz down- and up-mixer enables a seamless transition between the optical and sub-THz domains, as shown in Figure 1c. During adverse weather conditions that disrupt free-space optical (FSO) links, the optical signal at the transmitter can be rerouted through the sub-THz down-mixer and sent over a sub-THz carrier. At the receiver, the signal is directly up-mixed back into the optical domain using a high-speed plasmonic modulator. This seamless switching capability allows the optical and sub-THz links to complement each other in a confluent way, effectively addressing the limitations of each technology. […] (P.5, L.21)

2

It is evident that RF systems, including those in the THz band, are robust to atmospheric turbulence due to the nature of signal reception. RF signals are received using large-diameter antennas. In contrast, FSO systems are more affected by atmospheric turbulence due to the use of very small-diameter photodetectors for signal detection. It is also well known that aperture averaging with a large-diameter lens can reduce the effects of turbulence. This effect is particularly significant when using direct fiber coupling at the receiver, as the incoming free-space signals are directly focused into a 10-µm core fiber. The turbulence can alter the angle of arrival of these signals. Given these factors, it is clear that the impact of turbulence in RF systems is minimal.

	We thank the reviewer for this remark. Indeed, the fact that the impact of atmospheric turbulences for RF systems is smaller compared to FSO is already known and has been demonstrated in laboratory based studies in turbulence chambers. We believe that measurements of the effect of atmospheric turbulence in a field trial transmitting high data rates over kilometer distances, as one would encounter in a deployed link, are valuable to the community.<br><br>We adjusted the abstract and introduction to stress this point in the following way:
	Abstract:<br><br>[…] We assessed power variations due to atmospheric turbulence; the scintillation index remained below 0.019 under strong turbulence, confirming sub-THz link resilience. A theoretical comparison […] (P.1, L.26)<br><br>Introduction:<br>[…] We report outdoor measurements of atmospheric turbulence effects on a high data rate sub-THz link, with scintillation indices staying below 0.019 even under strong turbulence. These results confirm the known resilience of sub-THz links to turbulence[34, 35] and, unlike many lab-based studies, provide valuable evidence to the community that sub-THz links perform reliably under real-world outdoor conditions over practical distances exceeding 1 km. For the first time outside laboratory conditions, we measured the impact of atmospheric turbulence on received sub-THz power, with the scintillation index remaining below 0.019 even in strong turbulence. […] (P.3, L.40)
3<br>*For the data shown in Fig. 7, why does the scintillation index for the FSO system remain unchanged during the measurement period, while the scintillation index for the THz system varies? What were the weather conditions and temperature at the time of the measurement? Typically, turbulence is stronger in hot weather, and it is usually most intense around noon. If the scintillation index (SI) of the FSO system does not change, it indicates that the atmospheric turbulence remained relatively constant during the measurement period. In that case, we cannot conclude that the experiment was conducted under strong turbulence conditions, as stated in the paper.*	
	We thank the reviewer for this interesting comment. Indeed, we measured the humidity and temperature during the day of the measurements and could observe that the measured scintillation index of the sub-THz link follows the trend very well, peaking in the afternoon, where temperature was highest.<br><br>Regarding the scintillation index of the FSO, we want to stress that these values are not measured but calculated according to the $\Gamma\Gamma$-model. The details of the calculation are written in the supplementary information. According to the $\Gamma\Gamma$-model as shown in Figure 4.a, the scintillation index can indeed become smaller even when RISP or distance are increased, or the wavelength is reduced. This matches with other publications such as:<br><br>W. Gao, C. Han, and Z. Chen, “Scintillation and Attenuation Modelling of Atmospheric Turbulence for Terahertz UAV Channels.”<br><br>In very strong turbulence, the optical wavefront becomes so distorted that spatial coherence is lost, leading to reduced interference effects and thus a decrease in scintillation.<br><br>We adjusted the manuscript in the following way:
	Results:<br>[…] to sub-THz link in strong turbulence. Interestingly, the SI of the theoretical FSO link is not proportional to the RISP values, indicating that the saturation regime is reached, which

can also be observed in Figure 4a. Figure 7b shows the probabilistic density function of the […] (P.17, L.19)

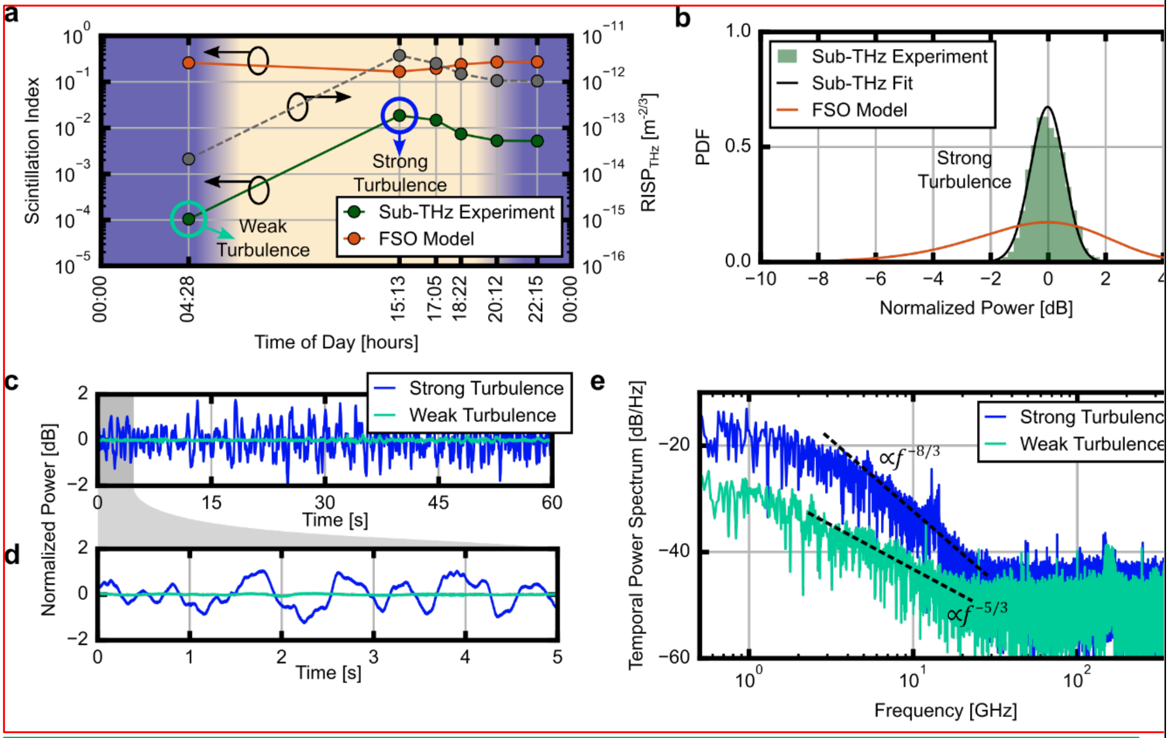

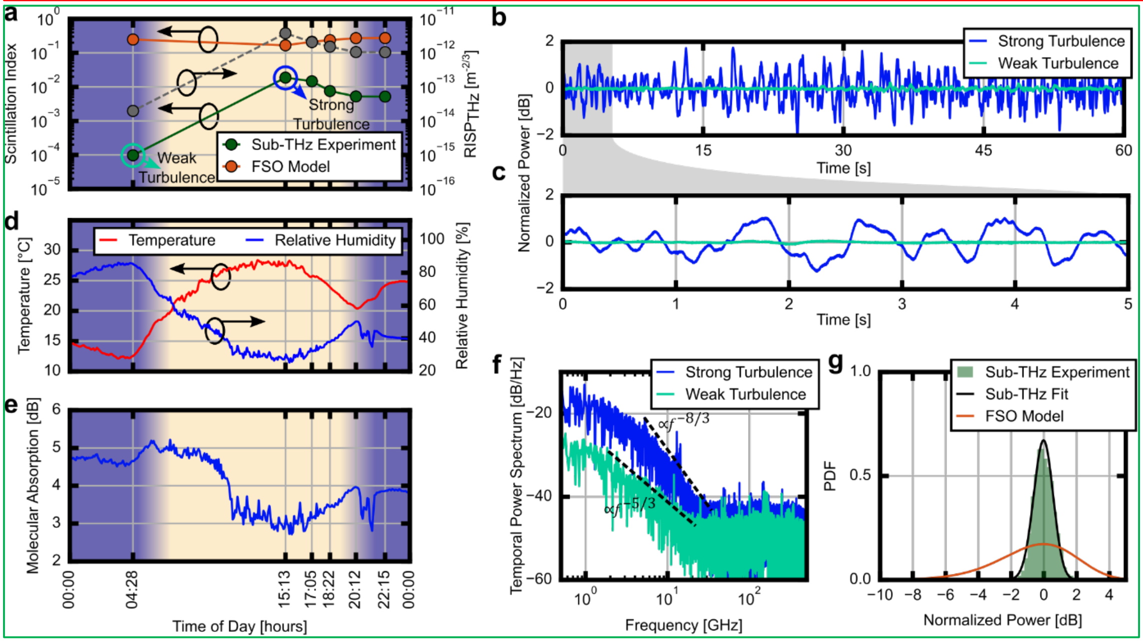

***Fig. 7: Impact of atmospheric turbulence on sub-THz compared with FSO links. a** Scintillation index (SI) and refractive index structure parameter (RISP) of the sub-THz link measured over the course of the day taken on 2nd June 2023 compared with calculated SIs of the equivalent free-space optical (FSO) link for the same conditions. The time stamps indicate the times where scintillation measurements have been taken. **b** Recorded time traces of the normalized power for worst-case and best-case conditions, **c** time traces zoomed in to the first 5 seconds. **d** Measured temperature and humidity values at the transmitter and **e** estimated atmospheric attenuation according to ITU-R P.676. **f** Fourier transform of the measured normalized power bandpass filtered between 0.5 Hz and 500 kHz, and **g** Measured histogram of the normalized received power of the 1.4 km sub-THz link in worst-case turbulence conditions and fitted $\Gamma\Gamma$-model to extract a RISP of $3.92 \cdot 10^{-12}$ m$^{-2/3}$ (solid green line). The extracted RISP was used to calculate the expected probability density function (PDF) of a hypothetical 1.4 km FSO link operating at 1550 nm, showing much higher power fluctuations (solid red line).*

***Fig. 7: Impact of atmospheric turbulence on sub-THz compared with FSO links. a** Scintillation index (SI) and refractive index structure parameter (RISP) of the sub-THz link measured over the course of the day taken on 2nd June 2023 compared with calculated SIs of the equivalent free-space optical (FSO) link for the same conditions. **b** Measured histogram of the normalized received power of the 1.4 km sub-THz link in worst-case turbulence conditions and fitted $\Gamma\Gamma$-model to extract a RISP of $3.92\cdot10^{-12}$ $m^{-2/3}$ (solid green line). The extracted RISP was used to calculate the expected probability density function (PDF) of a hypothetical 1.4 km FSO link operating at 1550 nm, showing much higher power fluctuations (solid red line). **c** Recorded time traces of the normalized power for worst-case and best-case conditions, **d** time traces zoomed in to the first 5 seconds, and **e** Fourier transform of the normalized power bandpass filtered between 0.5 Hz and 500 kHz.*

4

The change in the scintillation index (SI) of the THz system could be due to other factors, such as wind effects, power fluctuations in signal generation and transmission, and the overall stability of the systems. It is recommended that additional measurements be taken over multiple days and under varying weather conditions.

We agree with the reviewer, that it would be beneficial to measure the effects of turbulence and attenuation over multiple days in various weather conditions. Unfortunately, our time on the field was limited and we could only measure over the course of one day in sunny conditions.

To rule out effects due to power fluctuation in signal generation, transmission and overall stability, we have indeed performed short distance indoor experiments to confirm the stability of the system before going to the field trial. Some of these short distance results have been published by members of our group:

Horst Y*, et al.* Transparent Optical-THz-Optical Link at 240/192 Gbit/s Over 5/115 m Enabled by Plasmonics. *Journal of Lightwave Technology* **40**, 1690-1697 (2022).

Power fluctuations from wind-induced mechanical resonances, normally appear as narrowband peaks in the power spectrum. One strong peak at approximately 10 Hz is visible in our measurements (see Figure 7e.), which is in the range where we would expect some mechanical resonances. We couldn't observe any similar fluctuations in short distance experiments.

Furthermore, we attributed the fluctuations of the received power to scintillation due to following reasons:

- The temporal power spectrum follows the Kolmogorov power-law spectrum, which is expected for scintillation effects.
- The strength of the power fluctuations strongly depends on the time of day and the temperature and humidity, which we measured on site and have illustrated in the supplementary information. We observe high correlation between the temperature and measured scintillation index.

We adjusted the manuscript in the following way:

Results:

[…] instances during the day using a power meter with an averaging time of 200 μs. To confirm that the origin of the power fluctuations stem from the free-space channel, short range indoor experiments in the laboratory over 5 m have been conducted in prior studies[32], showing that the received power is stable. Therefore, the power fluctuations in this study are attributed to atmospheric turbulence effects. We use a $\Gamma\Gamma$-distribution and Andrew's method[47] to extract the […] (P15, L.8)

5

It is recommended that additional measurements be taken over multiple days and under varying weather conditions. Additionally, more data should be recorded and analyzed throughout a single day. A comparison of measurements taken over the same link would help reach a more precise conclusion.

	We thank the reviewer for his recommendation. While beyond the scope of this paper, it would indeed be preferable that future studies could collect more data over a longer period and in different weather conditions. Therefore, future work is planned where fiber, FSO and sub-THz are simultaneously deployed over longer periods of times. However, at the moment this is not possible due to lack of sub-THz packaged plasmonic modulator, which would allow for long-term measurements.<br><br>We adjusted the manuscript in the following way:
	Discussion:<br><br>[…] systems, concluding that sub-THz offers high-capacity, low-complexity connectivity with enhanced robustness against adverse weather. The main challenges to overcome and allow for long-term deployment is the precise and periodic realignment of the antennas with 0.1 degrees precision and RF-packaging of the plasmonic modulator. Packaging techniques with only 0.8 dB of insertion loss over 70 GHz of bandwidth are available[53], which would improve the link budget and at the same time allow for a simple setup. Our transparent […] (P.17, L.33)<br><br>[…] effects on a THz link, showing low scintillation index of 0.019 even under strong turbulence. The promising performance observed in this study highlights the need for future work involving long-term sub-THz link deployment and assessment of BER and EVM across diverse weather conditions. […] (P.17, L.43)

We thank the reviewer for the valuable feedback. We hope that with the paper can paint a more cohesive picture and relate the different technologies (fiber optics, FSO, sub-THz) to each other. We believe that combined FSO and sub-THz links leverage its respective advantages and disadvantages very well. We also believe that the all-photonic approach enables for a more confluent integration of both technologies. Furthermore, we plan to extend the research further to study long-term measurements in different weather conditions.

Eidgenössische Technische Hochschule Zürich
Swiss Federal Institute of Technology Zurich

Institute of Electromagnetic Fields (IEF)

ETH Zurich
M.Sc. Boris Vukovic
PhD Student
ETZ K 93
Gloriastrasse 35
8092 Zurich, Switzerland

Email: boris.vukovic@ief.ee.ethz.ch
Web: http://www.ief.ee.ethz.ch/

Zurich, 9 December 2025

Second Round of Revisions on Nature Communications Manuscript NCOMMS-24-85013

Dear Reviewers,

On behalf of all the co-authors I thank you for the supportive comments and thorough review, which enabled us to improve the quality of the paper. All your comments have been thoroughly addressed, and corresponding changes have been made to the manuscript. Below are detailed responses to each of the reviewer's comments (*italic gray*). Additionally, alterations made to the initial manuscript are clearly marked in red (~~removal~~) or green (addition) which are directly added below each comment.

Yours sincerely,

Boris Vukovic

Revisions Round 2

Reviewer 1

We thank the reviewer for his assessment and answer below the reviewer's comments below in detail to clarify these open points:

Authors added several details as requested. I think there are some errors in the text mixing dB and dBm. Authors should check them carefully. As an exemple, "9 dB" in line 322 should be 9 dBm I think.

We thank the reviewer for pointing out these errors and improving the quality of the paper. We checked the manuscript and supplementary again and have revised the manuscript in the following way:

Manuscript:

[…]. The sub-THz amplifiers at the down- and up-mixer have a saturated output power of 16 dBm and 1 dB compression point (P1dB) of 9 ~~dB~~ dBm. The carrier frequency of 226 GHz has been chosen to maximize the system's throughput and was limited by the combined bandwidth of the UTC-PD and the sub-THz amplifier. […] (P.9, L.32)

[…] modulator[29] (MZM) via a ground-signal-ground (GSG) RF probe with a characteristic impedance of 50 Ω. The total sub-THz power at the plasmonic MZM was -5.6 ~~dB~~ dBm. The electrodes of the MZM are modeled as a capacitive load, with an estimated capacitance of […] (P.10, L.6)

I am still a bit worried about the non-linear and noise analysis done, as authors used THz/sub-THz amplifiers. It is quite difficult here to assess the real contributions of these amplifiers, making comparisons somehow difficult. Can authors update this point?

We thank the reviewer for this remark and pointing us into looking into quantifying and compensating the non-linear distortion of the sub-THz amplifier. We will take this advise for future experiments. As for this work, we are not able to quantify the non-linear distortion. Therefore, we weaken the claim that we don't have non-linear distortion in our link.

Regarding the noise analysis, we updated the supplementary material with an additional figure, showing the signal and noise power spectral density of the sub-THz amplifiers and EDFA.

We updated the manuscript and supplementary section in the following way:

Manuscript:

[…] Combining the two baseband signals was achieved by utilizing a butterfly extension of the FFE. Despite the expected non-linear distortion of the two sub-THz amplifiers in the link, non-linear equalization was omitted, reducing the computational complexity of the DSP. ~~Despite the two sub-THz amplifiers in the link, non-linear equalization could not improve the SNR and BER as the amplifiers were not operated close to saturation. Therefore, non-linear equalization was omitted, reducing the computational complexity of the DSP.~~ Furthermore, phase noise introduced by the free-running lasers could be corrected by the BPS, without any significant penalty. […] (P.11, L.18)

Supplementary:

[…] Noise from sub-THz amplifiers is added to the signal and propagated through the channel. The noise figure of the sub-THz amplifier is assumed to be 20 dB. The SNR of the preamplifier […] (P.7, L.7)

[…] preamplifier EDFA 3. The noise figure of EDFA2 is 3.7 dB. To estimate the SNR after the dual-sideband receiver, the ASE-LO beat noise power was reduced by 3 dB. Supplementary Figure 4 shows the power spectral density of the signal after the plasmonic modulator (see point D in Fig. 3) and the contributing noise sources. The dominant noise sources were the sub-THz […] (P.7, L.17)

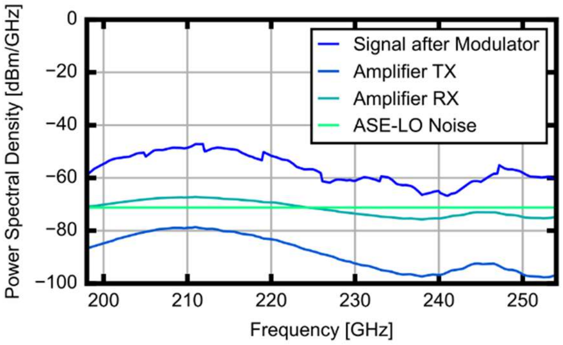

Supplementary Fig. 4: Calculated power spectral density of the signal and different noise sources. *The dominant noise sources are identified to be the sub-THz amplifier at the sub-THz receiver (Amplifier RX) and the ASE-LO beat noise.*

Reviewer 2

The authors have revised the manuscript as requested, and the overall quality has improved. However, upon careful re-reading, several issues remain that need to be addressed before the paper meets the rigorous standards of Nature Communications.

We are happy to hear, that the overall quality has improved and thank the reviewer for pointing out further issues, which we address below point by point. We hope that these changes improve the quality to the standards of Nature Communications.

1. As noted by all reviewers, the manuscript title has been revised as requested. However, I strongly recommend removing numerical values from the title to prioritize emphasis on the innovative concepts—particularly since the reported distance is not exceptional when compared to other published works.

We thank the reviewer for this remark.

Although the distance is not exceptionally high compared to other work, we want to emphasize that the combination of distance and data rate for this carrier frequency range (100-300 GHz) is a record. Furthermore, we argue that in the scenario of 6G radio access networks (RANs), going to higher data rates is more desirable compared to going to higher distances, as the remote radio units (RRUs) will be closer together as the reach of higher carrier frequencies gets shorter.

We follow the recommendation of the reviewer and removed numerical values from the title:

Plasmonic Modulator Enabling Kilometer-Range, High-Throughput Sub-THz Links for 6G Networks

~~All-Photonic Sub-THz Wireless Link Demonstrating 160 Gbit/s over 1400 m for 6G Radio Access Networks~~

2. Regarding new measurements of environmental parameters along the channel, the authors report the 24-hour variations of temperature and relative humidity at the transmitter. For a long-distance transmission experiment, environmental conditions along the propagation path are equally important; segment-wise measurements along the link would allow a more precise attribution of environmental effects. In this experiment, did the authors also measure the relevant parameters at the receiver? Are the variations consistent with those at the transmitter, and do they show consistent trends in their impact on the link?

We thank the reviewer for this comment and agree that multiple measurement points along the path would be ideal. Unfortunately, it was not possible to place more measurement stations as the link crossed an active runway. However, we measured the temperature and humidity at the transmitter and the receiver, and we added both measurement values to Figure 5d. The temperature and humidity values between transmitter and receiver match well with each other.

The variation between TX and RX can be explained by the terrain beneath the measurement station: The transmitter station was placed over concrete while the receiver station was placed above the meadow.

We added the measurements of TX and RX to Figure 5d and therefore adjusted the manuscript in the following way:

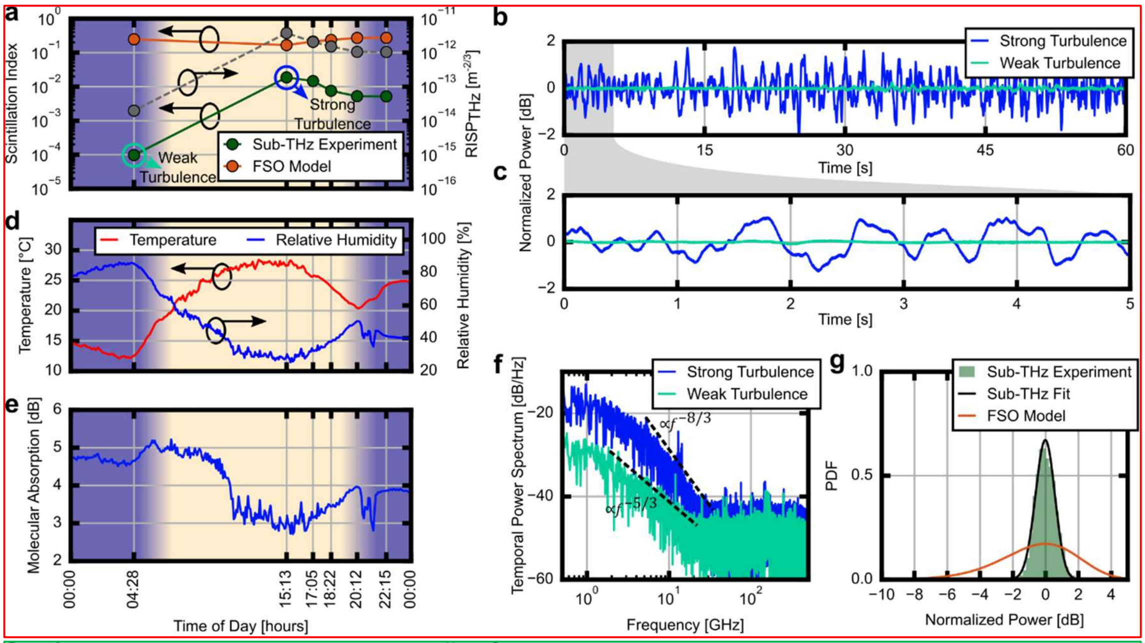

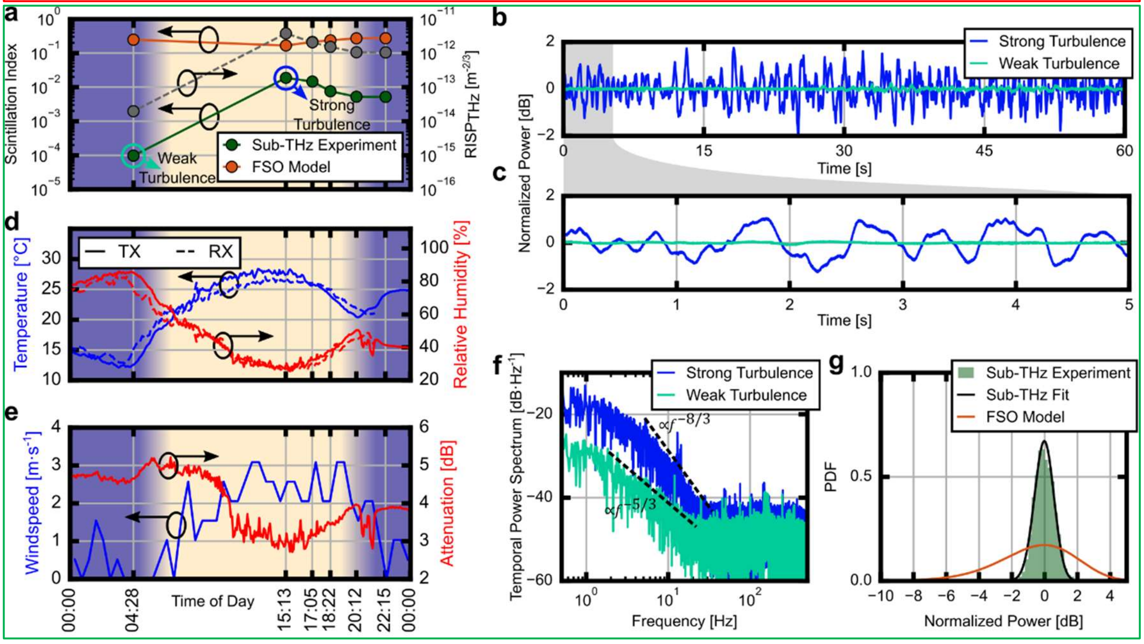

[…] Measured temperature and humidity values at the transmitter (solid line) and receiver (dashed line). ~~and~~ **e** ~~estimated~~ Estimated atmospheric attenuation according to ITU-R P.676[38]. **f** Fourier transform of the measured normalized power bandpass filtered between 0.5 Hz and 500 kHz, and **g** Measured histogram of the normalized received power […]

3. For the turbulence strength, the authors compute it from the power fluctuations of the optical signal modulated by the received THz signal. While such fluctuations may partially reflect variations in the received THz power, how accurate is this power-based approach for evaluating both the THz and FSO links? How does it compare with directly measuring power using a THz power meter at the THz receiving antenna?

We thank the reviewer for this interesting remark. While outside of the scope of this second round of revisions, we will still answer the question.

While we expect the absolute power level calibration to be off by a few dB, we only need the relative power changes over a period of 60 seconds for our analysis which we can

measure reliably. The relative power accuracy depends on the measurement time (60 seconds) and multiple components in our setup, which are:

- Relative laser power stability: ± 0.01 dB
- Stability of V_π of the modulator: (no fluctuations observed)
- Stability of insertion loss of the modulator: (no fluctuations observed)
- Optical power meter drift: ± 0.02 dB
- Fiber-to-chip coupling stability: (not observed during 60 s).

The relative power stability of both the laser and optical power meter, as specified in their datasheets, is approximately ±0.03 dB. Drift of the halfwave-voltage and insertion loss of the plasmonic modulator are not observed. To ensure constant fiber-to-chip coupling, we monitored the carrier power at the output of the modulator. No fluctuations were measured over 60 seconds.

We made the following clarification to the main text:

Manuscript:
[…] To quantify the effect of atmospheric turbulence on the sub-THz link, the optical power of a single sideband at the receiver at point D was recorded for 60 seconds at 6 different time instances during the day using a power meter with an averaging time of 200 μs. The relative sub-THz power fluctuations were derived from the optical sideband measurements with an estimated accuracy of ±0.05 dB. To confirm that the origin of the power fluctuations stem from the free-space channel, short range indoor experiments in the laboratory over 5 m have been […] (P.14, L.7)

4. The authors state that they have replaced "photonic-plasmonic" with "all-photonic" throughout the manuscript. However, footnote "a" below Table 2 still annotates "P–P" as "photonic-plasmonic." Please carefully check the entire manuscript for similar instances and ensure consistency. Furthermore, the types of all-electronic systems should be consistent (e.g., using "All-E" throughout instead of a mix of "All-E" and "E-E") to prevent confusion.

We thank the reviewer for catching this mistake. We checked the manuscript for this mistake and corrected it.

We changed the manuscript in the following way:

Type[a]	Line-rate-distance product	Line rate [Gbit/s/pol/	Distance [m]	Carrier frequency [GHz]	BER (pre-FEC)	Technology TX/RX	Modulation Format	Year	Author	Reference
…	…	…	…	…	…	…	…	…	…	…
All-E~~E-E~~	187.2	3.6	52000	72	7E-02 (Offline)	E-Band Mixer/"	64QAM	2024	Guo et al.	49
P-E	441.6	96	4600	87.5	2.4E-02 (Offline) (15% SD-FEC)	PD/E-Band Mixer	PDM-16QAM	2023	Li et al.	6
All-P~~P-P~~	857.6	32	26800	76.0	2.0E-02 (Offline) (15% SD-FEC)	PD/Phase Modulator	QPSK	2024	Liu et al.	7
All-P~~P-P~~	257.6	184	1400	230	3.83E-02 (Offline)	UTC-PD/Plas. Modulator	QPSK, 8QAM, 16QAM	2024		This work

[a]The type of the link is indicated by All-E (all-electronic), P-E (photonic-electronic), and All-~~PP-P (photonic-plasmonic)~~ (all-photonic). […]

The authors have added the BER and FEC overhead from previous works in Table 2 but have not specified the BER threshold and corresponding overhead used in this paper. It is unfair to only compare the line rate or line rate-distance product, with very different BER thresholds. The author should give clear net-data rate after the FEC corrections. In addition, the BER results in Figure 6(b) appear not to meet common pre-FEC BER thresholds (e.g., 1e-2 or 2e-2). The authors must explicitly state what kind of BER overhead used to justify the achieved line rate and net data rate, ensuring the claim is convincing.

We thank the reviewer for pointing this out.

We added the net rate, FEC type, FEC overhead, BER threshold to Table 2 and changed the figure-of-merit to net-rate-distance-product. Furthermore, we also replaced the y-axis Figure 2 with the net rate.

To select the appropriate FEC of our link, we used the normalized general mutual information (NGMI), as it is a more reliable predictor for post-FEC BER compared to pre-FEC BER[1]. As we would apply FEC before we split the bits to the different tributaries, we first calculate the NGMI of the whole channel according to

$$\mathrm{NGMI}=\frac{\sum_{j=1}^{N}\mathrm{GMI}_j}{\sum_{j=1}^{N}m_j},$$

where N is the number of tributaries, GMI_j is the general mutual information (GMI) of each tributary, and m_j are the bits per symbol of each tributary. In our case, the total NGMI aggregates to 0.8613. According to this publication[2], we can use SD + HD concatenated FEC with a NGMI threshold of 0.8574. This FEC would give an overall code rate of 0.8098 which corresponds to an SD-FEC overhead of 20%. Therefore, the achievable information rate (AIR) of our link is 158.48 Gbit/s while the net rate with 20% overhead is 153.3 Gbit/s.

1. Alvarado, A., Agrell, E., Lavery, D., Maher, R. & Bayvel, P. Replacing the Soft-Decision FEC Limit Paradigm in the Design of Optical Communication Systems. *J. Light. Technol.* **33**, 4338–4352 (2015).
2. Hu, Q. *et al.* Ultrahigh-Net-Bitrate 363 Gbit/s PAM-8 and 279 Gbit/s Polybinary Optical Transmission Using Plasmonic Mach-Zehnder Modulator. *J. Light. Technol.* **40**, 3338–3346 (2022).

We added the references and changed the manuscript in the following way, to make the claim more convincing:

Manuscript:

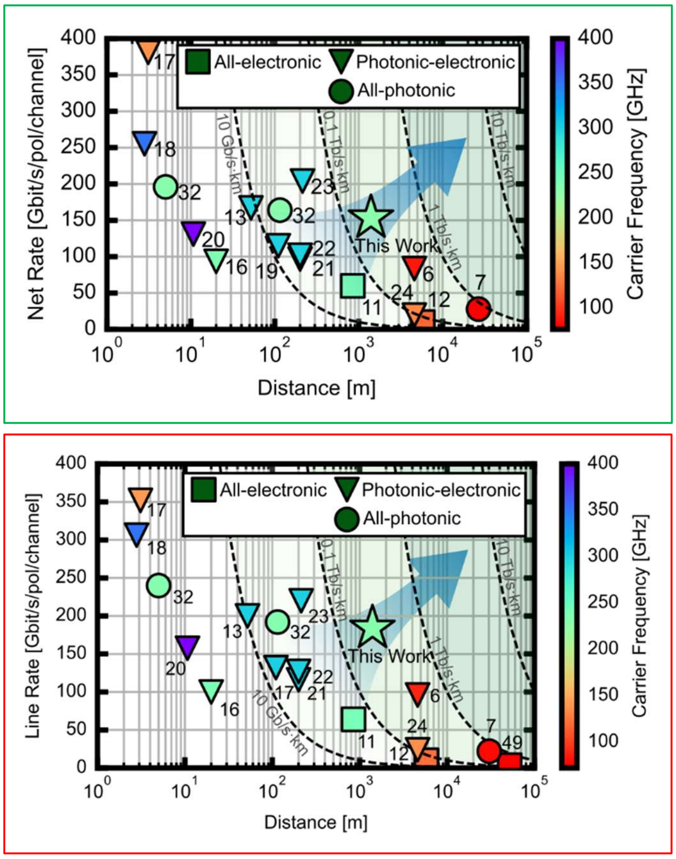

Fig. 2: State-of-the-Art THz-band (0.3-10 THz), sub-THz-band (100-300 GHz), E-band (60-90 GHz) and W-band (75-110 GHz) links. *~~Line~~ Net rate per polarization and channel versus transmission distance of state-of-the-art wireless links. The color represents the carrier frequency used in the experiment.*

[…] Figure 4b presents the constellation diagrams, the bit-error rate (BER), and general mutual information (GMI) of each tributary. These measurements are performed in best case conditions, where scintillation was at a minimum. Since forward error correction (FEC) is applied prior to bit demultiplexing, the overall GMI is obtained by averaging the values across all tributaries. This results in an average GMI of 2.48 bit/symbol. Multiplying this value by the 64-GBaud symbol rate yields an achievable information rate (AIR) of 158.7 Gbit/s. To determine the appropriate FEC scheme, we evaluate the normalized GMI (NGMI), which is a more reliable predictor of post-FEC performance compared to the pre-FEC BER[49]. Normalizing the total GMI by the average bits per symbol gives an NGMI of 0.8613. Using a concatenated SD+HD FEC with a 0.8574 NGMI threshold and 20% overhead[50] results in a net information rate of 153 Gbit/s. For comparison, we also evaluate the system performance when only the lower sideband (LSB) is detected. In this single-sideband configuration, the average GMI is 2.25 bit/symbol, corresponding to an AIR of 144.0 Gbit/s. Including the upper sideband (USB) in a dual-sideband reception scheme increases the GMI to 2.48 bit/symbol and the AIR to 158.7 Gbit/s. This represents an improvement of approximately 10 percent. The gain requires an additional coherent receiver and wavelength-demultiplexing stage, but it provides a substantial increase in spectral efficiency. […] (P.12, L.1)

[…] ~~In Figure 6b, we show the constellation diagram, the bit-error rate (BER) and general mutual information (GMI) of each tributary. These measurements are performed in best case conditions, where scintillation was at a minimum. The achievable information rate (AIR) is calculated by multiplying the baud rate of 64 GBaud by the~~

	~~GMI averaged over all tributaries. By employing dual-sideband reception an AIR of 158.7 Gbit/s is achieved, which is a 10 % increase in capacity compared to single sideband reception, which comes at the cost of one additional coherent receiver and wavelength demultiplexing. More precisely, the single-received LSB signal achieved an average GMI of 2.25 bit/symbol, equating to an AIR of 144.0 Gbit/s. When the USB signal was included in the dual-sideband reception scheme, the GMI increased to 2.48 bit/symbol, corresponding to an AIR of 158.7 Gbit/s.~~ […]

Reviewer 3

The authors have made efforts in responding to comments and revising the manuscript. However, the paper remains unclear. It is difficult to determine the main focus—whether it is on high-capacity, long-distance transmission, or on a comparison with the FSO link.

We thank the reviewer for this remark.

We changed the title to clarify that the main focus is on high-capacity, long-distance transmission and not on the comparison with FSO.

We do this by changing the title and moving the section "Comparison of Sub-THz and FSO Communications" to the supplementary material, as suggested by the reviewer.

Plasmonic Modulator Enabling Kilometer-Range, High-Throughput Sub-THz Links for 6G Networks

~~All-Photonic Sub-THz Wireless Link Demonstrating 160 Gbit/s over 1400 m for 6G Radio Access Networks~~

While several results and discussions on the effects of atmospheric turbulence on THz and FSO links are presented, along with their comparison, these appear rather trivial and not significant enough to be included in a main Nature Communications paper.

The reviewer calls these results trivial. We do not agree with this statement and think that it is necessary to monitor and understand the behavior of such a link in a real-world scenario. We can hardly cite other systems that used an atmospheric chamber

Moreover, the revised title does not fully reflect the scope of the work. The authors are advised to substantially reorganize the paper, focusing on high-data-rate and long-distance transmission.

We thank the reviewer for the comment. We revised the title in the following way, highlighting the high-data-rate and long-distance.

Plasmonic Modulator Enabling Kilometer-Range, High-Throughput Sub-THz Links for 6G Networks

~~All-Photonic Sub-THz Wireless Link Demonstrating 160 Gbit/s over 1400 m for 6G Radio Access Networks~~

However, more details and extensions should be provided compared to their previous work, including the OFC 2024 paper [36] and the Optics Express paper [33]. The influence of atmospheric turbulence on the THz link can then be included to highlight its advantages over the FSO link.

We respectfully believe that this comment goes beyond the scope of a second-round revision. The request for additional details and extensions is rather general and not linked to specific aspects of the manuscript. Furthermore, we do not feel that adding such extensions would improve the clarity of the paper, which we understand to be the reviewer's primary concern.

I suggest that the discussion and theoretical results currently presented in the "Comparison of Sub-THz and FSO Communications" section be moved to the Supplementary Information.

We thank the reviewer for the feedback and apologize that the main focus is still not communicated clearly enough.

	To make the main claim clearer, we moved the section "Comparison of Sub-THz and FSO Communications" to the supplementary material, as suggested by the reviewer.
	Moved section "Comparison of Sub-THz and FSO Communications to supplementary material.
1. In Fig. 7(a), is there an underlying physical explanation for why the scintillation index of the THz link does not saturate in the same way as the FSO link?	
	We thank the reviewer for this question. Indeed, as the wavelength of the sub-THz link is approximately 1000 times larger compared to infrared. Therefore, keeping the conditions the same, the sub-THz wave requires a 1000 times higher propagation length to acquire the same phase difference as an IR beam would. To reach the saturation regime for the sub-THz regime, one would need to increase the propagation distance.<br><br>This is already detailed in reference[1] which we cite at the corresponding place.<br><br>1. Gao, W., Han, C. & Chen, Z. Scintillation and Attenuation Modelling of Atmospheric Turbulence for Terahertz UAV Channels.
	**Supplementary:**<br>[…] Notably, sub-THz links exhibit a reduction in turbulence-induced effects by more than two orders of magnitude compared to FSO links. This difference arises from the fact that the wavelength of sub-THz waves is roughly three orders of magnitude larger than that of FSO waves. Scintillation is caused by refractive-index inhomogeneities in the atmosphere, which act as random phase-distorting elements. To accumulate the same level of phase distortion, a sub-THz beam would need to propagate over distances that are orders of magnitude longer than those required for an FSO beam[1]. Consequently, sub-THz links are significantly less susceptible to atmospheric turbulence. […] (P.2, L.32)
2. The authors state that a short-link experiment was previously conducted to confirm that no power fading is caused by the system itself. However, system stability depends heavily on operational conditions—such as cable placement, temperature, and vibrations. In particular, for field operations, the conditions differ from those of short-link experiments conducted indoors. Given these factors, it is difficult to conclude that the system is completely stable without any power fading. Therefore, results under different turbulence conditions should be analyzed and compared to confirm the actual effect of turbulence on the THz links.	
	We measured the power fading at different times during the day, as illustrated in Fig. 5a, 5b, 5c. We clearly see a dependence between strong and weak turbulence conditions which were all measured at the same setup.<br><br>Regarding the points:<br>• Cable placement: We do not see how placing the cables changes the performance of the system. For the strong and weak turbulence conditions outlined in the figure, the cables have not been changed.<br>• Temperature: The container where the setup was placed was AC controlled. Therefore, we expect only minor temperature fluctuations at the setup.<br>• Vibrations: We used the same optical table that we used for the indoor 115m experiments. As the optical table is isolating exactly in the frequency range that we plot in Fig. 5f, we do not believe that vibration changes during the day would lead to our measurement results.

3. For the THz link, wind can have a significant impact, potentially causing power fading due to misalignment of directive antennas. While the authors present temperature and humidity data, measured wind speed at the time of measurement should also be included and discussed.

We thank the reviewer for this comment, and we agree that wind can cause movement and misalignment of the antennas. Unfortunately, at the time of measurement we didn't have equipment to measure the wind speed directly. In future experiments, wind speed sensors should be installed to measure this effect. However, we included wind measurements from a nearby weather station. Indeed, the wind speed also correlates with the strength of the fluctuations. To mitigate the effect of the wind-induced pointing-error, we placed the whole setup including the antennas inside an office container. However, we cannot completely exclude wind-induced pointing errors.

We acknowledge this point and adjust our manuscript in the following way:

Manuscript:

[…] One can see that the temporal power spectrum of scintillation decreases with frequency with a power law according to $\propto f^{-8/3}$ and $\propto f^{-5/3}$ and plateaus beyond 30 Hz, which is in good agreement with the Kolmogorov power-law spectrum[52]. However, it should be noted that the SI and therefore RISP in this study is likely overestimated, as the observed power fluctuations are possibly driven by a combination of atmospheric turbulence and wind-induced pointing errors. Although the antennas were located indoors, wind effects cannot be entirely excluded. Figure 5d shows the measured temperature and relative humidity at the transmitter and receiver, while Figure 5e presents the calculated atmospheric attenuation and measured average wind speed at the airport. Scintillation levels are highest in the afternoon, coinciding with peak temperature, but also increase when wind speeds are strongest, as shown in Figure 5e. This suggests that scintillation is the dominant factor, though some contribution from wind effects remains possible. […] (P.14, L.33)

[…] Therefore, the power fluctuations in this study are attributed to atmospheric turbulence and wind-induced pointing error effects. We use a $\Gamma\Gamma$-distribution and Andrew's […] (P.14, L.18)

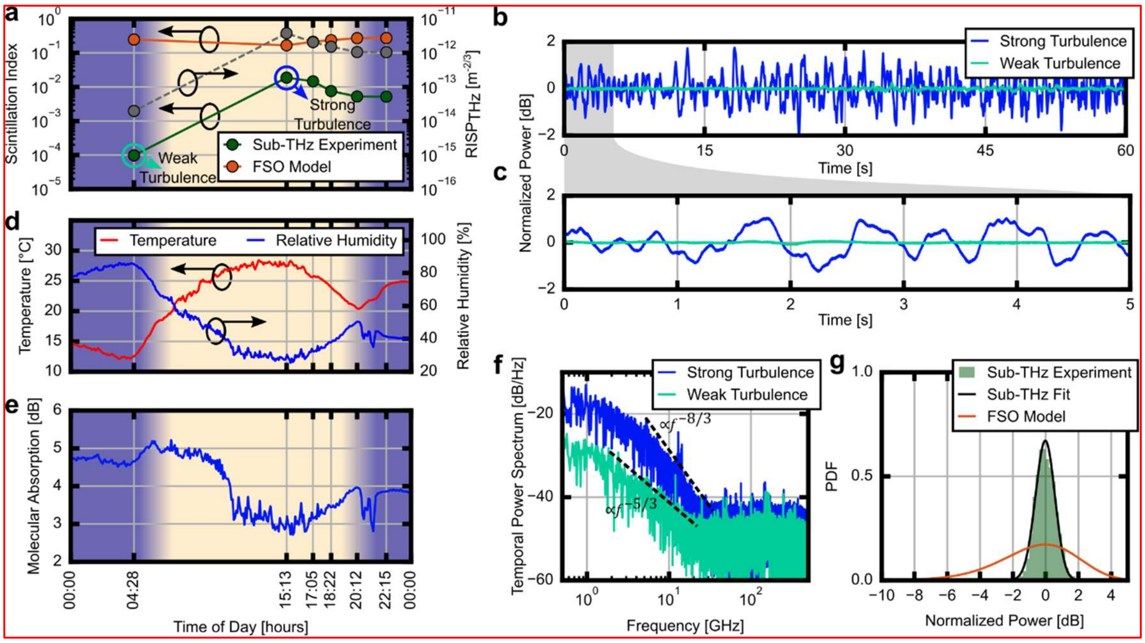

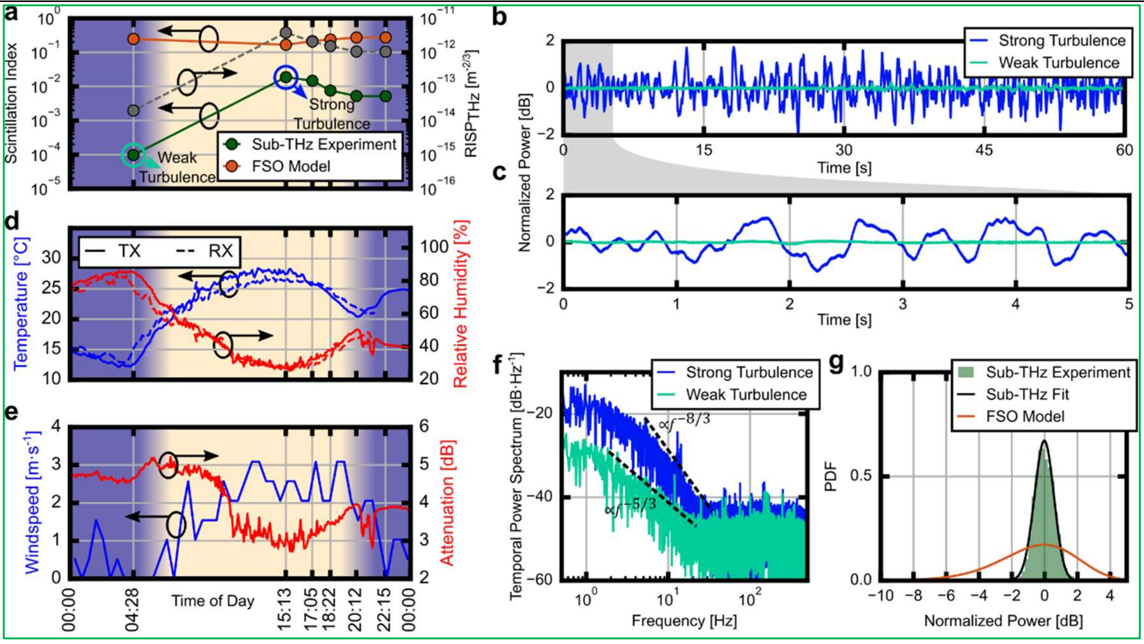

[…] **d** Measured temperature and humidity values at the transmitter (solid line) and receiver (dashed line). ~~and e estimated~~ **e** Wind speed measurement at the airbase Dubendorf[53] and estimated atmospheric attenuation according to ITU-R P.676[38]. […]

Revisions Round 1

Reviewer 1

We thank the reviewer for his overall assessment of our work. We are happy to see that the reviewer finds the paper of interest. We regret that the main points of our manuscripts have not been communicated clearly enough and that the SOTA comparison has been lacking. We answer below the reviewer's comments below in detail to clarify these open points:

The paper reports the achievement of a THz/optical link using plasmonic MZM, and including tests on the field (outdoor). While the paper is interesting, I am not sure to catch the main claims. So far, the work is a combination of several building blocks that are already reported by the same authors, with a new study related to the atmospheric effects. I don't fully understand why the plasmonic modulator approach is helping to analyze the resilience of the THz link over propagation in the atmosphere. To my opinion, the main findings and step forward compared to SoTA should be more clearly claimed to ensure the paper reach good impact.

We are happy to see that the reviewer finds the paper of interest. We regret that the main points of our manuscripts have not been communicated clearly enough. The novelties are as follows:

- This is the highest distance-data-rate product of a sub-THz free-space link
- The success is in part a combination of multiple factors
  - Use of plasmonic components that offer a flat frequency response up to highest speed.
  - Development of system-level improvements based on a novel dual-sideband receiver to increase the SNR and therefore data rate.
- Analysis and measurement of the effect of turbulences on a high data rate 200-300 GHz sub-THz signal in a field trail.

We want to stress that there is no claim that the plasmonic modulator helps to study the atmospheric effect. We apologize that the manuscript failed to communicate the main claim clearly. Plasmonic components help in performing communications at sub-THz frequencies because they offer a flat frequency response up to 1 THz and directly up-mix the sub-THz signal into the optical domain.

To stress the achievement of highest distance-rate product of this work more, we restructured the manuscript to show the comparison of state-of-the-art (Figure 2) at the beginning of the results section.

We have revised the manuscript as follows:

Title:

Photonic-Plasmonic Sub-THz Wireless Link for 6G Demonstrating 160 Gbps over 1400 m in Strong Atmospheric Turbulence

All-Photonic Sub-THz Wireless Link Demonstrating 160 Gbps over 1400 m for 6G Radio Access Networks

Abstract:

[…] Broadband photonic and plasmonic components enabled flat frequency response at high speeds. […] (P.1, L.24)

[…] We assessed power variations due to atmospheric turbulence; the scintillation index remained below 0.019 under strong turbulence, confirming sub-THz link resilience. A theoretical comparison […] (P.1, L.26)

Introduction:
[…] The receiver utilizes direct conversion through an organic-plasmonic Mach-Zehnder modulator (MZM) and a dual-sideband receiver scheme that provides a SNR gain of up to 2 dB[33]. The receiver utilizes direct conversion through an organic-plasmonic Mach-Zehnder modulator (MZM). Compared to prior art, a dual-sideband receiver scheme that provides a SNR gain of up to 2 dB[33] has been employed. We report outdoor measurements of atmospheric turbulence effects on a high data rate sub-THz link, with scintillation indices staying below 0.019 even under strong turbulence. These results confirm the known resilience of sub-THz links to turbulence[34, 35] and, unlike many lab-based studies, provide valuable evidence to the community that sub-THz links perform reliably under real-world outdoor conditions over practical distances exceeding 1 km. For the first time outside laboratory conditions, we measured the impact of atmospheric turbulence on received sub-THz power, with the scintillation index remaining below 0.019 even in strong turbulence. […] (P.3, L.35)

Results:
[…] Sub-THz frequencies may offer a balance of high throughput and resilience towards adverse weather, making them an ideal complement to FSO to ensure high-capacity, reliable wireless communications.

The seamless transition from the optical domain to the sub-THz domain is enabled by the high-speed sub-THz down- und up-mixer as illustrated in Figure 1c. In case of adverse weather conditions, where FSO is blocked, the optical signal at the transmitter side can be re-routed to the sub-THz down-mixer and transmitted via a sub-THz carrier. On the receiver side, the signal is directly up-mixed back to the optical domain by means of a high-speed plasmonic modulator.
A high-speed sub-THz down- and up-mixer enables a seamless transition between the optical and sub-THz domains, as shown in Figure 1c. During adverse weather conditions that disrupt free-space optical (FSO) links, the optical signal at the transmitter can be rerouted through the sub-THz down-mixer and sent over a sub-THz carrier. At the receiver, the signal is directly up-mixed back into the optical domain using a high-speed plasmonic modulator. This seamless switching capability allows the optical and sub-THz links to complement each other in a confluent way, effectively addressing the limitations of each technology.

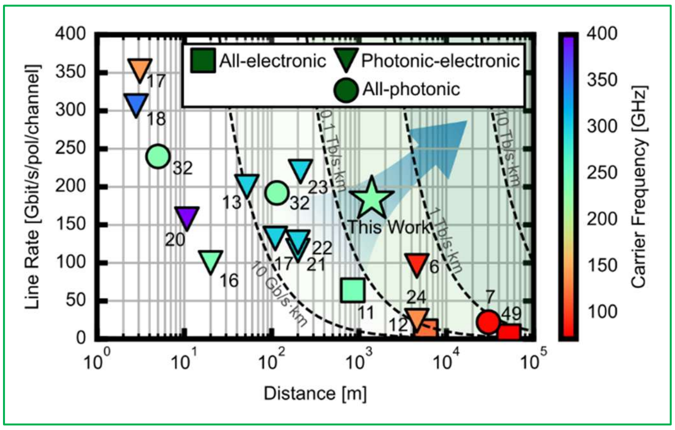

Fig. 2: State-of-the-Art THz-band (0.3-10 THz), sub-THz-band (100-300 GHz), E-band (60-90 GHz) and W-band (75-110 GHz) links. *Line rate per polarization and channel versus transmission distance of state-of-the-art wireless links. The color represents the carrier frequency used in the experiment.*

To show the potential of the all-photonic links, we plot in Figure 2 the transmission distance and achieved line rates for a selection of state-of-the-art all-electronic, photonic-electronic, and all-photonic link demonstrations. Further details to each demonstration are summarized in Table 2. This work shows that high data rate and long distance sub-THz links are indeed achievable by using photonic and plasmonic components. While raising the carrier frequency above 100 GHz brings the advantage of higher bandwidth, it raises a set of technological and operational challenges that need to be addressed. Firstly, we discuss the theoretical considerations for a long distance sub-THz link like atmospheric attenuation and turbulence, including a comparison to FSO communication. Secondly, we demonstrate in a field trial over 1400 m the sub-THz link demonstration (above 100 GHz) with the highest product of line-rate and distance to date, which is enabled by high-speed plasmonic modulators and the dual-sideband receiver. […] (P.5, L.9)

1.*[…] About comparison with FSO, the higher robustness of the THz transmission is expected and was experimentally demonstrated already in 2012 by Moeller & Federici (DOI: 10.1364/JOSAA.29.000179) and several recent studies reported this as well, as an example: Jianjun Ma, Yuheng Song, Mingxia Zhang, Guohao Liu, Weiming Li, John F. Federici, Daniel M. Mittleman, Terahertz channels in atmospheric conditions: Propagation characteristics and security performance, Fundamental Research, 2024. […]*

We thank the reviewer for this comment and the references. We have now referenced these papers. We also agree that there are published studies on atmospheric turbulence and that it is a known result, that sub-THz is more resilient to atmospheric turbulence compared to FSO. However, to the best of our knowledge, all these studies use turbulence chambers to precisely control the conditions in a reproducible way. We believe that measurements of the effect of atmospheric turbulence in a field trial transmitting high data rates over kilometer distances, as one would encounter in a deployed link, are valuable to the community.

We included the reference and adjusted the introduction to stress this point in the following way:

Introdcution:
[…] We report outdoor measurements of atmospheric turbulence effects on a high data rate sub-THz link, with scintillation indices staying below 0.019 even under strong turbulence. These results confirm the known resilience of sub-THz links to turbulence[34, 35] and, unlike many lab-based studies, provide valuable evidence to the community that sub-THz links perform reliably under real-world outdoor conditions over practical distances exceeding 1 km. For the first time outside laboratory conditions, we measured the impact of atmospheric turbulence on received sub-THz power, with the scintillation index remaining below 0.019 even in strong turbulence. […] (P. 3, L. 40)

[…] In order to assess the effects of the atmospheric transmission and discuss the atmospheric contribution, a back-to-back transmission without the air link would be necessary, to highlight the effect of the turbulences and also the stability of the EVM, SNR of the THz link itself, but without air propagation. […]

	We agree with the reviewer, that a back-to-back transmission of the THz link without the air channel are needed to precisely isolate the effect of the sub-THz channel. We have indeed performed short distance indoor experiments to confirm the stability of the system before going to the field trial. Some of these short distance results have been published by members of our group:<br><br>Horst Y*, et al.* Transparent Optical-THz-Optical Link at 240/192 Gbit/s Over 5/115 m Enabled by Plasmonics. *Journal of Lightwave Technology* **40**, 1690-1697 (2022).<br><br>With respect to the comment on the EVM and SNR properties: We answer this comment further below in more detial<br><br>We adjusted the manuscript in the following way to emphasize this point:
	Results:<br>[…] instances during the day using a power meter with an averaging time of 200 µs. To confirm that the origin of the power fluctuations stem from the free-space channel, short range indoor experiments in the laboratory over 5 m have been conducted in prior studies[32], showing that the received power is stable. Therefore, the power fluctuations in this study are attributed to atmospheric turbulence effects. We use a $\Gamma\Gamma$-distribution and Andrew's method[47] to extract the […] (P.15, L.8)
[…] This also stands for the system optical performance: it should also be discussed to properly compare the FSO performance with the THz one in a clear sky condition or with a back-to-back optical transmission. […]	
	We thank the reviewer for his commentary on the comparison of an FSO link to a sub-THz link under more weather conditions. We note that clear sky conditions can still include various forms of turbulences due to temperature gradients, winds, relative humidity, etc. We therefore expanded on our theoretical analysis on how a sub-THz link compares to the FSO link.<br><br>We added the new subplots Fig. 3c and 3d that illustrate the advantages in scintillation index and fading loss by comparing their calculated values.<br><br>While beyond the scope of this paper, it would indeed be preferable that future studies could directly compare all systems side by side: fiber link, FSO link and sub-THz link where we monitor the stability of each property over extended periods of time.
	Results:<br>[…] adaptive optics. Figures 4c and 4d indicate that, under the aforementioned conditions, the scintillation index and fading losses fall within the green-shaded region, indicating a favorable regime for sub-THz relative to FSO links. More information on the calculations can be found in the Supplementary Note 1. […] (P.8, L.28)

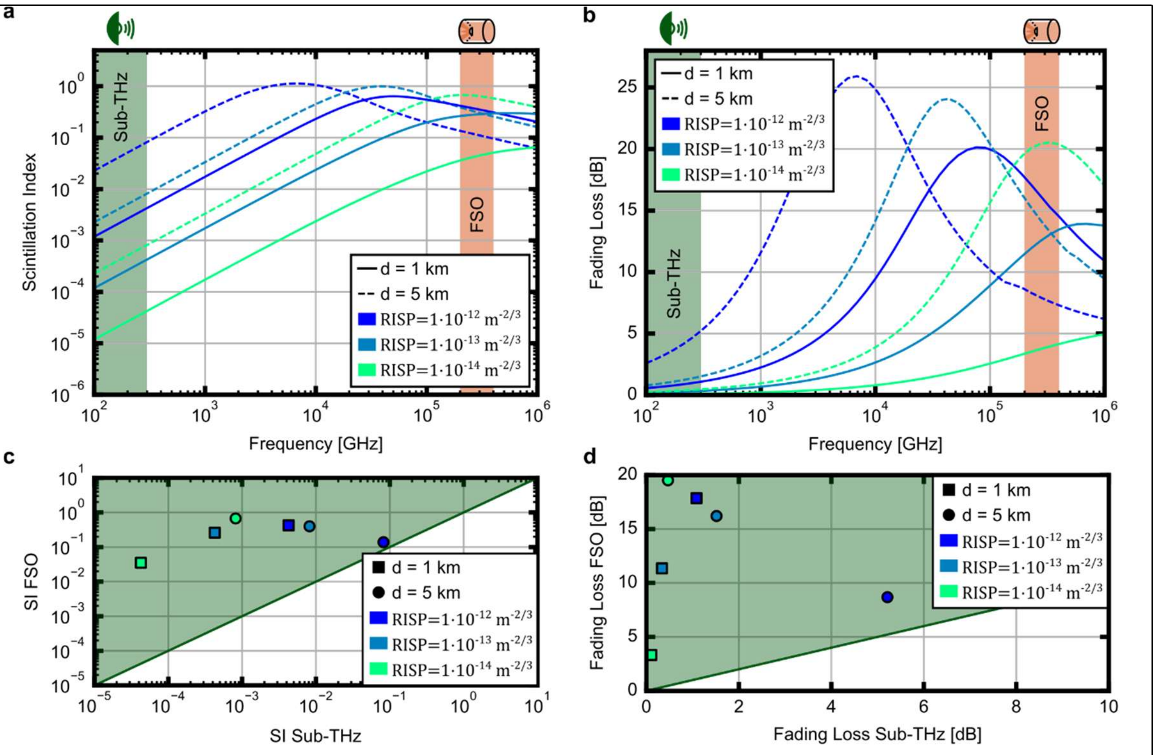

Fig. 4*: Comparison of scintillation index (SI) and fading loss according to Andrew's method between sub-THz and free-space optics (FSO) in weak, moderate, and strong turbulence conditions at link distances of 1 km and 5 km assuming a receiver aperture radius of 2.5 cm. a** *Scintillation index for three turbulence conditions, where the turbulence strengths relate to the refractive index structure parameter values (RISP).* ***b *Additional fading loss that needs to be compensated to have a 99.99 % reliability of the wireless link.* ***(c, d)*** *Comparison of SI and fading loss between FSO (1550 nm) and Sub-THz (300 GHz) in above mentioned conditions. The green shaded area is where sub-THz is advantageous compared to FSO.*

[…] If the focus is to study the effects of the THz signal, why not detecting directly the THz signal at 226 GHz? A lot of receivers in the THz range are available now. If the main claim is the use of the plasmonic modulator that helps to study the atmospheric effect, this should be discussed. […]

We want to stress that there is no claim that the plasmonic modulator helps to study the atmospheric effect. We apologize that the manuscript failed to communicate the main claim clearly. Plasmonic components help in performing communications at sub-THz frequencies because they offer a flat frequency response up to 1 THz and directly up-mix the sub-THz signal into the optical domain.

We have revised the manuscript as follows:

Title:

Photonic-Plasmonic Sub-THz Wireless Link for 6G Demonstrating 160 Gbps over 1400 m in Strong Atmospheric Turbulence

All-Photonic Sub-THz Wireless Demonstrating 160 Gbps over 1400 m for 6G Radio Access Networks

	Abstract:<br>[…] We assessed power variations due to atmospheric turbulence; the scintillation index remained below 0.019 under strong turbulence, confirming sub-THz link resilience. A theoretical comparison […] (P.1, L.26)
2.<br>*In the figure 5, the I/Q maps and SNR correspond to optimal situation or worse case? This is not clear here.*	
	We thank the reviewer for this comment. The I/Q maps and SNR correspond to the best case situation, where scintillation is at a minimum.<br><br>We adjusted the manuscript and the caption of Figure 5 in the following ways:
	Results:<br>[…] combination (purple) in best case conditions where scintillation was at a minimum. The combined SNR of the tributaries with lower baseband power could be improved by the dual-sideband receiver scheme. **b** Constellation […] (P.12, L.35)<br><br>[…]. In Figure 6b, we show the constellation diagram, the bit-error rate (BER) and general mutual information (GMI) of each tributary. These measurements are performed in best case conditions, where scintillation was at a minimum. The achievable information rate (AIR) is […] (P.13, L.28)
[…] I would expect to see some BER variations linked to weather variations here. If the study aim to link atmospheric effects to the THz link performance compared to FSO, impact on the system metrics (like BER) should be shown. […]	
	We agree with the reviewer that measurements of the BER in different atmospheric turbulence conditions would be valuable to study the impact on link performance. This paper shows the potential of the technology and is a demonstrates one of the highest distance-data rate products so far shown. As discussed above, a more thorough is planned to fully assess the stability of the BER in many more conditions.<br><br>We adjusted the manuscript and the following way:
	Discussion:<br>[…] effects on a THz link, showing low scintillation index of 0.019 even under strong turbulence. The promising performance observed in this study highlights the need for future work involving long-term sub-THz link deployment and assessment of BER and EVM across diverse weather conditions. […] (P.17, L.42)
3<br>*About the product data-rate.distance, a full comparison for the readers needs to include additional metrics. In the figure 6, in order to compare the system to the other works, the EVM (%) or BER (Bit Error Rate) achieved should be mentioned. This is also applicable in the table, where the EVM (% or dB) and BER should be included. Here the authors measured the BER performance of their system thus they can compare their performance with others.*	
	We agree with the reviewer, that adding BER and EVM helps comparing the performance of each link.<br><br>Table 2 in the manuscript has been adjusted by adding the pre-FEC BER, together with the type of FEC (HD-FEC/SD-FEC), the overhead in percent and if the FEC is performed online or offline, if this information is available in the publications. As almost none of the

publications give information about the EVM, we decided to only show and compare the BER in Table 2 to keep the table readable.

Results:

**Type[a]**	**Line-rate-distance product**	**Line rate [Gbit/s/pol/**	**Distance [m]**	**Carrier frequency [GHz]**	**BER (pre-FEC)**	**Technology TX/RX**	**Modulation Format**	**Year**	**Author**	**Reference**
P-E	0.8568	306	2.8	350	2.4E-02 (Offline)	UTC-PD/SBD	PS-64QAM-OFDM	2020	Jia et al.	18
P-E	1.0912	352	3.1	140	< 4E-02 (Offline)	UTC-PD/D-Band Mixer	64QAM-PS5.5	2019	Li et al.	17
P-P	1.2	240	5	231	4.6E-02 (Offline)	UTC-PD/PM	QPSK, 16QAM	2022	Horst et al.	32
P-E	1.67	157	10.7	400	2.4E-02 (Offline)	UTC-PD/SBD	16QAM	2022	Jia et al.	20
P-E	2	100	20	237.5	4.5E-03 (Offline)	UTC-PD/InGaAs MMIC	16QAM	2013	Koenig et al.	16
P-E	10.4	200	52	300	6E-03 (Offline)	UTC-PD/GaAs MMIC	16QAM	2024	Dittmer et al.	13
P-E	14.52	132	110	300	1.9E-03 (Offline)	UTC-PD/SBD	16QAM	2020	Harter et al.	19
P-P	22.08	192	115	231	3.2E-02 (Offline)	UTC-PD/PM	QPSK, 16QAM	2022	Horst et al.	32
P-E	23.2	116	200	300	2E-02 (Offline) (20% SD-FEC)	UTC-PD/HM	PDM-QPSK	2024	Cai et al.	21
P-E	25.3	127	200	300	-	UTC-PD/	PS-16QAM DP-OFDM	2024	Tong et al.	22
P-E	47.08	220	214	300	3.75E-03 (Online) (HD-FEC)	UTC-PD/SHM	32QAM	2024	Maekawa et al.	23
All-E	54.4	64	850	240	7.9E-05 (Online)	GaAs HEMT MMIC/"	QPSK	2015	Kallfass et al.	11
All-E	58	10	5800	120	-	InGaAs/InP HEMT MMIC/"	ASK	2010	Hirata et al.	12
P-E	110.4	24	4600	135	8.6E-03 (Offline) (15% SD-FEC)	-	OFDM-16QAM	2022	Li et al.	24
E-E	187.2	3.6	52000	72	7E-02 (Offline)	E-Band Mixer/''	64QAM	2024	Guo et al.	49
P-E	441.6	96	4600	87.5	2.4E-02 (Offline) (15% SD-FEC)	PD/E-Band Mixer	PDM-16QAM	2023	Li et al.	6
P-P	857.6	32	26800	76.0	2.0E-02 (Offline) (15% SD-FEC)	PD/Phase Modulator	QPSK	2024	Liu et al.	7

P-P	257.6	184	1400	230	3.83E-02 (Offline)	UTC-PD/Plas. Modulator	QPSK, 8QAM, 16QAM	2024		This work

4

About the distance, as 12 dBm of output power is used using amplifier, the transmission distance is obviously enhanced compared to short distance demos, I think this is more an engineering step forward rather that a new finding.

We thank the reviewer for the comment, and we agree that higher distances require more output power and the use of amplifiers. As the scope of the journal also involves applied, and engineering sciences, we argue that the engineering aspect of this work is of value to the community. Compared to laboratory demonstrations, bridging the distance of 1.4 km involves engineering, like positioning and alignment of the antenna, the antenna themselves, the use of amplifiers, which introduce bandwidth limitations, noise and nonlinear effects that come with higher power. Furthermore, turbulence effects may limit the systems performance.

5

I have also some technical questions that are not described:

- How the SNR is calculated?

We thank the reviewer for this comment. We added the calculation of the SNR for each tributary in a new section "Calculation of SNR" in the supplementary material.

We adjusted the manuscript and supplementary information in the following way:

Supplementary Information:

Supplementary Note 2: Calculation of SNR

This section describes how the signal-to-noise ratio (SNR) of each tributary is calculated after digital signal processing (DSP). The SNR of the received tributary signal $Y = N(0, \sigma_s + \sigma_n)$ is calculated by comparing it to the transmitted signal $X = N(0, \sigma_s)$ and assuming white Gaussian noise (AWGN) is added to Y. The noise power N_0 is defined as

$$N_0 = \mathrm{Var}(Y - hX) = \mathrm{Var}(Y) + h^2\mathrm{Var}(X) - 2h\mathrm{Cov}(X, Y), \qquad (7)$$

where X is the normalized transmitted signal, Y is the normalized received signal and h a proportionality factor. The noise power N_0 is minimized by choosing $h = \mathrm{Cov}(X, Y)/\mathrm{Var}(X)$. Inserting h into (7) yields

$$N_0 = \mathrm{Var}(Y) - \frac{\mathrm{Cov}(X, Y)^2}{\mathrm{Var}(X)}. \qquad (8)$$

Calculating the SNR yields.

$$\mathrm{SNR} = \frac{\mathrm{Var}(Y) - N_0}{N_0} = \frac{\mathrm{Var}(Y)}{N_0} - 1 = \frac{1}{1 - \frac{\mathrm{Cov}(X, Y)^2}{\mathrm{Var}(X)\mathrm{Var}(Y)}} - 1. \qquad (9)$$

Results:

[…] The resulting baseband power spectral density is shown in Figure 6a (grey color), along with the SNRs for each tributary. The calculation of the SNR is described in Supplementary Note 2. The SNRs of the lower sideband (LSB) and upper sideband (USB) are also presented, […] (P.13, L.15)

6

- Why the tap number of the FFE is very high compared to CMA and RDE?

We thank the reviewer for this comment, and we agree that the tap number of the FFE is very high. The tap number of the FFE could potentially be scaled down and optimized.

We adjusted the manuscript in the following way:

[…] filter taps of the CMA, RDE, and FFE was set to 41, 41 and 223, respectively. The number of taps was not optimized and could potentially be reduced without loss of performance. Combining the two baseband signals was achieved by utilizing a butterfly extension of the FFE. […] (P.13, L.7)

7

- The received signals show very noisy I/Q maps with limited signal to noise ratio. Did the authors analyzed the origin of this noise (or how they confirm that the ASE noise is the main limitation)?

We thank the reviewer for this remark. The I/Q maps look noisy indeed. We could easily resort to I/Q maps that look less noisy by reducing the symbol rate. Yet, these diagrams show the community up to what speed error free operation (error free if FEC is applied) can be performed. Therefore, the I/Q maps are supposed to be taken at the point where we transition to the limit of where data can still be recovered.

With respect to the reviewer's question on the noise source: We analyzed the limiting noise sources in a link budget, which we added to the supplementary information. We found that the ASE-LO beat noise of EDFA 2, and noise of the second sub-THz amplifier are the dominant noise sources in the system. Additionally, we have analyzed the measured SNR for each tributary of both sidebands separately and the combined SNR. By employing the dual-sideband receiver and by comparing those SNRs we can distinguish that the noise source is either added prior of after the plasmonic modulator. If the dominant noise is added after the modulator, e.g. in the EDFA 2, the noise will be uncorrelated, and an SNR gain is observed. However, if the noise is already present in the RF signal prior to the modulator, the noise in both sidebands is correlated and receiving both sidebands will not give any SNR improvement. Therefore, in tributaries 5-8 the limiting noise is the ASE-LO beat noise of EDFA 2, while tributaries 1-4 show more correlated noise that results in less gain. Based on a link budget, we attribute the noise source to the sub-THz amplifier.

To make this clearer, we adjusted the manuscript in the following way:

Results:

[…] is the amplified spontaneous emission noise introduced by the low-noise EDFA 2 after point D, see Figure 5a. On the other hand, the dual-sideband receiver's SNR improvement of tributaries 1-4 was below 2 dB, indicating that the dominant noise in both sidebands is correlated and originates prior to the plasmonic modulator. This finding agrees well with the theoretical link budget, as discussed in the supplementary note 3. […] (P.13, L.22)

Supplementary Information:

Supplementary Note 3: Link Budget

Supplementary Table 3 presents the theoretical link budget for each tributary. Signal power spectral density (PSD) is propagated through the channel using both measured and theoretical power spectra of the system components and the free-space link, see Supplementary Figure 2 and Figure 7e. Noise from sub-THz amplifiers is added to the signal and propagated through the channel. The SNR of the preamplifier coherent receiver is limited by the ASE-LO beat noise and is estimated using

$$\mathrm{SNR} = \frac{P_s}{hf n_{\mathrm{sp}} B}, \qquad \text{(S10)}$$

where P_s is the optical signal power, hf the photon energy, n_{sp} the SNR degradation from the shot-noise limit and B the bandwidth[5]. For high gain values, the SNR degradation of the amplifier chain is approximately $n_{sp} \approx F_{tot}/2$, where F_{tot} is the combined noise factor of the receiver chain, which consist of low-noise EDFA 2, loss of the demultiplexer and polarization beam splitter and preamplifier EDFA 3, see Figure 4d. According to the Friis formula, the total noise figure can be calculated by

$$F_{\mathrm{tot}} = F_{\mathrm{EDFA2}} + \frac{L_{\mathrm{DEMUX}} F_{\mathrm{EDFA3}} - 1}{G_{\mathrm{EDFA2}}}, \qquad \text{(S11)}$$

where F_{EDFA2} and G_{EDFA2} are the noise factor and gain of the low-noise EDFA 2, L_{DEMUX} the loss of the demultiplexer and polarization beam splitter and F_{EDFA3} the noise factor of the preamplifier EDFA 3. To estimate the SNR after the dual-sideband receiver, the ASE-LO beat noise power was reduced by 3 dB. The dominant noise sources were the sub-THz amplifier and the low-noise EDFA 2 at the receiver. The dual-sideband receiver's SNR gain matches the measured results well, showing that tributaries with lower optical sideband power are primarily limited by ASE-LO beat noise and benefit the most from the dual-sideband approach.

Supplementary Tab. 3: Theoretical link budget calculation for each tributary.

	Unit	Tributary							
Tributary Number		1	2	3	4	5	6	7	8
Bandwidth	GHz	8	8	8	8	8	8	8	8
Optical Launch Power (point A)	dBm	3.2	1.6	-1.2	2.3	-0.7	1.4	0.9	4.5
Optical Launch SNR (point A)	dB	17.2	19.7	21.3	23.1	21.9	20.9	18.6	16.2
Sub-THz Launch Power (point C)	dBm	2.5	4.7	4.3	2.7	-0.3	0.0	-0.3	5.1
Sub-THz Launch SNR (point C)	dB	17.1	19.6	21.1	22.8	21.4	20.6	18.4	16.2
Free-Space Path Loss	dB	-141.5	-141.7	-142	-142.3	-142.6	-142.8	-143.1	-143.3
Attenuation	dB	-5	-4.5	-4.5	-4.6	-4.8	-5	-5.3	-5.7
Antenna Gain	dB	111.7	111.7	111.7	111.7	111.7	111.7	111.7	111.7
Excess Loss	dB	7.0	7.0	7.0	7.0	7.0	7.0	7.0	7.0
Sub-THz Received Power	dBm	-39.2	-36.8	-37.5	-39.4	-42.9	-43.1	-44.1	-39.1
Sub-THz Power at Modulator	dBm	-14.1	-9.9	-11.1	-15.0	-21.8	-23.6	-22.9	-19.2
Optical Sideband Power (point D)	dBm	-44.5	-40.3	-41.5	-45.4	-52.1	-54	-53.2	-49.6
SNR Single Sideband (point E/F)	dB	12.3	15.8	15.6	12.7	6.2	4.4	5.0	8.3
SNR Dual-Sideband	dB	13.5	16.8	16.9	14.5	8.3	6.5	7.0	10.0

8

- It is said that main noise source in the system is the EDFA noise (ASE), what is the noise figure of this EDFA?

We adjusted the manuscript in the following way, to add the noise figure and gain of the EDFA.

[…] The optical signal is amplified by a low-noise EDFA EDFA 2 with a gain of 35 dB and a noise figure of 3.4 dB, transmitted through 6 km of SSMF before it is received in the dual-sideband optical coherent receiver[33], emulating the […] (P.12, L.4)

9

- As mentioned by authors, the plasmonic modulator is one of the important part of the system, however I didn't find any details of the impedance matching, the interconnection of the 226 GHz for modulator driving as well as the driving power to the modulator. I believe this is important parameters and this should be described. […]

We thank the reviewer for this remark and apologize that we didn't describe this important aspect in the original manuscript.

We adjusted the manuscript to describe the impedance matching and connection with the modulator in the following way. Furthermore, we added the "Sub-THz Power at Modulator" to the link budget in the Supplementary Note 3.

	Results:<br>[…] modulator[28] (MZM) via a ground-signal-ground (GSG) RF probe with a characteristic impedance of 50 Ω. The total sub-THz power at the plasmonic MZM was -5.6 dB. The electrodes of the MZM are modeled as a capacitive load, with an estimated capacitance of 5 fF. Operating well below the cut-off frequency, the MZM presents a high impedance and behaves effectively as an open circuit, resulting in the reflection of the incident RF power back toward the probe and amplifier. Under these conditions, the voltage amplitude at the modulator input is 0.3 V. The plasmonic MZM, which is biased to the null point through wavelength We thank the reviewer for this remark. […] (P.11, L.38)
10<br>*[…] Also, the noise figure/ the P1dB (compression point) of the amplifiers used should be discussed as this typically impacts a lot the performances of the overall system as the noise figure of THz amplifier is yet not very good.*	
	We thank the reviewer for this comment.<br><br>We added the saturated output power and P1dB of the sub-THz amplifier. We agree with the reviewer, that noise figure of these amplifiers is not very good yet. Unfortunately, the datasheet of the manufacturer didn't specify the noise figure of the amplifiers and to date, we don't have the equipment to characterize it by ourselves.<br><br>In our case, as we operate the sub-THz amplifier at the transmitter 4 dB below the saturated output power, we couldn't observe any non-linear distortions. This is underlined by two instances in the measured IQ diagrams.<br>• The symbols are equally spaced with gaussian noise which points away from non-linearities.<br>• Applying a Volterra algorithm with 1 second and 1 third-order tap does not improve the SNR pointing away from non-linearities.<br><br>We added the following sentences to the manuscript to point this out more clearly.
	Results:<br>[…] The sub-THz signal is amplified by a commercial electrical sub-THz amplifier with a gain of 24 dB, yielding a typical launch power of 12 dBm (point C). The sub-THz amplifiers at the down- and up-mixer have a saturated output power of 16 dBm and 1 dB compression point (P1dB) of 9 dB. […] (P.11, L.18)<br><br>[…] Combining the two baseband signals was achieved by utilizing a butterfly extension of the FFE. Despite the two sub-THz amplifiers in the link, non-linear equalization could not improve the SNR and BER as the amplifiers were not operated close to saturation. Therefore, non-linear equalization was omitted, reducing the computational complexity of the DSP. […] (P.13, L.9)

We thank the reviewer again for his constructive comments. By addressing each of the remarks and improving our manuscript with the provided feedback, we hope that now the paper clearly communicates the open points on the main achievements. Moving from distinct component based lab experiments to large, combined field trials show the potential and challenges of this technology for future use in industry. Using an all-photonic approach with plasmonic

modulators could show the highest distance-data rate product for sub-THz links (> 100 GHz carrier frequency).

Reviewer 2

I've reviewed the paper titled "Photonic-Plasmonic Sub-THz Wireless Link for 6G Demonstrating 160 Gbps over 1400 m in Strong Atmospheric Turbulence" submitted to Nature Communication. This is an extension work which previously reported in OFC 2024. The results are solid, the topics is of significance to next generation network. However, the paper's innovation requires further clarification, and several details need to be addressed to strengthen the presentation.

We are happy to see that the reviewer finds the results solid, and we are thankful for the comments, which improve the presentation of the paper. We address each of the reviewer's inputs in detail below.

1

The primary contribution of this paper is the outdoor system demonstration of a sub-THz link over 1.4 km using Plasmonic MZMs.
-There are already 500GHz THz-to-optical conversion by plasmonic MZM as reported in Ref. 24, and also some experimental demonstrations been demonstrated in Ref. 28.
The novelty of this work compared to these references remains unclear. Have there been any device-level improvements? The paper should clearly articulate its advancements over the state-of-the-art.

We thank the reviewer for the comment.

We would like to communicate the novelty of this paper more clearly, which are:

- This is the highest distance-rate product of a sub-THz free-space link
- The success is in part a combination of multiple factors
  - Use of plasmonic components that offer a flat frequency response up to highest speed.
  - Development of system-level improvements based on a novel dual-sideband receiver to increase the SNR and therefore data rate.
  - Assembly of the devices and components such as plasmonic modulators, antennas, antenna-positioners, etc. into a system demonstration in a field trial.
- Analysis and measurement of the effect of turbulences on a high data rate 200-300 GHz sub-THz signal in a field trial.

The advancement over state-of-the-art is the record high distance-rate product of a sub-THz free-space link. To stress this more, we restructured the manuscript to show the comparison of state-of-the-art (Figure 2) at the beginning of the results section.

Compared to Ref. 28, the dual-sideband receiver was developed and incorporated successfully in the system. Furthermore, the air channel is changed from 115 m indoors to 1.4 km outdoors, which comes with challenges, such as alignment, increased FSPL, attenuation, atmospheric turbulence, etc.

We do not report on any device level improvements.

To clarify these points, the manuscript has been adjusted in the following way:

Abstract:
[…] We assessed power variations due to atmospheric turbulence; the scintillation index remained below 0.019 under strong turbulence, confirming sub-THz link resilience. A theoretical comparison […] (P.1, L.26)

Introduction:
[…] The receiver utilizes direct conversion through an organic-plasmonic Mach-Zehnder modulator (MZM) and a dual-sideband receiver scheme that provides a SNR gain of up to 2 dB[33]. The receiver utilizes direct conversion through an organic-plasmonic Mach-Zehnder modulator (MZM). Compared to prior art, a dual-sideband receiver scheme that provides a SNR gain of up to 2 dB[33] has been employed. We report outdoor measurements of atmospheric turbulence effects on a high data rate sub-THz link, with scintillation indices staying below 0.019 even under strong turbulence. These results confirm the known resilience of sub-THz links to turbulence[34, 35] and, unlike many lab-based studies, provide valuable evidence to the community that sub-THz links perform reliably under real-world outdoor conditions over practical distances exceeding 1 km. For the first time outside laboratory conditions, we measured the impact of atmospheric turbulence on received sub-THz power, with the scintillation index remaining below 0.019 even in strong turbulence. […] (P.3, L.35)

Results:
[…] Sub-THz frequencies may offer a balance of high throughput and resilience towards adverse weather, making them an ideal complement to FSO to ensure high-capacity, reliable wireless communications.

The seamless transition from the optical domain to the sub-THz domain is enabled by the high-speed sub-THz down- und up-mixer as illustrated in Figure 1c. In case of adverse weather conditions, where FSO is blocked, the optical signal at the transmitter side can be re-routed to the sub-THz down-mixer and transmitted via a sub-THz carrier. On the receiver side, the signal is directly up-mixed back to the optical domain by means of a high-speed plasmonic modulator.
A high-speed sub-THz down- and up-mixer enables a seamless transition between the optical and sub-THz domains, as shown in Figure 1c. During adverse weather conditions that disrupt free-space optical (FSO) links, the optical signal at the transmitter can be rerouted through the sub-THz down-mixer and sent over a sub-THz carrier. At the receiver, the signal is directly up-mixed back into the optical domain using a high-speed plasmonic modulator. This seamless switching capability allows the optical and sub-THz links to complement each other in a confluent way, effectively addressing the limitations of each technology.

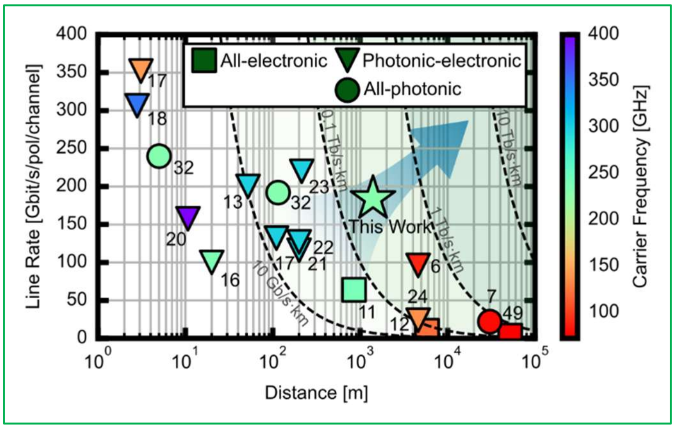

Fig. 2: State-of-the-Art THz-band (0.3-10 THz), sub-THz-band (100-300 GHz), E-band (60-90 GHz) and W-band (75-110 GHz) links. *Line rate per polarization and channel versus transmission distance of state-of-the-art wireless links. The color represents the carrier frequency used in the experiment.*

To show the potential of the all-photonic links, we plot in Figure 2 the transmission distance and achieved line rates for a selection of state-of-the-art all-electronic, photonic-electronic, and all-photonic link demonstrations. Further details to each demonstration are summarized in Table 2. This work shows that high data rate and long distance sub-THz links are indeed achievable by using photonic and plasmonic components. While raising the carrier frequency above 100 GHz brings the advantage of higher bandwidth, it raises a set of technological and operational challenges that need to be addressed. Firstly, we discuss the theoretical considerations for a long distance sub-THz link like atmospheric attenuation and turbulence, including a comparison to FSO communication. Secondly, we demonstrate in a field trial over 1400 m the sub-THz link demonstration (above 100 GHz) with the highest product of line-rate and distance to date, which is enabled by high-speed plasmonic modulators and the dual-sideband receiver. […] (P.5, L.9)

2

The title may cause confusion among readers. It is well established that atmospheric turbulence is generally not an issue for millimeter-wave (MMW) and THz signals but poses significant challenges for free-space optical (FSO) channels. The current title might mislead readers to believe that turbulence has become a problem for THz communication or that the paper solves the turbulence issue for FSO. Clarifying this or change the title is recommended.

We thank the reviewer and agree that the title might lead to confusion. We adjusted the title to stress the main achievement of the publication which is the product of distance and data rate.

Title:

Photonic-Plasmonic Sub-THz Wireless Link for 6G Demonstrating 160 Gbps over 1400 m in Strong Atmospheric Turbulence

All-Photonic Sub-THz Wireless Link Demonstrating 160 Gbps over 1400 m for 6G Radio Access Networks

3

While the authors compare THz and FSO performance under turbulent conditions, no clear technical contributions or innovations are evident in this comparison. Section III, "Influence of Atmospheric Turbulence on Wireless Link," merely reiterates a well-known result. Therefore, the innovation or contributions requires further clarification in this section.

We thank the reviewer for this remark. We agree that there are published studies on atmospheric turbulence and that it is a known result, that sub-THz is more resilient to atmospheric turbulence compared to FSO. To the best of our knowledge, all these studies use turbulence chambers to precisely control the conditions in a reproducible way. We believe that measurements of the effect of atmospheric turbulence in a field trial transmitting high data rates over kilometer distances, as one would encounter in a deployed link, are valuable to the community. More importantly, we believe that is necessary to verify that turbulences do not matter when both distance and modulation format and bit rates have been increased.

We adjusted the manuscript in the following way:

	Introduction:<br>[…] We report outdoor measurements of atmospheric turbulence effects on a high data rate sub-THz link, with scintillation indices staying below 0.019 even under strong turbulence. These results confirm the known resilience of sub-THz links to turbulence[34, 35] and, unlike many lab-based studies, provide valuable evidence to the community that sub-THz links perform reliably under real-world outdoor conditions over practical distances exceeding 1 km. For the first time outside laboratory conditions, we measured the impact of atmospheric turbulence on received sub-THz power, with the scintillation index remaining below 0.019 even in strong turbulence. The paper also compares FSO and sub-THz links, highlighting sub-THz's greater […] (P.3, L.40)
4<br>*The authors employ terms like All-E (all-electronic), P-E (photonic-electronic), and P-P (photonic-plasmonic) to describe system setups. For instance, P-E refers to a photonics-based transmitter (UTC-PD) and an electronics-based receiver. However, the term “photonic-photonic” would be more appropriate as it encompasses a broader range of solutions, whereas “photonic-plasmonic” excludes other photonic up (or down)-conversion methods, such as thin-film lithium niobate-based THz down-conversion.*<br>*Some of the latest experimental achievements in all-photonic up- and down-conversion solutions have not been included. Relevant references such as:*<br>*P. T. Dat et al., "151.5-GHz Sub-THz Signal Reception and Down-Conversion Using All-Optical Technology," 2024 Optical Fiber Communications Conference and Exhibition (OFC), San Diego, CA, USA, 2024, pp. 1–3.*	
	We thank the reviewer and agree that photonic-plasmonic excludes other technologies like TFLN. We adjusted the manuscript and replaced “photonic-plasmonic” with “all-photonic” throughout the manuscript. Furthermore, we added the reference the reviewer suggested and adjusted the introduction in the following way:
	Introduction:<br>[…] prevented a direct up-mixing of the sub-THz signals to the optical domain. Progress with the development of plasmonic modulators has brought up a modulator solution offering bandwidths in excess of 500 GHz[26] and most recently up to 1 THz[27] that in addition requires low driving voltages and small footprint[28]. Recently, direct up-mixing at 152 GHz was achieved by employing thin-film lithium niobate (TFLN) modulators, demonstrating 80 Gb/s back-to-back transmission[29]. While TFLN allow low optical insertion losses, the electro-optic bandwidth drops beyond 100 GHz due to the difficulty of phase matching the RF and optical wave. Plasmonic modulators on the other hand have brought up a modulator solution offering bandwidths in excess of 500 GHz[26] and most recently up to 1 THz[27] that in addition requires low driving voltages and small footprint[28]. Such plasmonic-based. Such plasmonic-based […] (P.3, L.17)
5<br>*Additionally, Table 2 only lists the distance-rate product for frequencies above 100 GHz, omitting notable demonstrations around 100 GHz in the E or W band (75–110 GHz). Including these would provide a more comprehensive comparison.*	
	We thank the reviewer for this comment and agree, that there are notable demonstrations in the E and W band which lie at the edge of the sub-THz regime.<br><br>We adjusted the manuscript in the following way:
	[…] Yet, FSO links can be strongly degraded by atmospheric turbulence effects and adverse weather, especially fog[4]. A solution offering sufficient bandwidth to transmit the high capacities signals as well as robustness against adverse weather and atmospheric turbulences[5] are wireless links operating at sub-THz above frequencies above 100 GHz. A solution offering sufficient bandwidth to transmit the high capacitiy signals as well as

robustness against adverse weather and atmospheric turbulences[5] are wireless links operating in the E/W (60 – 110 GHz) and in the sub-THz band (100 – 300 GHz). Demonstrations in the E/W band have achieved channel line rates of 96 Gbit/s over 4.6 km[6] and 32 Gbit/s over 28.8 km[7]. In case even higher data rates above 100 Gbit/s are required, sub-THz frequencies offer more bandwidths. Unlike fiber, these sub-THz links can be rapidly deployed permanently or on a temporary basis to enhance network capacity in specific areas during events such as emergency situations. These sub-THz links offer high data rates of >100 Gbit/s and unlike fiber, wireless links can be rapidly deployed permanently or on a temporary basis to enhance network capacity in specific areas during events such as emergency situations. Furthermore, in line-of-sight conditions, a direct […] (P.2, L.22)

Results:

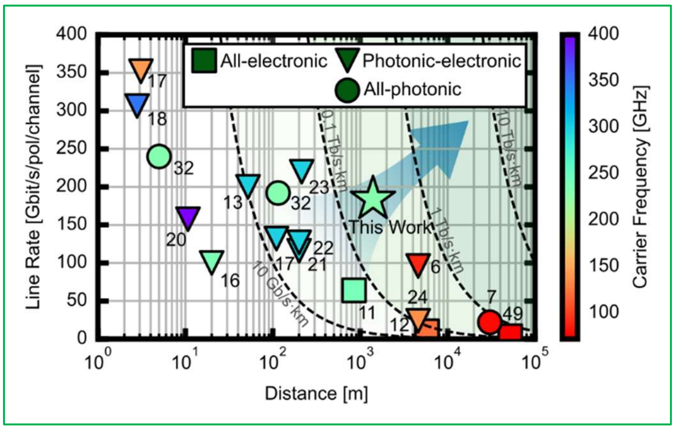

Fig. 2: State-of-the-Art THz-band (0.3-10 THz), sub-THz-band (100-300 GHz), E-band (60-90 GHz) and W-band (75-110 GHz) links. *Line rate per polarization and channel versus transmission distance of state-of-the-art wireless links. The color represents the carrier frequency used in the experiment.*

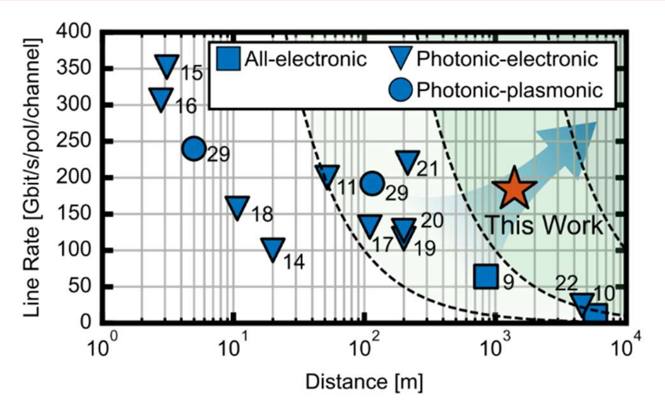

Fig. 6: State-of-the-Art Sub-THz links. *Line rate per polarization and channel versus distance of state-of-the-art sub-THz link carrier frequencies above 100 GHz.*

Tab. 2: State-of-the-Art sub-THz-band (100-300 GHz), E-band (60-90 GHz) and W-band (75-110 GHz) links sorted by distance-rate-product.

Tab. 2: State-of-the-Art sub-THz links with carrier frequencies above 100 GHz sorted by distance-rate-product.

Type^a	Distance-rate-product [Gbit/s·km]	Line rate [Gbit/s/pol/channel]	Distance [m]	Carrier frequency [GHz]	BER (pre-FEC)	Technology TX/RX	Modulation Format	Year	Author	Reference
…	…	…	…	…	…	…	…	…	…	…

P-E	110.4	24	4600	135	8.6E-03 (Offline) (15% SD-FEC)	-	OFDM-16QAM	2022	Li et al.	24
E-E	187.2	3.6	52000	72	7E-02 (Offline)	E-Band Mixer/”	64QAM	2024	Guo et al.	49
P-E	441.6	96	4600	87.5	2.4E-02 (Offline) (15% SD-FEC)	PD/E-Band Mixer	PDM-16QAM	2023	Li et al.	6
P-P	857.6	32	26800	76.0	2.0E-02 (Offline) (15% SD-FEC)	PD/Phase Modulator	QPSK	2024	Liu et al.	7
P-P	257.6	184	1400	230	3.83E-02 (Offline)	UTC-PD/Plas. Modulator	QPSK, 8QAM, 16QAM	2024		This work

6

In the dual-band receiver, which factor has a greater impact on system performance: the insertion loss caused by the wavelength demultiplexer or the gain provided by combining two sidebands? How can these factors be balanced in the system design?

We thank the reviewer for this interesting question.

The quality of the signal depends on the SNR at the receiver. The total noise figure ($\mathrm{NF_{tot}} = 10\log(F_{\mathrm{tot}})$) of the receiver chain (low-noise EDFA 2 → DEMUX → EDFA 3 → Coherent RX) can be calculated using the Friis formula. In our case this yields

$$F_{\mathrm{tot}} = F_{\mathrm{EDFA2}} + \frac{L_{\mathrm{DEMUX}} F_{\mathrm{EDFA3}} - 1}{G_{\mathrm{EDFA2}}},$$

where F_{EDFA2} and G_{EDFA2} are the noise factor and gain of the low-noise EDFA, L_{DEMUX} the loss of the demultiplexer and polarization beam splitter and F_{EDFA3} the noise factor of the preamplifier EDFA 3. With the components used in our experiment, the total noise figure only increases by 0.2% with the additional 8 dB of loss of the demultiplexer, which is negligible. Therefore, in this regime, there is no trade-off between the demultiplexer loss and dual-sideband receiver gain.

To highlight, that there is a second EDFA (EDFA 3) used as a preamplifier for the coherent receiver, we added a subfigure d in Figure 5 showing the detailed schematic of the dual-sideband receiver. Furthermore, we added a theoretical link budget calculation in the supplementary information.

Results:

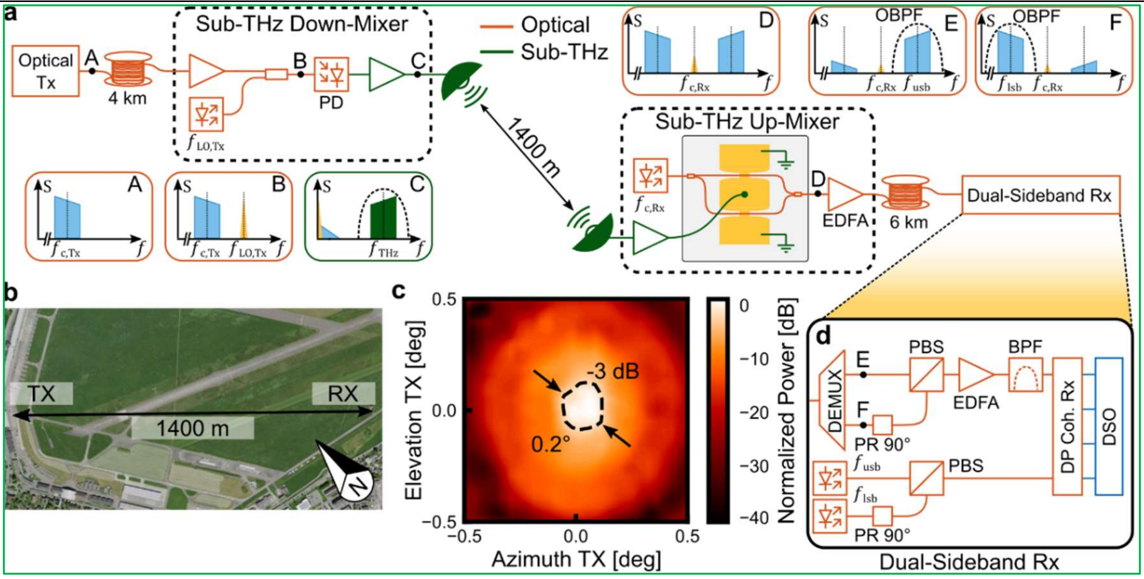

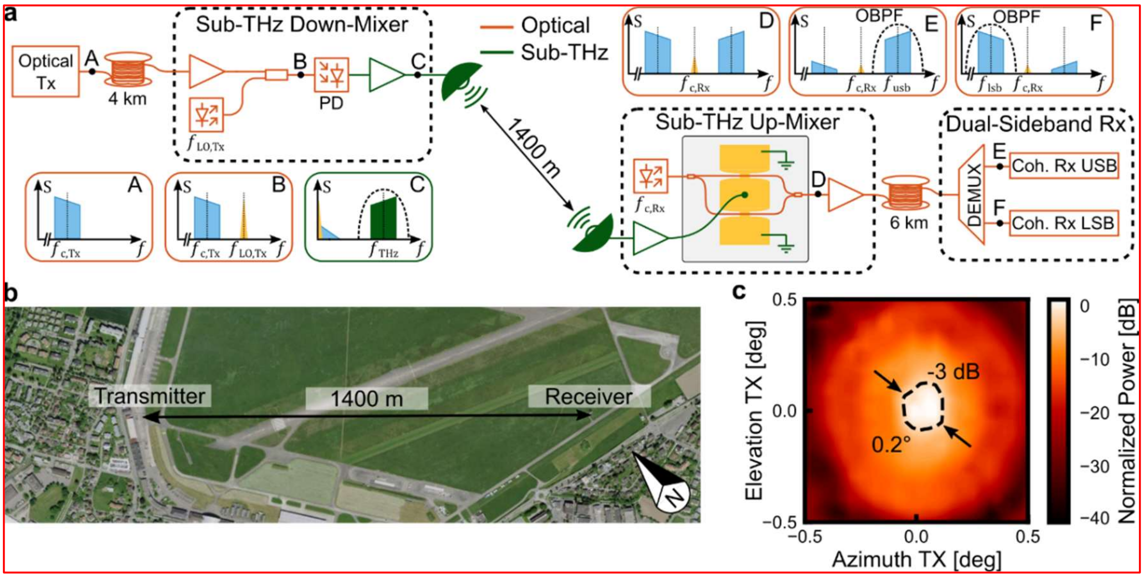

[…] **d** Implementation of the dual-sideband receiver which shifts one sideband to the orthogonal polarization and uses a commercial dual-polarization coherent receiver (DP Coh. Rx) and a digital sampling oscilloscope (DSO) to receive and sample the signals. […] (P.10, L.19)

[…] connection to the baseband unit (BBU). The dual-sideband receiver incorporates a wavelength demultiplexer to separate the two sidebands (point E and F). The polarization of the lower sideband (LSB) is rotated by 90° and is combined again with the upper sideband (USB) via a polarization beam splitter. The signal is amplified by a high-gain EDFA, bandpass filtered and processed by a dual-polarization heterodyne coherent receiver (DP Coh. Rx). This EDFA has a noise figure of 4.5 dB and is operated in power-controlled mode, which keeps the input power into the coherent receiver stable. Both sidebands are converted to baseband, where the electrical baseband signal is sampled by a high-speed digital sampling oscilloscope (DSO). The dual-sideband receiver incorporates a wavelength demultiplexer to separate the two sidebands (point E and F), which are then processed by two heterodyne coherent receivers (Coh. Rx), converting both sidebands to baseband, where the electrical baseband signal is sampled by a high-speed digital sampling oscilloscope (DSO). Digital signal processing […] (P.12, L.6)

Supplementary Information:

Supplementary Note 3: Link Budged

Supplementary Table 3 presents the theoretical link budget for each tributary. Signal power spectral density (PSD) is propagated through the channel using both measured and theoretical power spectra of the system components and the free-space link, see Supplementary Figure 2 and Figure 7e. Noise from sub-THz amplifiers is added to the signal and propagated through the channel. The SNR of the preamplifier coherent receiver is limited by the ASE-LO beat noise and is estimated using

$$\mathrm{SNR} = \frac{P_\mathrm{s}}{hf n_\mathrm{sp} B}, \qquad \text{(S10)}$$

where P_s is the optical signal power, hf the photon energy, n_sp the SNR degradation from the shot-noise limit and B the bandwidth[5]. For high gain values, the SNR degradation of the amplifier chain is approximately $n_\mathrm{sp} \approx F_\mathrm{tot}/2$, where F_tot is the combined noise factor of the receiver chain, which consist of low-noise EDFA 2, loss of the demultiplexer and polarization beam splitter and preamplifier EDFA 3, see Figure 4d. According to the Friis formula, the total noise figure can be calculated by

$$F_\mathrm{tot} = F_\mathrm{EDFA2} + \frac{L_\mathrm{DEMUX} F_\mathrm{EDFA3} - 1}{G_\mathrm{EDFA2}}, \qquad \text{(S11)}$$

where F_EDFA2 and G_EDFA2 are the noise factor and gain of the low-noise EDFA 2, L_DEMUX the loss of the demultiplexer and polarization beam splitter and F_EDFA3 the noise factor of the preamplifier EDFA 3. To estimate the SNR after the dual-sideband receiver, the ASE-LO beat noise power was reduced by 3 dB. The dominant noise sources were the sub-THz amplifier and the low-noise EDFA 2 at the receiver. The dual-sideband receiver's SNR gain matches the measured results well, showing that tributaries with lower optical sideband power are primarily limited by ASE-LO beat noise and benefit the most from the dual-sideband approach.

Supplementary Tab. 3: Theoretical link budget calculation for each tributary.

	Unit	Tributary							
Tributary Number		1	2	3	4	5	6	7	8
Bandwidth	GHz	8	8	8	8	8	8	8	8
Optical Launch Power (point A)	dBm	3.2	1.6	-1.2	2.3	-0.7	1.4	0.9	4.5
Optical Launch SNR (point A)	dB	17.2	19.7	21.3	23.1	21.9	20.9	18.6	16.2
Sub-THz Launch Power (point C)	dBm	2.5	4.7	4.3	2.7	-0.3	0.0	-0.3	5.1
Sub-THz Launch SNR (point C)	dB	17.1	19.6	21.1	22.8	21.4	20.6	18.4	16.2
Free-Space Path Loss	dB	-141.5	-141.7	-142	-142.3	-142.6	-142.8	-143.1	-143.3
Attenuation	dB	-5	-4.5	-4.5	-4.6	-4.8	-5	-5.3	-5.7
Antenna Gain	dB	111.7	111.7	111.7	111.7	111.7	111.7	111.7	111.7
Excess Loss	dB	7.0	7.0	7.0	7.0	7.0	7.0	7.0	7.0
Sub-THz Received Power	dBm	-39.2	-36.8	-37.5	-39.4	-42.9	-43.1	-44.1	-39.1
Sub-THz Power at Modulator	dBm	-14.1	-9.9	-11.1	-15.0	-21.8	-23.6	-22.9	-19.2
Optical Sideband Power (point D)	dBm	-44.5	-40.3	-41.5	-45.4	-52.1	-54	-53.2	-49.6
SNR Single Sideband (point E/F)	dB	12.3	15.8	15.6	12.7	6.2	4.4	5.0	8.3
SNR Dual-Sideband	dB	13.5	16.8	16.9	14.5	8.3	6.5	7.0	10.0

7

How were the atmospheric conditions' effects on sub-THz propagation experimentally verified? Specifically, under high humidity, were there significant signal attenuation changes? How does the system design compensate for these variations?

We thank the reviewer for this comment.

We tracked the humidity and temperature during the experiment and calculated the expected attenuation according to ITU-R models to be between 3 and 5 dB on the day of the measurement (2nd of June 2023). In our system, these changes are compensated by the gain of the preamplifier EDFA, which was operated in power-controlled mode, therefore adjusting the gain to reach the optimal power into the coherent receiver.

We adjusted the manuscript in the following way:

Results:

[…] EDFA 3 has a noise figure of 4.5 dB and is operated in power-controlled mode, which keeps the input power into the coherent receiver stable. Both sidebands are converted to baseband […] (P.12, L.11)

[…] The sub-THz signal is transmitted over a 1400 m free-space distance, 2 m above a predominantly meadow terrain with an asphalt runway in between, to the receiver. The channel loss comprises 142.5 dB of FSPL and, based on measured conditions (12.5 °C, 86% relative humidity), 5-7 dB of atmospheric attenuation, which is partially compensated by two identical […] (P.11, L.25)

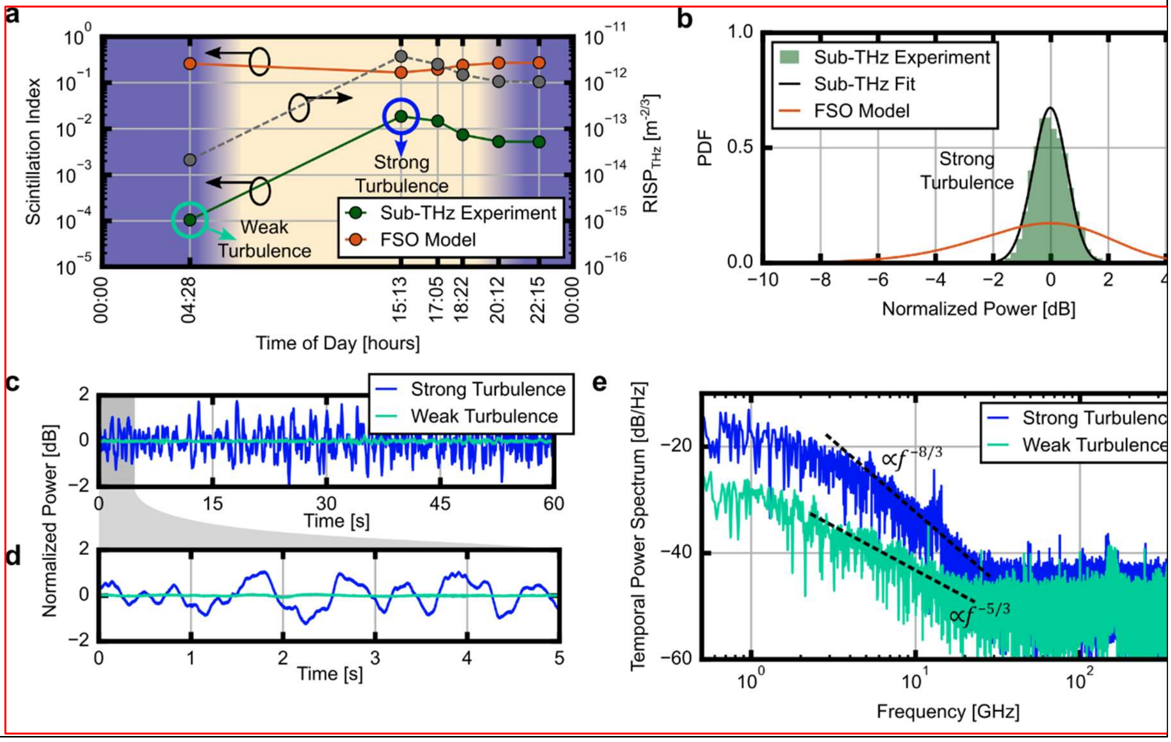

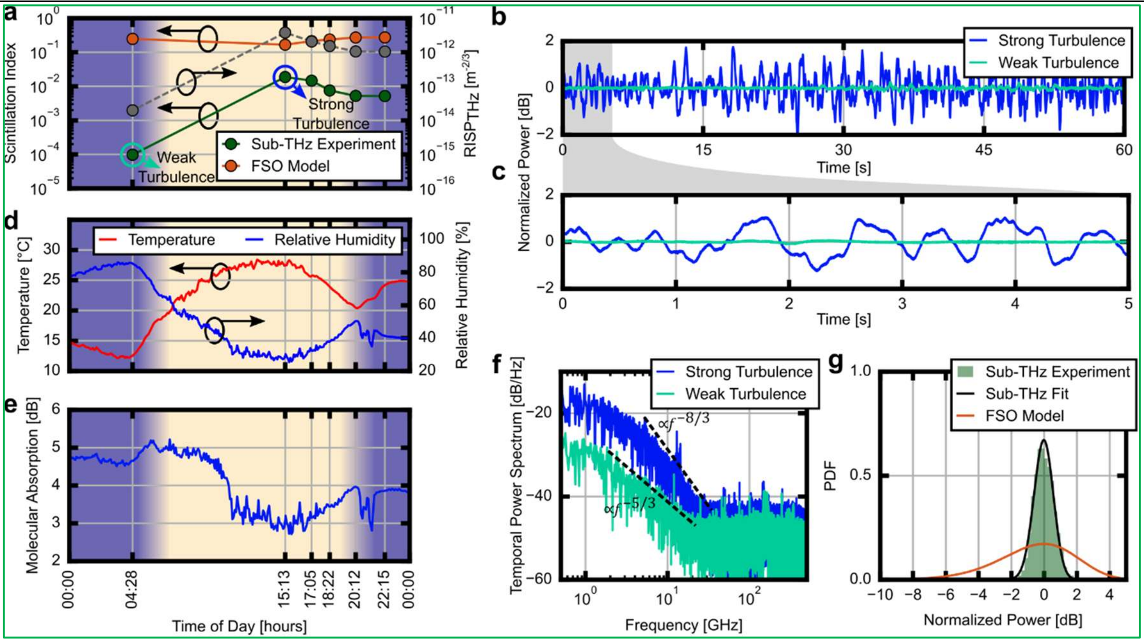

Fig. 7: Impact of atmospheric turbulence on sub-THz compared with FSO links. a *Scintillation index (SI) and refractive index structure parameter (RISP) of the sub-THz link measured over the course of the day taken on 2nd June 2023 compared with calculated SIs of the equivalent free-space optical (FSO) link for the same conditions. The time stamps indicate the times where scintillation measurements have been taken.* ***b*** *Recorded time traces of the normalized power for worst-case and best-case conditions,* ***c*** *time traces zoomed in to the first 5 seconds.* ***d*** *Measured temperature and humidity values at the transmitter and* ***e*** *estimated atmospheric attenuation according to ITU-R P.676.* ***f*** *Fourier transform of the measured normalized power bandpass filtered between 0.5 Hz and 500 kHz, and* ***g*** *Measured histogram of the normalized received power of the 1.4 km sub-THz link in worst-case turbulence conditions and fitted $\Gamma\Gamma$-model to extract a RISP of $3.92 \cdot 10^{-12}$ m$^{-2/3}$ (solid green line). The extracted RISP was used to calculate the expected probability density function (PDF) of a hypothetical 1.4 km FSO link operating at 1550 nm, showing much higher power fluctuations (solid red line).*

Fig. 7: Impact of atmospheric turbulence on sub-THz compared with FSO links. a *Scintillation index (SI) and refractive index structure parameter (RISP) of the sub-THz link measured over the course of the day taken on 2nd June 2023 compared with calculated SIs of the equivalent free-space optical (FSO) link for the same conditions.* ***b*** *Measured histogram of the normalized received power of the 1.4 km sub-THz link in worst-case turbulence conditions and fitted $\Gamma\Gamma$-model to extract a RISP of $3.92 \cdot 10^{-12}$ m$^{-2/3}$ (solid green line). The extracted RISP was used to calculate the expected probability density function (PDF) of a hypothetical 1.4 km FSO link operating at* 1550 nm*, showing much higher power fluctuations (solid red line).* ***c*** *Recorded time traces of the normalized power for worst-case and best-case conditions,* ***d*** *time traces zoomed in to the first 5 seconds, and* ***e*** *Fourier transform of the normalized power bandpass filtered between 0.5 Hz and 500 kHz.*

8

What are the bandwidth and operational frequency range of the UTC-PD and sub-THz amplifiers used in the system? Were the gain spectrum, frequency response, and attenuation characteristics of these components considered when selecting the central frequency? Is the chosen central frequency optimized for the system's best performance?

We thank the reviewer for this comment. Indeed, the carrier frequency was chosen to optimize the performance and is determined by the combined response of the UTC-PD and sub-THz amplifiers. We adjusted the manuscript in the following way:

Results:

[…] The carrier frequency of 226 GHz has been chosen to maximize the system's throughput and was limited by the combined bandwidth of the UTC-PD and the sub-THz amplifier. […] (P.11, L.21)

[…] amplifiers at these frequencies, see Supplementary Note 4. The dual-sideband […] (P.13, L.20)

Supplementary Information:

Supplementary Note 4: Sub-THz Components

Supplementary Figure 2 shows the normalized response and gain spectrum of the UTC-PD and sub-THz amplifiers used in the experiment. The high differences in output power across the band could be mitigated by splitting the band into 8 NFDM tributaries and perform power loading, see Supplementary Table 3. The optimal center frequency of 226 GHz was chosen to maximize the data rate through the channel.

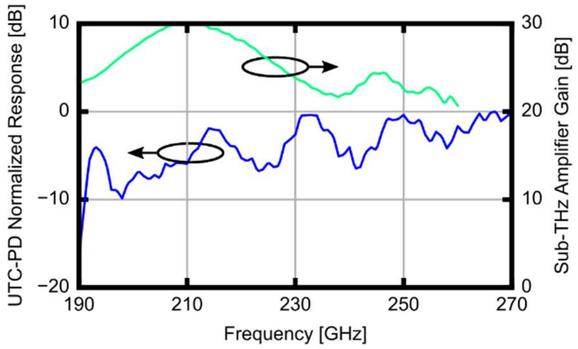

Supplementary Fig. 2: Normalized response of the sub-THz components. *The normalized response of the uni-travelling photodiode (UTC-PD) is shown in blue while the gain spectrum of the sub-THz amplifier is shown in green.*

9

The authors state, "since the free-space channel is linear, only linear equalizers were required." However, this is not true. Other system components could also introduce nonlinear distortions? There is no discussion of nonlinear processing in the DSP section. Given the use of high-gain sub-THz amplifiers, could nonlinearities be introduced in these components? If so, how are these nonlinear effects addressed or mitigated in the system?

We thank the reviewer very much for the comment. We totally agree with the reviewer that amplifiers can introduce severe non-linearities, especially when driven close to saturation. In our case we were below saturation and do not expect any severe non-linearities. This is underlined by two instances in the measured IQ diagrams.

- The symbols are equally spaced with gaussian noise which points away from non-linearities.
- Applying a Volterra algorithm with 1 second and 1 third-order tap does not improve the SNR again pointing away from non-linearities.

We added the following sentences to the manuscript to point this out more clearly.

Results:
[…] Combining the two baseband signals was achieved by utilizing a butterfly extension of the FFE. Despite the two sub-THz amplifiers in the link, non-linear equalization could not improve the SNR and BER as the amplifiers were not operated close to saturation. Therefore, non-linear equalization was omitted, reducing the computational complexity of the DSP. […] (P.13, L.9)

10

Deploying sub-THz links will require infrastructure such as transmitter/receiver installations, antenna alignment systems, and power supplies. What are the practical challenges and requirements for deploying these links in various environments? How can the process be streamlined to support large-scale rollouts for 6G networks?

We thank the reviewer for this comment.

For kilometer range point-to-point communication, high-gain antennas with low beam divergence and small half-power beam widths in the range of 0.2 degrees are required. One of the main challenges are:

- Aligning the antennas to this precision requires motorized alignment and, in our case, periodic realignment of the antennas.
- Protection of the antenna from the elements (wind, snow, ice) as contamination and movement will have higher penalties compared to lower frequencies.
- RF Packaging of the plasmonic devices to reduce the setup complexity.

We adjusted the manuscript in the following manner:

Discussion:

[…] systems, concluding that sub-THz offers high-capacity, low-complexity connectivity with enhanced robustness against adverse weather. The main challenges to overcome and allow for long-term deployment is the precise and periodic realignment of the antennas with 0.1 degrees precision and RF-packaging of the plasmonic modulator. Packaging techniques with only 0.8 dB of insertion loss over 70 GHz of bandwidth are available[53], which would improve the link budget and at the same time allow for a simple setup. Our transparent […] (P.17, L.33)

11

The paper uses RF probes for signal modulation, rather than packaged devices. What methods are planned for packaging these devices in the future? What bandwidth losses are anticipated in the packaging process, and are there specific techniques to minimize degradation while maintaining performance?

We thank the reviewer for this interesting question. RF packaging of the plasmonic modulator is necessary to make large scale deployment possible.

One packaging technique could be by coupling from a hollow metal waveguide to the chip by an on-chip antenna, as demonstrated in [1]. Between 200 and 270 GHz a loss of 0.8 dB could be expected. This is a lower loss compared to the loss of the probe we currently use, which is specified to be 2.3 dB. Therefore, packaging the device can potentially improve the performance, without significantly limiting the bandwidth.

A. Zamora, K. M. K. H. Leong, T. Reck, G. Chattopadhyay, and W. Deal, "A 170-280 GHz InP HEMT low noise amplifier," *Int. Conf. Infrared, Millimeter, Terahertz Waves, IRMMW-THz*, pp. 1–2, 2014, doi: 10.1109/IRMMW-THz.2014.6956402.

We adjusted the manuscript in the following manner:

Discussion:

[…] systems, concluding that sub-THz offers high-capacity, low-complexity connectivity with enhanced robustness against adverse weather. The main challenges to overcome and allow for long-term deployment is the precise and periodic realignment of the antennas with 0.1 degrees precision and RF-packaging of the plasmonic modulator. Packaging techniques with only 0.8 dB of insertion loss over 70 GHz of bandwidth are available[53], which would improve the link budget and at the same time allow for a simple setup. Our transparent […] (P.17, L.33)

12

The paper describes two photoelectric conversions, and from Figure 4, it appears that the two optical signals at the UTC-PD are not phase-locked. Similarly, the optical signals at the

coherent receiver (Coh.Rx) also operate independently. This would result in poor phase noise in the electrical signals. Does the offline signal processing section address phase noise mitigation?

We agree with the reviewer, that the two free-running lasers at the transmitter and receiver introduce phase noise. Yes, the phase noise mitigation is done using blind phase search (BPS) phase recovery. We could not observe a SNR penalty.

We adjusted the manuscript in the following manner:

Results:
[…] carrier recovery, blind phase search (BPS) and a T/2-spaced feedforward equalizer (FFE). […] (P.11, L.36)

[…] Furthermore, phase noise introduced by the free-running lasers could be corrected by the BPS, without any significant penalty. The resulting baseband power spectral density is shown […] (P.13, L.5)

13

The demonstrated system has a low bandwidth utilization of the ultra-wideband MZM, and to improve the SNR, two coherent receivers were used. This trade-off in cost versus performance needs to be carefully analyzed. Could the system be scaled to higher THz center frequencies?

(1) With respect to the bandwidth utilization
We agree with the reviewer, that the full bandwidth of the MZM is not utilized. To date, the bandwidth of our system is primarily limited by the combined spectrum of the following components:

- The packaged UTC-PD
- Sub-THz amplifiers
- The WR-03 hollow metal waveguide of the packaged components.

More fundamentally, the bandwidth is additional limited by the atmospheric attenuation, see Figure 3. We could improve the bandwidth with future amplifier developments and by using more broadband high-speed PDs.

(2) With respect to the higher THz center frequencies
The system could be scaled to higher frequencies as the plasmonic modulators work up to 1 THz. However, for kilometer-range communication there is no value in going to higher THz frequencies due to the higher free space attenuation losses (see Figure 2a). Furthermore, current THz electrical amplifiers and source have less gain and power output at higher frequencies which would further limit the reach. However, due to the broadband nature of the modulator it is possible to switch operation to lower carrier frequencies for example to achieve better SNR in adverse weather conditions.

(3) With respect to the cost of the system
Regarding cost, we agree with the reviewer that the SNR gain of 2 dB per channel comes at a cost of doubling the amount of coherent receivers. The precise analysis of the cost-throughput trade-off in operation depends on many variables which are not available to us and would be beyond the scope of this paper. However, we added the sentence to the manuscript to note the problem:

[…] which is a 10 % increase in capacity compared to single sideband reception, which comes at the cost of one additional coherent receiver and wavelength demultiplexing. More precisely, the single-received LSB […] (P.13, L.32)

14

It is recommended to provide key THz device parameters, such as operating frequency and bandwidth, to enhance the clarity of the demonstration.

We thank the reviewer for the comment and added the response of the UTC-PD and gain of the sub-THz amplifier.

We adjusted the manuscript in the following way:

Results:

[…] amplifiers at these frequencies, see Supplementary Note 4. The dual-sideband […] (P.13, L.120)

Supplementary Information:

Supplementary Note 4: Sub-THz Components

Supplementary Figure 2 shows the normalized response and gain spectrum of the UTC-PD and sub-THz amplifiers used in the experiment. The high differences in output power across the band could be mitigated by splitting the band into 8 NFDM tributaries and perform power loading, see Supplementary Table 3. The optimal center frequency of 226 GHz was chosen to maximize the data rate through the channel.

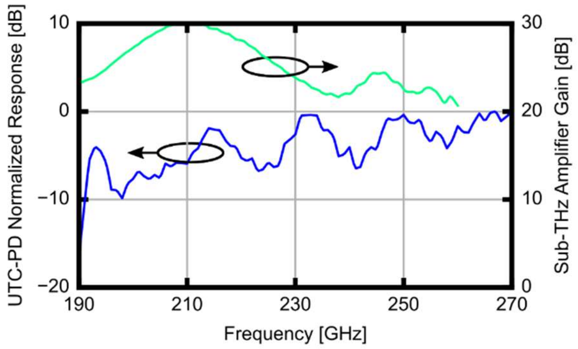

Supplementary Fig. 2: Normalized response of the sub-THz components. *The normalized response of the uni-travelling photodiode (UTC-PD) is shown in blue while the gain spectrum of the sub-THz amplifier is shown in green.*

15

Some typos: please check your definitions of up-mixer and down mixer. For example, the descriptions in Figure 4(a) are inconsistent with the subtitles. Should "Plasmonic Sub-THz Down-Mixer" be "Plasmonic Sub-THz Up-Mixer"?

We thank the reviewer for catching these typos. We corrected the one pointed out and re-checked the manuscript for any typos and inconsistency.

We thank the reviewer for all comments and interesting questions. We hope that the paper communicates the innovation and advancements over the state-of-the-art more clearly. We showed that the all-photonic approach using high-speed modulators (like plasmonic modulators) offers highest bandwidth and therefore data rate and can be used for kilometer ranged communication. The transition from isolated lab-based experiments to large-scale integrated field trials highlights both the potential and the challenges of this technology for future industrial applications.

Reviewer 3

1

The authors present a photonic-plasmonic sub-THz wireless link and achieve 160-Gbps transmission over 1400 m. This result is remarkable; however, it is similar to the work presented at OFC 2024. It would be beneficial if the authors could extend this research further. The authors have added discussions and comparisons with the FSO system; however, no solutions are proposed to overcome the limitations of each technology. Additionally, the two works presented in the manuscript are not closely related to each other. It would be better to separate them into two papers, each with extended results.

We thank the reviewer for his overall positive assessment are happy to hear that the reviewer finds the result remarkable.

(1) With respect to splitting the work into two papers:
We think that keeping the content in one paper helps to compare results and therefore suggest keeping it because we have to disagree that the two works are not closely related. Future communication systems will rely on fiber optics, FSO and wireless technologies. A potential solution to overcome the limitations of each technology is to employ both technologies complementary to each other as we tried to outline in Figure 1 b, c. The here demonstrated work shows how we can switch from the optical domain to the sub-THz domain and vice-versa while keeping the same established optical Rx and Tx.

We clarify this further in the main text with the following changes:

Results:

[…] The seamless transition from the optical domain to the sub-THz domain is enabled by the high-speed sub-THz down- und up-mixer as illustrated in Figure 1c. In case of adverse weather conditions, where FSO is blocked, the optical signal at the transmitter side can be re-routed to the sub-THz down-mixer and transmitted via a sub-THz carrier. On the receiver side, the signal is directly up-mixed back to the optical domain by means of a high-speed plasmonic modulator. […]

[…] A high-speed sub-THz down- and up-mixer enables a seamless transition between the optical and sub-THz domains, as shown in Figure 1c. During adverse weather conditions that disrupt free-space optical (FSO) links, the optical signal at the transmitter can be rerouted through the sub-THz down-mixer and sent over a sub-THz carrier. At the receiver, the signal is directly up-mixed back into the optical domain using a high-speed plasmonic modulator. This seamless switching capability allows the optical and sub-THz links to complement each other in a confluent way, effectively addressing the limitations of each technology. […] (P.5, L.21)

2

It is evident that RF systems, including those in the THz band, are robust to atmospheric turbulence due to the nature of signal reception. RF signals are received using large-diameter antennas. In contrast, FSO systems are more affected by atmospheric turbulence due to the use of very small-diameter photodetectors for signal detection. It is also well known that aperture averaging with a large-diameter lens can reduce the effects of turbulence. This effect is particularly significant when using direct fiber coupling at the receiver, as the incoming free-space signals are directly focused into a 10-μm core fiber. The turbulence can alter the angle of arrival of these signals. Given these factors, it is clear that the impact of turbulence in RF systems is minimal.

We thank the reviewer for this remark. Indeed, the fact that the impact of atmospheric turbulences for RF systems is smaller compared to FSO is already known and has been demonstrated in laboratory based studies in turbulence chambers. We believe that measurements of the effect of atmospheric turbulence in a field trial transmitting high data rates over kilometer distances, as one would encounter in a deployed link, are valuable to the community.

We adjusted the abstract and introduction to stress this point in the following way:

Abstract:

[…] We assessed power variations due to atmospheric turbulence; the scintillation index remained below 0.019 under strong turbulence, confirming sub-THz link resilience. A theoretical comparison […] (P.1, L.26)

Introduction:

[…] We report outdoor measurements of atmospheric turbulence effects on a high data rate sub-THz link, with scintillation indices staying below 0.019 even under strong turbulence. These results confirm the known resilience of sub-THz links to turbulence[34, 35] and, unlike many lab-based studies, provide valuable evidence to the community that sub-THz links perform reliably under real-world outdoor conditions over practical distances exceeding 1 km. For the first time outside laboratory conditions, we measured the impact of atmospheric turbulence on received sub-THz power, with the scintillation index remaining below 0.019 even in strong turbulence. […] (P.3, L.40)

3

For the data shown in Fig. 7, why does the scintillation index for the FSO system remain unchanged during the measurement period, while the scintillation index for the THz system varies? What were the weather conditions and temperature at the time of the measurement? Typically, turbulence is stronger in hot weather, and it is usually most intense around noon. If the scintillation index (SI) of the FSO system does not change, it indicates that the atmospheric turbulence remained relatively constant during the measurement period. In that case, we cannot conclude that the experiment was conducted under strong turbulence conditions, as stated in the paper.

We thank the reviewer for this interesting comment. Indeed, we measured the humidity and temperature during the day of the measurements and could observe that the measured scintillation index of the sub-THz link follows the trend very well, peaking in the afternoon, where temperature was highest.

Regarding the scintillation index of the FSO, we want to stress that these values are not measured but calculated according to the ΓΓ-model. The details of the calculation are written in the supplementary information. According to the ΓΓ-model as shown in Figure 4.a, the scintillation index can indeed become smaller even when RISP or distance are increased, or the wavelength is reduced. This matches with other publications such as:

W. Gao, C. Han, and Z. Chen, “Scintillation and Attenuation Modelling of Atmospheric Turbulence for Terahertz UAV Channels.”

In very strong turbulence, the optical wavefront becomes so distorted that spatial coherence is lost, leading to reduced interference effects and thus a decrease in scintillation.

We adjusted the manuscript in the following way:

Results:

[…] to sub-THz link in strong turbulence. Interestingly, the SI of the theoretical FSO link is not proportional to the RISP values, indicating that the saturation regime is reached, which

can also be observed in Figure 4a. Figure 7b shows the probabilistic density function of the […] (P.17, L.19)

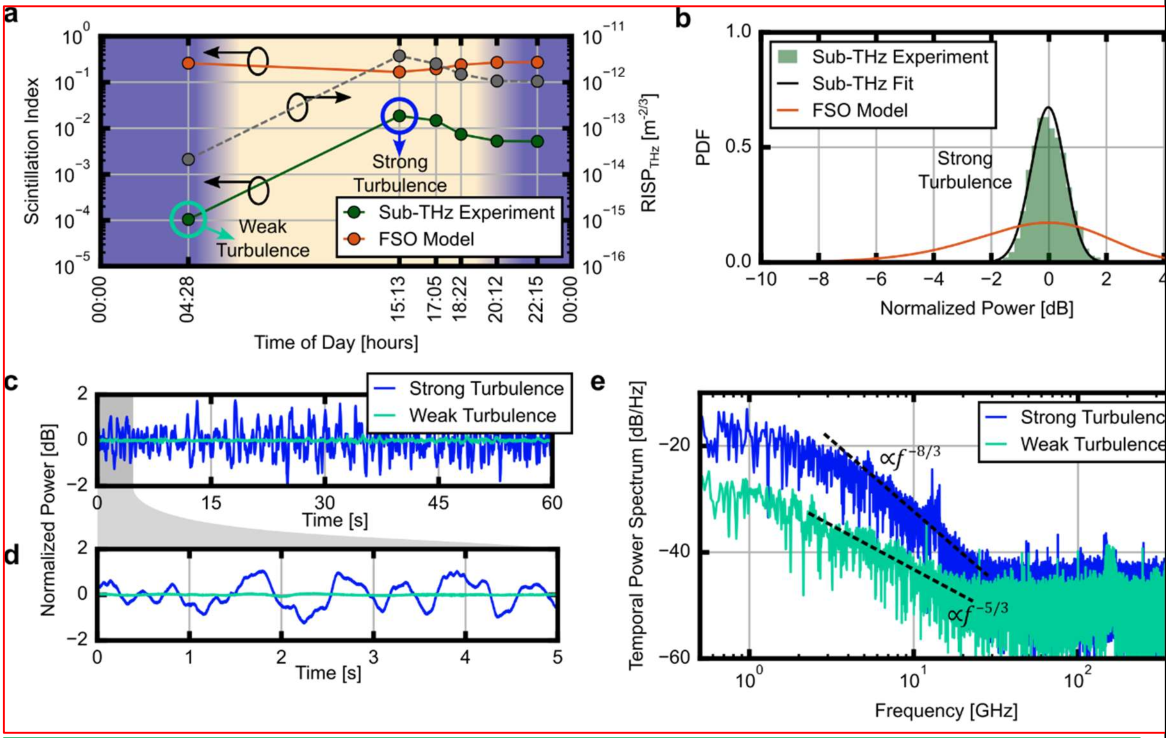

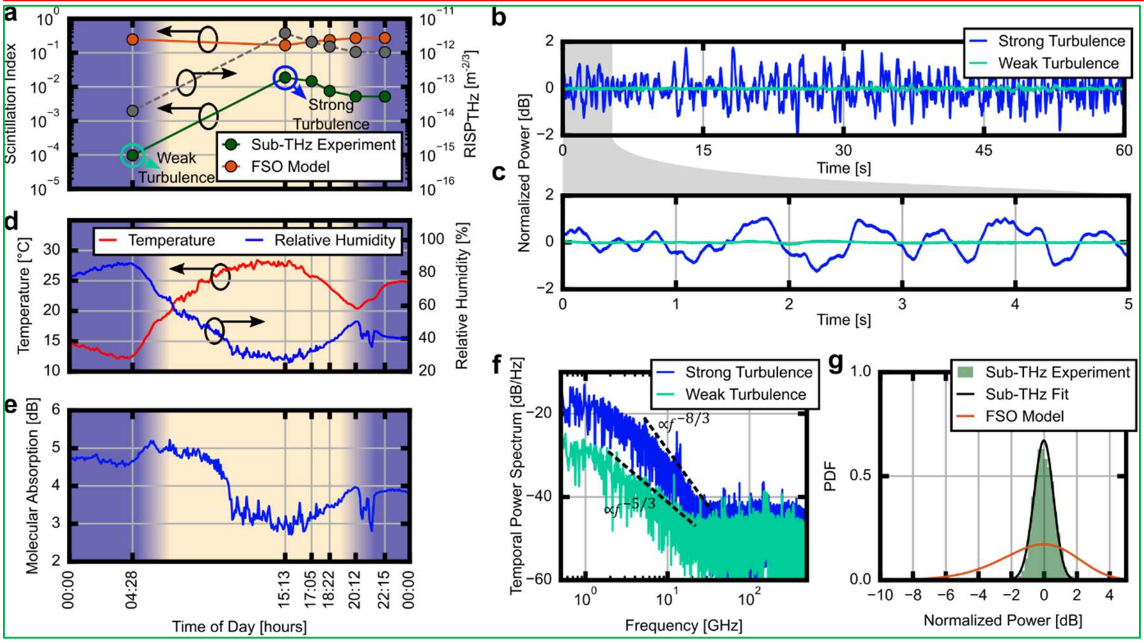

Fig. 7: Impact of atmospheric turbulence on sub-THz compared with FSO links. a *Scintillation index (SI) and refractive index structure parameter (RISP) of the sub-THz link measured over the course of the day taken on 2nd June 2023 compared with calculated SIs of the equivalent free-space optical (FSO) link for the same conditions. The time stamps indicate the times where scintillation measurements have been taken.* ***b*** *Recorded time traces of the normalized power for worst-case and best-case conditions,* ***c*** *time traces zoomed in to the first 5 seconds.* ***d*** *Measured temperature and humidity values at the transmitter and* ***e*** *estimated atmospheric attenuation according to ITU-R P.676.* ***f*** *Fourier transform of the measured normalized power bandpass filtered between 0.5 Hz and 500 kHz, and* ***g*** *Measured histogram of the normalized received power of the 1.4 km sub-THz link in worst-case turbulence conditions and fitted $\Gamma\Gamma$-model to extract a RISP of $3.92 \cdot 10^{-12}$ $m^{-2/3}$ (solid green line). The extracted RISP was used to calculate the expected probability density function (PDF) of a hypothetical 1.4 km FSO link operating at 1550 nm, showing much higher power fluctuations (solid red line).*

***Fig. 7: Impact of atmospheric turbulence on sub-THz compared with FSO links. a** Scintillation index (SI) and refractive index structure parameter (RISP) of the sub-THz link measured over the course of the day taken on 2nd June 2023 compared with calculated SIs of the equivalent free-space optical (FSO) link for the same conditions. **b** Measured histogram of the normalized received power of the 1.4 km sub-THz link in worst-case turbulence conditions and fitted $\Gamma\Gamma$-model to extract a RISP of $3.92 \cdot 10^{-12}$ $m^{-2/3}$ (solid green line). The extracted RISP was used to calculate the expected probability density function (PDF) of a hypothetical 1.4 km FSO link operating at 1550 nm, showing much higher power fluctuations (solid red line). **c** Recorded time traces of the normalized power for worst-case and best-case conditions, **d** time traces zoomed in to the first 5 seconds, and **e** Fourier transform of the normalized power bandpass filtered between 0.5 Hz and 500 kHz.*

4

The change in the scintillation index (SI) of the THz system could be due to other factors, such as wind effects, power fluctuations in signal generation and transmission, and the overall stability of the systems. It is recommended that additional measurements be taken over multiple days and under varying weather conditions.

We agree with the reviewer, that it would be beneficial to measure the effects of turbulence and attenuation over multiple days in various weather conditions. Unfortunately, our time on the field was limited and we could only measure over the course of one day in sunny conditions.

To rule out effects due to power fluctuation in signal generation, transmission and overall stability, we have indeed performed short distance indoor experiments to confirm the stability of the system before going to the field trial. Some of these short distance results have been published by members of our group:

Horst Y, *et al.* Transparent Optical-THz-Optical Link at 240/192 Gbit/s Over 5/115 m Enabled by Plasmonics. *Journal of Lightwave Technology* **40**, 1690-1697 (2022).

Power fluctuations from wind-induced mechanical resonances, normally appear as narrowband peaks in the power spectrum. One strong peak at approximately 10 Hz is visible in our measurements (see Figure 7e.), which is in the range where we would expect some mechanical resonances. We couldn't observe any similar fluctuations in short distance experiments.

Furthermore, we attributed the fluctuations of the received power to scintillation due to following reasons:

- The temporal power spectrum follows the Kolmogorov power-law spectrum, which is expected for scintillation effects.
- The strength of the power fluctuations strongly depends on the time of day and the temperature and humidity, which we measured on site and have illustrated in the supplementary information. We observe high correlation between the temperature and measured scintillation index.

We adjusted the manuscript in the following way:

Results:

[…] instances during the day using a power meter with an averaging time of 200 μs. To confirm that the origin of the power fluctuations stem from the free-space channel, short range indoor experiments in the laboratory over 5 m have been conducted in prior studies[32], showing that the received power is stable. Therefore, the power fluctuations in this study are attributed to atmospheric turbulence effects. We use a $\Gamma\Gamma$-distribution and Andrew's method[47] to extract the […] (P15, L.8)

5

It is recommended that additional measurements be taken over multiple days and under varying weather conditions. Additionally, more data should be recorded and analyzed throughout a single day. A comparison of measurements taken over the same link would help reach a more precise conclusion.

	We thank the reviewer for his recommendation. While beyond the scope of this paper, it would indeed be preferable that future studies could collect more data over a longer period and in different weather conditions. Therefore, future work is planned where fiber, FSO and sub-THz are simultaneously deployed over longer periods of times. However, at the moment this is not possible due to lack of sub-THz packaged plasmonic modulator, which would allow for long-term measurements.<br><br>We adjusted the manuscript in the following way:
	Discussion:<br><br>[…] systems, concluding that sub-THz offers high-capacity, low-complexity connectivity with enhanced robustness against adverse weather. The main challenges to overcome and allow for long-term deployment is the precise and periodic realignment of the antennas with 0.1 degrees precision and RF-packaging of the plasmonic modulator. Packaging techniques with only 0.8 dB of insertion loss over 70 GHz of bandwidth are available[53], which would improve the link budget and at the same time allow for a simple setup. Our transparent […] (P.17, L.33)<br><br>[…] effects on a THz link, showing low scintillation index of 0.019 even under strong turbulence. The promising performance observed in this study highlights the need for future work involving long-term sub-THz link deployment and assessment of BER and EVM across diverse weather conditions. […] (P.17, L.43)

We thank the reviewer for the valuable feedback. We hope that with the paper can paint a more cohesive picture and relate the different technologies (fiber optics, FSO, sub-THz) to each other. We believe that combined FSO and sub-THz links leverage its respective advantages and disadvantages very well. We also believe that the all-photonic approach enables for a more confluent integration of both technologies. Furthermore, we plan to extend the research further to study long-term measurements in different weather conditions.

Eidgenössische Technische Hochschule Zürich
Swiss Federal Institute of Technology Zurich

Institute of Electromagnetic Fields (IEF)

ETH Zurich
M.Sc. Boris Vukovic
PhD Student
ETZ K 93
Gloriastrasse 35
8092 Zurich, Switzerland

Email: boris.vukovic@ief.ee.ethz.ch
Web: http://www.ief.ee.ethz.ch/

Zurich, 11 February 2026

Third Round of Revisions on Nature Communications Manuscript NCOMMS-24-85013C

Dear Reviewers,

On behalf of all the co-authors I thank you for the supportive comments and thorough review, which enabled us to improve the quality of the paper even further. All your comments have been thoroughly addressed, and corresponding changes have been made to the manuscript. Below are detailed responses to each of the reviewer's comments (*italic gray*). Additionally, alterations made to the initial manuscript are clearly marked in red (~~removal~~) or green (addition) which are directly added below each comment.

Yours sincerely,

Boris Vukovic

Revisions Round 3

Reviewer 1

We thank the reviewer for his assessment and answer below the reviewer's comments in detail to clarify these open points:

The corrections and suggestions were taken into account, but one thing is not technically clear in my understanding of the paper.

Authors said that output power at point C (Figure 3) is 12 dBm, ie 3 dB beyond amplifier P1dB which is 9 dBm. Operating a QAM-16 3 dB beyond P1dB induces that the QAM-16 signal should be compressed a lot, which is not the case. This is surprising as any-non linear equalization is done. Authors should cross-check before publication.

We thank the reviewer for this insightful comment.

We have carefully re-examined the output power and confirm that it is indeed 12 dBm. Upon further inspection of the amplifier data sheet, we identified a plausible explanation for the observed low distortion in the 16-QAM tributaries. Specifically, the amplifier's 1 dB compression point (P1dB) is frequency dependent and lies between 10 and 12 dBm in the 190–220 GHz range. This frequency range coincides with that used for the higher-order modulation formats (16-QAM and 8-QAM).

We therefore believe that, for tributaries 1–4, the amplifier operates close to but below the onset of significant nonlinear distortion, such that no nonlinear compensation is required.

We have revised the manuscript as follows:

Manuscript:

[…]. Tributaries 5-8 have diminished powers, attributable to the reduced output power of the UTC-PD and sub-THz amplifiers at these frequencies, see Supplementary Note 5. By contrast, tributaries 1–4 operate in a frequency range where the sub-THz amplifier exhibits a higher saturated output power and a higher 1 dB compression point (P1dB), see Supplementary Fig. 6. This operating regime helps to suppress nonlinear distortion and supports the use of higher-order modulation formats. The dual-sideband reception was particularly efficient in improving these tributaries with a low SNR. […] (P.8, L.27)

Supplementary:

[…]. The optimal center frequency of 226 GHz was chosen to maximize the data rate through the channel. Supplementary Figure 6 shows the typical frequency-dependent saturated output power and 1 dB compression point (P1dB) of the sub-THz amplifiers, provided by Virginia Diodes[18]. As P1dB increases at lower carrier frequencies, distortion is reduced, enabling the use of higher-order modulation formats for tributaries 1–4, see Figure 4. (P.8, L.11)

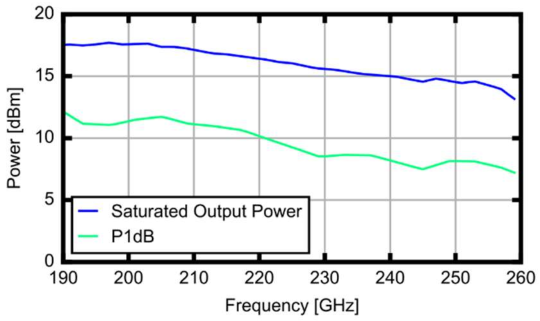

Supplementary Fig. 6: Saturated output power and 1 dB compression point (P1dB) of the sub-THz amplifier. *The values shown represent typical device performance rather than that of the specific amplifier used in this work and are provided by VDI*[18].

Reviewer 2

We thank the reviewer for his assessment and answer the reviewer's comments below in detail to clarify these open points:

The revised manuscript title now better highlights the technical innovation. The overall quality paper after revision has been improved a lot. However, I suggest further removing "for 6G Networks". Since the use of the sub-THz band in 6G is not yet definitively standardized and the demonstrated modulator/link represent the physical-layer advancement, a more general title would avoid limiting the work's applicability. Additionally, to maintain consistency throughout the manuscript and to ensure the focus remains on the innovative concepts, I recommend that the authors also revise any similar statements in the abstract and main text that may overly associate this work exclusively with 6G.

We thank the reviewer for the insightful comment. We agree that the physical-layer innovations discussed in this work are broader than 6G, which is still under standardization. Accordingly, we have revised the title and the manuscript as follows:

Title:

~~Plasmonic Modulator Enabling Kilometer-Range, High-Throughput Sub-THz Links for 6G Networks~~

Plasmonic Modulator Enabling Kilometer-Range High-Throughput Sub-THz Links for Radio Access Networks

Abstract:

[…] Future ~~6G~~ radio access networks must accommodate growing mobile data traffic. […] and cost-effective deployment for ~~6G~~ wireless front- and backhaul.

Manuscript:

In ~~5G/6G~~ future mobile radio access networks (RAN), the links between the user end (UE) […] (P.1, L.33)

All-photonic sub-THz links could provide a cost-effective and easily integrable alternative to fiber for the dense ~~6G~~ radio access networks of the future. […] (P.3, L.18)

[…]. The vision of this work is depicted in Figure 1, where the RAN integrates various communication systems, including fiber-based, ~~5G/6G~~ radio, free-space optical (FSO), and sub-THz systems. […] (P.4, L.6)

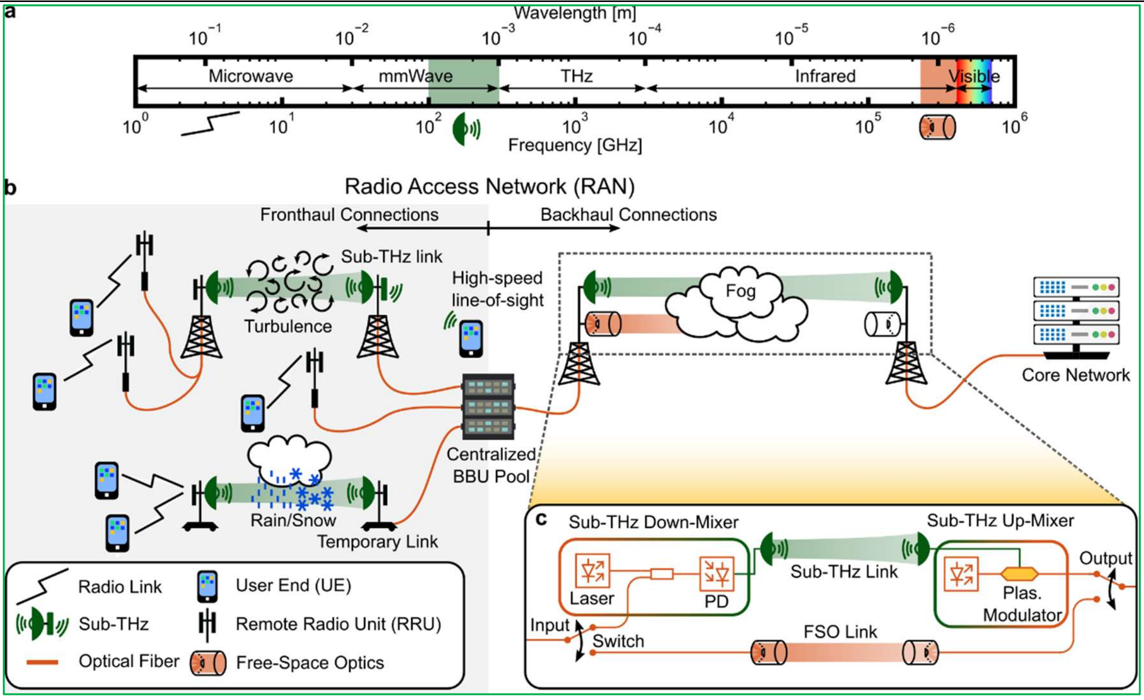

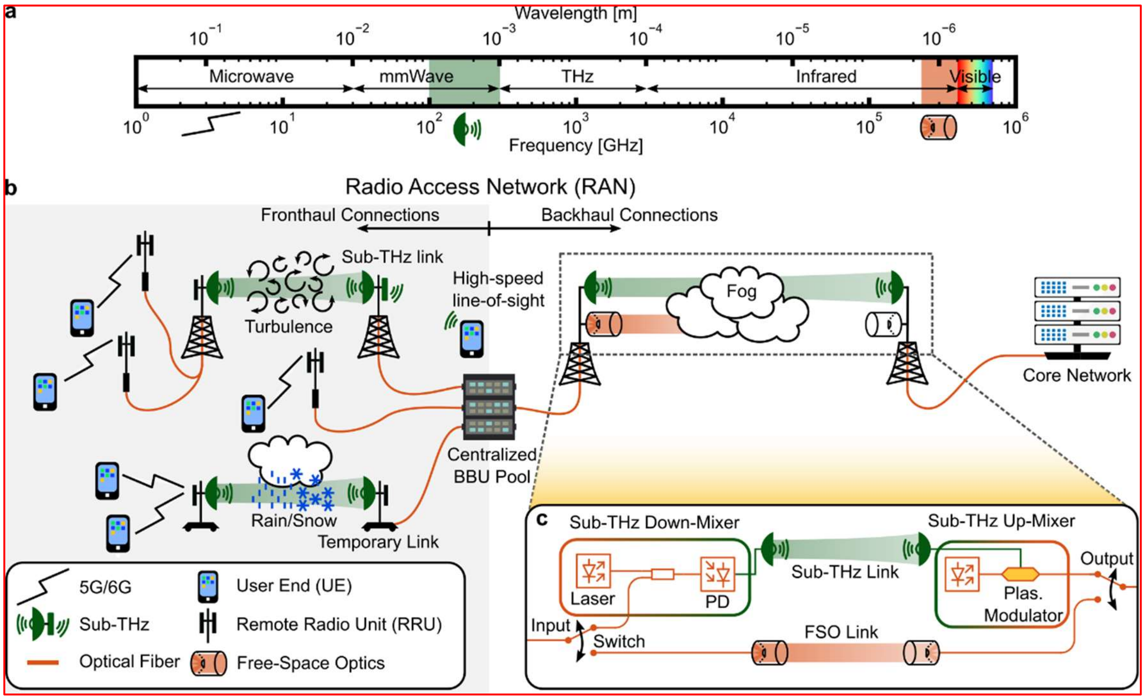

Fig. 1: **High-capacity sub-THz front- and backhaul connections for radio access networks as envisioned for future ~~6G~~ mobile communications.** […] (P.4, L.15)

[…]. All-photonic sub-THz links offer high data rates in the kilometer range, making them a viable solution for backhauling in ~~6G~~ future radio access networks. (P.13, L.18)

Reviewer 3

We thank the reviewer for his assessment and answer below the reviewer's comments in detail to clarify these open points:

The authors have made efforts to improve the manuscript in response to the reviewer's previous comments. By moving the "Comparison of Sub-THz and FSO Communications" section to the Supplementary Information and revising the title, the manuscript is now more focused and easier for readers to follow.

Although the authors disagreed with some of the reviewer's earlier comments, the reviewer's intention was not to impose additional burdens or challenges, but rather to help strengthen the manuscript. If the authors and the editor consider the current content, largely based on the OFC 2024 paper, sufficient for publication in Nature Communications, the reviewer has no objection.

We thank the reviewer for the detailed and constructive feedback. We viewed the comments not as burdens or challenges, but as valuable suggestions that helped us further improve the quality of the manuscript. We appreciate the reviewer's positive assessment of the revisions and are grateful that no further concerns were raised.

Minor comments on the revised manuscript:
- The section titles "Influence of Atmospheric Turbulence on Wireless Links" (main text) and "Influence of Atmospheric Turbulence on Sub-THz Links" (Supplementary Note 2) are very similar. The authors are encouraged to revise one of these titles to better distinguish them.

We thank the reviewer for this remark. We changed the title of the subsection in the manuscript and the supplementary information to better distinguish them:

Manuscript:

~~Influence of Atmospheric Turbulence on Wireless Links~~

Influence of Atmospheric Turbulence on Sub-THz Links

Beyond the data transmission over large distances, we further […] (P.10, L.6)

Supplementary:

~~Supplementary Note 2: Influence of Atmospheric Turbulence on Sub-THz Links~~

Supplementary Note 2: Modeling of Turbulence-Induced Impairments in Sub-THz Wireless Links

Atmospheric turbulence is air movement caused by wind […] (P.4, L.4)

- The "Discussion" section in the main text is quite brief. It would be helpful to include additional details on packaging approaches and the development of the modulator module to support practical implementation.

We thank the reviewer for this comment and agree that the packaging discussion could be expanded. We therefore added the following section and references to the manuscript, explaining how a sub-THz package of a plasmonic modulator might be implemented.

References

47. Zamora A, Leong KMKH, Reck T, Chattopadhyay G, Deal W. A 170-280 GHz InP HEMT low noise amplifier. *International Conference on Infrared, Millimeter, and Terahertz Waves, IRMMW-THz*, 1-2 (2014).

48. Leong KMKH*, et al.* A 340-380 GHz Integrated CB-CPW-to-Waveguide Transition for Sub Millimeter-Wave MMIC Packaging. *Ieee Microwave and Wireless Components Letters* **19**, 413-415 (2009).

49. Ibili H*, et al.* Plasmonic On-Chip Antenna Enabling Fully Passive sub-THz-to-Optical Receiver for Future RoF Systems. In: *Optical Fiber Communication Conference (OFC) 2024*). Optica Publishing Group (2024).

50. Ibili H*, et al.* Modeling Plasmonic Antennas for the Millimeterwave & THz Range. *IEEE Journal of Selected Topics in Quantum Electronics* **29**, 1-15 (2023).

Manuscript:

[…]. The main challenges to overcome and allow for long-term deployment ~~is~~ are the precise and periodic realignment of the antennas with 0.1 degrees precision and RF-packaging of the plasmonic modulator. In this work, the plasmonic modulator was probed using a sensitive RF probe, rendering the experimental setup susceptible to vibrations and environmental exposure. These limitations could be mitigated in future implementations through device packaging. For other sub-THz components, low-loss coupling from a rectangular waveguide to an on-chip transmission line has been demonstrated using an on-chip dipole antenna positioned at a quarter-wavelength from the waveguide termination. This technique is established in sub-THz amplifier packaging[46, 47] and insertion losses of 1 dB up to 380 GHz are achievable[48]. Plasmonic modulators have been directly integrated with on-chip antennas, enabling efficient capture of the incident RF field and direct electrical driving of the modulator[30, 49, 50]. Taking together, these established approaches indicate a pathway toward packaged plasmonic modulators with similarly low insertion loss, although such an implementation has not yet been experimentally demonstrated. ~~Packaging techniques with only 0.8 dB of insertion loss over 70 GHz of bandwidth are available[47], which would improve the link budget and at the same time allow for a simple setup.~~ Within this broader context, our transparent, all-photonic link, enabled by plasmonic modulators […] (P.13, L.6)
